# Multi-parametric quantitative *in vivo* spinal cord MRI with unified signal readout and image denoising

**DOI:** 10.1016/j.neuroimage.2020.116884

**Published:** 2020-08-15

**Authors:** Francesco Grussu, Marco Battiston, Jelle Veraart, Torben Schneider, Julien Cohen-Adad, Timothy M. Shepherd, Daniel C. Alexander, Els Fieremans, Dmitry S. Novikov, Claudia A.M. Gandini Wheeler-Kingshott

**Affiliations:** a Queen Square MS Centre, UCL Queen Square Institute of Neurology, Faculty of Brain Sciences, University College London, London, UK; b Centre for Medical Image Computing, Department of Computer Science, University College London, London, UK; c Center for Biomedical Imaging, Department of Radiology, New York University School of Medicine, New York, USA; d Philips UK, Surrey, England, UK; e NeuroPoly Lab, Institute of Biomedical Engineering, Polytechnique Montreal, Montreal, Canada; f Functional Neuroimaging Unit, CRIUGM, Université de Montréal, Montreal, Canada; g Brain MRI 3T Research Centre, IRCCS Mondino Foundation, Pavia, Italy; h Department of Brain and Behavioural Sciences, University of Pavia, Pavia, Italy

**Keywords:** Spinal cord, Signal readout, Quantitative MRI, Multi-parametric MRI, Marchenko-Pastur PCA denoising

## Abstract

Multi-parametric quantitative MRI (qMRI) of the spinal cord is a promising non-invasive tool to probe early microstructural damage in neurological disorders. It is usually performed in vivo by combining acquisitions with multiple signal readouts, which exhibit different thermal noise levels, geometrical distortions and susceptibility to physiological noise. This ultimately hinders joint multi-contrast modelling and makes the geometric correspondence of parametric maps challenging. We propose an approach to overcome these limitations, by implementing state-of-the-art microstructural MRI of the spinal cord with a unified signal readout in vivo (i.e. with matched spatial encoding parameters across a range of imaging contrasts). We base our acquisition on single-shot echo planar imaging with reduced field-of-view, and obtain data from two different vendors (vendor 1: Philips Achieva; vendor 2: Siemens Prisma). Importantly, the unified acquisition allows us to compare signal and noise across contrasts, thus enabling overall quality enhancement via multi-contrast image denoising methods. As a proof-of-concept, here we provide a demonstration with one such method, known as Marchenko-Pastur (MP) Principal Component Analysis (PCA) denoising. MP-PCA is a singular value (SV) decomposition truncation approach that relies on redundant acquisitions, i.e. such that the number of measurements is large compared to the number of components that are maintained in the truncated SV decomposition. Here we used in vivo and synthetic data to test whether a unified readout enables more efficient MP-PCA denoising of less redundant acquisitions, since these can be denoised jointly with more redundant ones. We demonstrate that a unified readout provides robust multi-parametric maps, including diffusion and kurtosis tensors from diffusion MRI, myelin metrics from two-pool magnetisation transfer, and T1 and T2 from relaxometry. Moreover, we show that MP-PCA improves the quality of our multi-contrast acquisitions, since it reduces the coefficient of variation (i.e. variability) by up to 17% for mean kurtosis, 8% for bound pool fraction (myelin-sensitive), and 13% for T1, while enabling more efficient denoising of modalities limited in redundancy (e.g. relaxometry). In conclusion, multi-parametric spinal cord qMRI with unified readout is feasible and provides robust microstructural metrics with matched resolution and distortions, whose quality benefits from multi-contrast denoising methods such as MP-PCA.

## Highlights

• We present a multi-parametric MRI protocol for in vivo spinal cord microstructural imaging based on a unified signal readout.
• The protocol enables the evaluation of diffusion, relaxation and myelin metrics with matched resolution and distortions.
• The unified readout enables multi-contrast analyses, which are demonstrated by multi-contrast MP-PCA denoising.
• Simulations and multi-vendor in vivo data show that MP-PCA is a useful pre-processing step for spinal cord imaging pipelines.
• The performance of MP-PCA greatly benefits from the increased number of measurements enabled by the unified readout.

## List of symbols and abbreviations

AD: Axial Diffusivity
*b*: Diffusion weighting strength (b-value)
BPF: Bound Pool Fraction
CNR: Contrast-to-noise Ratio
CoV: Coefficient of Variation
CSF: Cerebrospinal Fluid
$\Delta f_{c}$: Off-resonance pulse offset frequency
DW: Diffusion-weighted
EPI: Echo Planar Imaging
FA: Fractional Anisotropy
FFE: Fast-Field-Echo
FOV: Field-of-view
FSL: FMRIB Software Library
g: Diffusion weighting gradient direction
GM: Grey Matter
GREASE: Gradient Echo Asymmetric Spin Echo
IQR: Interquartile range
IR: Inversion Recovery
k: Free-to-bound pool exchange rate
MD: Mean Diffusivity
MK: Mean Kurtosis
MP: Marchenko-Pastur
MRI: Magnetic Resonance Imaging
MT: Magnetisation Transfer
mTE, multi-TE: Multi-echo time
PCA: Principal Component Analysis
θ: Off-resonance pulse flip angle
qMRI: Quantitative Magnetic Resonance Imaging
qMT: Quantitative Magnetisation Transfer
ρ: Relative Proton Density
RARE: Rapid Acquisition with Refocussed Echoes
RD: Radial Diffusivity
rFOV: Reduced Field-of-view
SCT: Spinal Cord Toolbox
SNR: Signal-to-noise Ratio
SPGR: Spoiled Gradient Echo
SV: Singular Value
$\;T_{1}$: Macroscopic longitudinal relaxation time
$\;T_{2}$: Macroscopic transverse relaxation time
$\;T_{2}^{B}$: Bound-pool transverse relaxation time
$\;T_{2}^{F}$: Free-pool transverse relaxation time
TE, $\;T_{E}$: Echo time
TEDDI: TE-Dependent Diffusion Imaging
TI, $\;T_{I}$: Inversion time
TR, $\;T_{R}$: Repetition time
WM: White Matter
ZOOM: Zonally-magnified Oblique Multi-slice

## 1 Introduction

The spinal cord is a small but functionally important structure of the human central nervous system, affected in several common disorders. These are often associated with high disability (Hendrix et al., 2015), and include: multiple sclerosis (Ciccarelli et al., 2019), amyotrophic lateral sclerosis (van Es et al., 2017), spinal cord injury (Ahuja et al., 2017) and many others (Lorenzi et al., 2020). Routine anatomical magnetic resonance imaging (MRI) plays an important role in the diagnosis and management of these conditions (Kearney et al., 2015). However, it only offers macroscopic descriptors of tissue damage that lack specificity for pathophysiology, have limited prognostic value and fail to guide treatment and rehabilitation personalisation (Cohen-Adad, 2018; Stroman et al., 2014; Wheeler-Kingshott et al., 2014). The gradual adoption of quantitative MRI (qMRI) techniques may help overcome the limitations of conventional anatomical MRI. Based on either well-validated biophysical models or parsimonious signal representations (Novikov et al., 2018), qMRI promises to provide estimates of biologically meaningful characteristics, which would make parametric maps vendor-independent (Cercignani and Bouyagoub, 2018). The latest multimodal qMRI techniques exploit the complementary information from different contrasts (De Santis et al., 2016; Stikov et al., 2015), for example relaxometry and diffusion, to better quantify the parameters of tissue microstructure (Benjamini and Basser, 2019; Hutter et al., 2018; Kim et al., 2017; Lemberskiy et al., 2018; Ning et al., 2019; Slator et al., 2019; Veraart et al., 2018).

In vivo qMRI of the spinal cord is increasingly popular (Battiston et al., 2018a, 2018b; By et al., 2017; By et al., 2018; Duval et al., 2017; Grussu et al., 2015, 2019; Ljungberg et al., 2017; Massire et al., 2016; Schilling et al., 2019; Taso et al., 2016) due to recent advancements in scanner hardware (Barry et al., 2018; Duval et al., 2015) and analysis software (De Leener et al., 2017). However, its development is currently hampered by the following two challenges.

Firstly, multi-contrast qMRI in the spinal cord in vivo typically relies on specialised techniques with dedicated signal readout for each contrast (Duval et al., 2017; Massire et al., 2016; Taso et al., 2016). The variety of readouts is not compatible with joint computational modelling of voxel-wise multi-contrast signals, and also limits the alignment of multimodal metrics due to different distortions and susceptibility to physiological noise (Campbell et al., 2018). The second major challenge is related to the fact that data quality in spinal cord imaging remains lower compared to the brain. This is due to the need for high spatial resolution (the spinal cord cross sectional area is about 1 ​cm^2^), which is challenged by artifacts from pulsation (Morozov et al., 2018; Summers et al., 2006) and local magnetic field inhomogeneities (Vannesjo et al., 2018; Verma and Cohen-Adad, 2014). Improving the intrinsic data quality is therefore imperative to facilitate the application of the latest qMRI techniques to the spinal cord, which are still in their infancy as compared to those in the brain (Cohen-Adad, 2018; Wheeler-Kingshott et al., 2014).

In this paper, we propose a unified acquisition for state-of-the-art multimodal qMRI of the spinal cord in vivo that addresses both challenges. Our protocol relies on a unified signal readout based on single-shot spin echo planar imaging (EPI) with reduced field-of-view. (rFOV). Our acquisition provides images whose spatial encoding is identical across a range of MRI contrasts, and thus have the same intrinsic resolution and susceptibility artifacts (i.e. distortions). Importantly, the unified acquisition also enforces the same noise statistics across multiple signal contrasts, thus enabling overall data quality enhancement via denoising of the whole multimodal image set.

Several denoising methods have been proposed in MRI, some of them being promising to enhance the quality of multi-contrast image sets. Examples include total variation (Blomgren and Chan, 1998), non-local (Kafali et al., 2018; Kim et al., 2016; Manjón et al., 2009), wavelet (Scheunders and De Backer, 2007), principal component (Manjón et al., 2015; Sonderer and Chen, 2018) or k-space (Haldar and Liang, 2008; Kim et al., 2016) methods. Here we provide a first demonstration of the usefulness of multi-contrast denoising in in vivo spinal cord settings, as enabled by the unified signal readout. For this practical demonstration, we adopt the MP-PCA technique (Veraart et al., 2016b), since it is a promising, open-access and easy-to-use method that has proven to be useful in a number of MRI contexts (Adanyeguh et al., 2018; Does et al., 2019; McKinnon et al., 2018; Tax et al., 2019). Also, MP-PCA enables the analysis of multi-contrast reconstructed images without the need to obtain raw k-space of complex-valued (Cordero-Grande et al., 2019) data, which is typically not very practical in most real-life clinical settings. Different denoising approaches could have also been tested, and we aim to consider them in future work.

It is important to point out that unified, multi-contrast readouts are well known in MRI literature, given the growing interest in multi-parametric approaches. As compared to such previous approaches (Benjamini and Basser, 2019; Hutter et al., 2018; Kim et al., 2017; Ning et al., 2019; Slator et al., 2019), this paper provides several original contributions. Firstly, multi-contrast MRI approaches have not been demonstrated in the spinal cord in vivo, given the intrinsic challenge to deal with physiological noise, non-rigid motion and distortions. Here we tackle these challenges using a cardiac-gated, reduced field-of-view readout. Secondly, to our knowledge we present the richest in vivo multi-contrast spinal cord protocol. Modality-wise, we focus on diffusion-weighted (DW), quantitative magnetisation transfer (qMT), inversion recovery (IR) and multi echo time (multi-TE) imaging (T1 and T2 mapping). These techniques provide microstructural measurers that are potential biomarkers in neurodegenerative and demyelinating diseases (Battiston et al., 2018a; Kearney et al., 2015). Each of these quantitative methods is potentially useful on its own. However, their joint acquisition enables multi-contrast analyses, where the complementary information of the different indices can be fused to obtain novel metrics such as indices of myelin g-ratio (Cercignani and Bouyagoub, 2018; Duval et al., 2017). Importantly, our work is the first to characterise in detail multi-contrast denoising for application in the spinal cord in vivo against real-life confounders (e.g. signal/noise drifts during the acquisition; non-rigid motion; updates in scanners gains). Therefore, it provides the community with quantitative figures related to image quality and metrics variability that are useful for the design of sample sizes in future clinical studies.

## 2 Background on MP-PCA

MP-PCA denoising (Veraart et al., 2016b) is a singular value (SV) decomposition truncation method. MP-PCA denoises noisy input matrices $A=\left[a_{i,j}\right]$ of size M×N constructed by arranging M MRI measurements along rows from N neighbouring voxels along columns, such that M<N without loss of generality.

MP-PCA studies the squared SVs of A, which we indicate as $\left\{\lambda_{1},\;\ldots ,\;\lambda_{M}\right\}$ s.t. $\lambda_{k-1}\geq \;\lambda_{k}$ for k=2,…,M, and selects a threshold at the P-th SV $\lambda_{P}$ by identifying an MP distribution on the distribution of the remaining M−P smallest SVs of A, which are fully-random in the limit case of infinitely large matrices (Baik et al., 2005; Hoyle and Rattray, 2004). Once the threshold $\lambda_{P}$ has been identified, MP-PCA sets the M−P SVs $\lambda_{k}$ (k>P) to zero and outputs a matrix $A^{\text{den}}=\left[a_{i,j}^{\text{den}}\right]$**,** i.e. a denoised version of A, as well as the simultaneously estimated noise standard deviation σ and the number of SVs P that survive truncation. As far as the output matrix $A^{\text{den}}$ is concerned, it is important to keep in mind that not all original noise-free signal components might be recovered, since some unrecoverable signal may be represented in the M−P nullified components.

It should be noted that the P remaining SVs are corrupted with thermal noise, such that the signal-to-noise ratio (SNR) gain can be roughly estimated as $\sqrt{M/P}$ (Veraart et al., 2016a, 2016b). This motivates the application of MP-PCA on large *redundant* image series, i.e. such that they are characterised by M≫P. This fuels the hypothesis that bundling contrasts together to increase M may improve the performance of MP-PCA, thus motivating our work.

Importantly, several other methods for low-rank matrix denoising have been proposed in the literature (Bunea et al., 2011; Giraud, 2011; Kargin, 2015; Nadakuditi, 2014), including statistical/information theory (Johnstone, 2006) approaches deriving optimal asymptotic matrix denoisers (Donoho et al., 2018; Gavish and Donoho, 2014, 2017; Shabalin and Nobel, 2013). Notably, the *optimal shrinkage* approach (Gavish and Donoho, 2017) not only sets to zero SV below the optimal SV threshold $\lambda_{P}$, but also reduces the values of $\lambda_{k}$ for k≤P, improving denoising performance compared to hard SV truncation. Here we used MP-PCA to provide a practical demonstration of multi-contrast applications unlocked by our unified readout. MP-PCA is an open-source tool developed in the imaging context that is well-known and widely adopted within the MRI community, having shown utility and robustness in real-life scenarios (e.g. in presence of signal, noise or frequency drifts as well as of physiological noise) (Adanyeguh et al., 2018; Does et al., 2019; McKinnon et al., 2018; Tax et al., 2019). Other multi-contrast denoising approaches could also show benefit, and we reserve them for future investigation.

## 3 Methods

We synthesised multimodal MRI scans encompassing modalities with different redundancy, emphasising protocols that could be realistically implemented in the spinal cord in vivo, and evaluated the performance of MP-PCA denoising when performed on each modality independently or on multiple modalities jointly.

We also acquired multi-contrast MRI data with unified readout on scanners from two vendors (vendor 1: Philips; vendor 2: Siemens), and characterised the quality of several qMRI metrics as obtained following MP-PCA denoising or without denoising. The shared readout enables the assessment of whether denoising modalities characterised by limited redundancy can be improved if these are denoised jointly with more redundant acquisitions.

In the following sections, we will describe simulations first, as these provide the context for the interpretation of findings in vivo. All analyses were performed using in-house scripts, which are made openly available (http://github.com/fragrussu/PaperScripts/tree/master/sc_unireadout).

### 3.1 In silico study

#### 3.1.1 Signal synthesis

We synthesised realistic spinal cord scans using anatomical information from the Spinal Cord Toolbox.

(http://github.com/neuropoly/spinalcordtoolbox) (SCT) (De Leener et al., 2017), which contains a high-resolution MRI template with voxel-wise volume fractions of white matter (WM, $v_{\text{WM}}$), grey matter (GM, $v_{\text{GM}}$) and cerebrospinal fluid (CSF, $v_{\text{CSF}}$) (Lévy et al., 2015).

Firstly, we used NiftyReg (http://niftyreg.sf.net) **reg_resample** (Modat et al., 2010) with default options to downsample the voxel-wise volume fractions $v_{\text{WM}}$, $v_{\text{GM}}$ and $v_{\text{CSF}}$ to a resolution that is plausible for quantitative MRI of the spinal cord based on EPI (By et al., 2018; Duval et al., 2015; Grussu et al., 2015), i.e. 1 ​× ​1 ​× ​5 ​mm^3^ along R-L, A-P and S–I directions, ensuring realistic partial volume effects. Afterwards, we cropped the field-of-view (FOV) along the foot-head direction to 200 ​mm (40 slices), in order to keep a tractable number of synthetic spinal cord voxels to analyse (i.e. 1700 voxels).

We used custom-written Matlab (The MathWorks, Inc., Natick, MA) code to synthesise signals for a rich multimodal quantitative MRI protocol encompassing of DW, qMT, IR and multi-TE imaging with shared imaging readout (protocol in Table 1, matching our rich in vivo MRI protocol). The total voxel-wise noise-free magnitude signal $S_{\text{TOT}}$ was obtained as the weighted sum of the signals from WM, GM and CSF, i.e.

(1) $$S_{\text{TOT}}=\text{ ​}v_{\text{WM}}\;S_{\text{WM}}\;+\;\;v_{\text{GM}}\;S_{\text{GM}}\;+\;\;v_{\text{CSF}}\;S_{\text{CSF}},$$

where $v_{\text{WM}}+\;v_{\text{GM}}+\;v_{\text{CSF}}=1$.

**Table 1:** Sequence parameters used to simulate synthetic multimodal spinal cord scans. In the table, DW, qMT, IR and multi-TE stand respectively for diffusion-weighted, quantitative magnetisation transfer, inversion recovery and multi-echo time. All of DW, qMT, IR and multi-TE imaging rely on the same spin echo EPI readout with long TR (i.e. such that it is hypothesised that TR ​≫ ​T1). For qMT, each of the 4 repetitions of 11 ​MT-weighted measurements is characterised by a different delay between the end of the off-resonance pulse train and the readout, i.e. {17, 95, 173, 251} ms. The off-resonance pulse train in qMT was made of 25 sinc-Gaussian pulses (bandwidth: 122 ​Hz), each lasting 15 ​ms and with inter-pulse delay of 15 ​ms (Battiston et al., 2018a).

**Scan**	Echo time TE [ms]	Inversion time TI [ms]	Diffusion encoding strength *b* [s/mm^2^]	Off-resonance pulse flip angleθ [°]	Off-resonance pulse offset frequency$\Delta f_{c}$ [KHz]
**DW imaging**	72	No inversion pulse used	{0, 300, 1000, 2000, 2800} s/mm^2^ with {8, 4, 10, 18, 28} directions	No off-resonance pulse used	No off-resonance pulse used
**qMT** **imaging**	24	No inversion pulse used	No diffusion encoding used	4 repetitions of {0, 426, 433, 524, 1429, 1438, 1440, 1459, 1460, 1462, 1465}	4 repetitions of {0.00, 1.07, 1.00, 2.70, 14.13, 3.78, 13.60, 1.05, 1.01, 3.76, 8.39}
**IR imaging**	24	12 linearly spaced in [200, 2300] ms	No diffusion encoding used	No off-resonance pulse used	No off-resonance pulse used
**multi-TE** **imaging**	{25, 40, 55, 70, 85, 100, 200}	No inversion pulse used	No diffusion encoding used	No off-resonance pulse used	No off-resonance pulse used

For each measurement characterised by sequence parameters $({\text{T}}_{\text{E}}$, ${\text{T}}_{\text{I}}$, b, g, θ, $\text{Δ}f_{c})$ (respectively: echo time, inversion time, diffusion-weighting strength or b-value, diffusion gradient direction, off-resonance pulse flip angle, off-resonance pulse offset frequency), we synthesised each of $S_{\text{WM}}$, $S_{\text{GM}}$ and $S_{\text{CSF}}$ as:

(2) $$S=\rho e^{-\frac{{\text{T}}_{\text{E}}}{{\text{T}}_{2}}}\left|1-2e^{-\frac{{\text{T}}_{\text{I}}}{{\text{T}}_{1}}}\right|e^{-bg^{\text{T}}\left(\left(\text{AD-RD}\right){\mathrm{zz}}^{\text{T}}+\text{RD}I\right)g\;}w(\theta ,\text{Δ}f_{c};{\text{T}}_{1}{\text{,T}}_{2}^{\text{F}},k,{\text{T}}_{2}^{\text{B}},\text{BPF}).\;$$

Above, I is the 3 ​× ​3 identity matrix, w describes MT-weighting, $z={\left[0\;0\;1\right]}^{\text{T}}$ is aligned with the cord longitudinal axis and (ρ, ${\text{T}}_{1}$, ${\text{T}}_{2}$, AD, RD, k, ${\text{T}}_{2}^{\text{F}}$, ${\text{T}}_{2}^{\text{B}}$, BPF) are tissues-specific parameters, in this order: relative proton density (Mezer et al., 2013), macroscopic longitudinal and transverse relaxation rate (Smith et al., 2008), axial and radial diffusivity (Basser et al., 1994), free-to-bound pool exchange rate, free pool transverse relaxation rate, bound pool transverse relaxation rate, bound pool fraction (Henkelman et al., 1993). Eq. (2) models water relaxation as mono-exponential; diffusion as Gaussian, described by an axially symmetric diffusion tensor with primary diffusion direction aligned with the cord longitudinal axis; exchange between free and bound (i.e. myelin) protons according to the two-pool MT model (Henkelman et al., 1993). The MT-weighting factor w was calculated via direct numerical integration of the two-pool Bloch equations (details in Supplementary Material S1), assuming a super-Lorentzian line shape for bound protons and simulating off-resonance pulse trains made of 25 sinc-Gaussian pulses (bandwidth: 122 ​Hz), each lasting 15 ​ms and with inter-pulse delay of 15 ​ms, as used before in spinal cord applications (Battiston et al., 2018a).

We synthesised a unique noise-free signal profile in each tissue voxel by simulating within-tissue variability in WM and GM. This ensures that each synthetic voxel has its own unique sources of signal, avoiding obvious redundancies within the set of synthetic signals, as these could lead to overestimation of the performances of MP-PCA denoising (Ades-Aron et al., 2018). In practice, we drew voxel-wise values for each of (ρ, ${\text{T}}_{1}$, ${\text{T}}_{2}$, AD, RD, k${\text{ ​T}}_{2}^{\text{F}}$, ${\text{T}}_{2}^{\text{B}}$, BPF) from a tissue-specific Gaussian distribution, with parameters inspired by values known from literature (Battiston et al., 2018a; Grussu et al., 2015; Smith et al., 2008) (parameters in Table 2).

**Table 2:** Tissue parameters used to generate the synthetic spinal cord scans. Values are inspired by previous literature (Battiston et al., 2018a; Grussu et al., 2015; Smith et al., 2008). For white/grey matter, within-tissue variability was simulated by drawing parameter values from a Gaussian distribution and assigning the obtained values to different voxels. The mean and standard deviation of the Gaussian distributions are reported in the table (standard deviation within brackets, equal to 10% of the mean). For the cerebrospinal fluid (CSF), tissue parameters were fixed to the same values across all CSF-containing voxels.

**Tissue**	Relative proton density *ρ*	Longitudinal relaxation time T_1_ [ms]	Transverse relaxation time T_2_ [ms]	Axial diffusivity AD [μm^2^/ms]	Radial diffusivity RD [μm^2^/ms]	Free-to-bound proton exchange rate *k* [1/s]	Free proton transverse relaxation time T_2_^F^ [ms]	Bound proton transverse relaxation time T_2_^B^ [μs]	Bound pool fraction BPF
**White matter**	0.70 (0.07)	1000 (100)	70 (7)	2.10 (0.21)	0.40 (0.040)	2.3 (0.23)	We fix T_2_^**F**^ ​= ​T_2_	12 (0.12)	0.14 (0.014)
**Grey matter**	0.80 (0.08)	1200 (120)	80 (8)	1.60 (0.16)	0.55 (0.055)	1.7 (0.17)	We fix T_2_^**F**^ ​= ​T_2_	12 (0.12)	0.08 (0.008)
**CSF**	1.00	4000	800	3.00	3.00	Not defined (BPF ​= ​0)	We fix T_2_^**F**^ ​= ​T_2_	Not defined (BPF ​= ​0)	0.00

The synthetic spinal cord phantom is made openly available online (http://github.com/fragrussu/PaperScripts/tree/master/sc_unireadout/sc_phantom).

#### 3.1.2 Denoising

We corrupted the synthetic signals with Gaussian and Rician noise at different SNRs (300 unique noise realisations on 1700 voxels), ranging from 10 to 40 (SNR evaluated with respect to the b=0 signal in WM for the DW measurements).

Afterwards, we used the Matlab implementation of the MP-PCA algorithm (http://github.com/NYU-DiffusionMRI/mppca_denoise) to denoise the synthetic spinal cord images at the various SNRs.

For our simulations, we processed MP-PCA matrices constructed by arranging spinal cord voxels within an individual MRI slice along rows and different MRI measurements along columns (i.e. slice-by-slice cord denoising). We implemented three different denoising strategies:

1. Individual denoising of each modality among DW, IR, multi-TE and quantitative MT imaging respectively (importantly, IR, multi-TE have limited redundancy and would not theoretically qualify for MP-PCA, which is expected to remove little to no noise);
2. Joint denoising of all modalities concatenated as one large set of measurements;
3. Joint denoising of DW imaging concatenated with each of IR, multi-TE and qMT imaging in series respectively, which would be useful to describe cases when only one modality other than DW imaging is acquired.

#### 3.1.3 Analysis

We evaluated the performance of MP-PCA denoising by studying the percentage relative error ε between the denoised signals $S_{\text{TOT},\text{denoised}}$ and the ground truth (noise-free) signal $S_{\text{TOT}}$. We estimated accuracy and precision of the different denoising strategies by calculating respectively the median of ε (such that the closer to zero, the higher the accuracy) and interquartile range (IQR) of ε (such that the lower, the higher the precision) within the synthetic spinal cord over the 100 noise instantiations.

Additionally, we performed the SV decomposition on noisy, noise-free and MP-PCA denoised signal matrices (Matlab function **svd( )**) at various SNRs (both Gaussian and Rician noise) for a representative synthetic MRI slice. This enabled the visualisation of the MP-PCA threshold given the set of matrix SVs.

### 3.2 In vivo study

We performed clinically viable, multi-contrast spinal cord qMRI scans on healthy volunteers and analysed them to characterise the performance of MP-PCA denoising on different qMRI modalities, devising denoising strategies for acquisitions with different levels of redundancy. The experimental sessions were approved by local research ethics committees.

Our qMRI protocols exhibit a unified signal readout, which is based on spin echo EPI, a typical choice for DW imaging. The shared readout ensures comparable noise characteristics across the different qMRI modalities, thus enabling joint denoising of different qMRI contrasts. The MRI protocol in vendor 1 encompasses DW, qMT, IR and multi-TE imaging, while in vendor 2 includes DW and multi-TE imaging. The MRI protocol in vendor 2 is less rich due to practical availability of pulse sequences. Nonetheless, it suffices to demonstrate the potential of joint multi-contrast denoising of modalities with different redundancies, and is representative of protocols required in multi-contrast techniques such as TEDDI (Veraart et al., 2018).

In all systems, MRI scans were performed axially-oblique at the level of the cervical cord, with field-of-view centred at the C2–C3 intervertebral disk (foot-head coverage of 60 ​mm). The whole set of MRI sequence parameters used for both vendors is reported in Supplementary Material S2.

#### 3.2.1 MRI: vendor 1

The protocol developed on a 3T Philips Achieva machine, located at the UCL Queen Square Institute of Neurology (London, UK) consisted of multi-contrast, single-shot spin echo EPI scans with unified signal readout based on reduced FOV (rFOV) ZOOM technology (Wheeler-Kingshott et al., 2002), which enable 4 contrast mechanisms to be exploited: DW imaging, qMT imaging, IR imaging and multi-TE imaging (mTE, i.e. acquisitions of single-shot images at different TE). Salient sequence parameters, including information on b-values, echo/inversion times, off resonance saturation and cardiac gating are reported in Table 3.

**Table 3:** Salient sequence parameters for the qMRI protocol with unified readout implemented on vendor 1 (Philips Achieva, London, UK). DW, qMT, IR and multi-TE stand for diffusion-weighted, quantitative magnetisation transfer, inversion recovery and multi-echo time. Consistently with simulations, in qMT each repetition is characterised by a different delay between the end of the off-resonance train and the readout, i.e. {17, 95, 173, 251} ms. qMT off-resonance trains were made of 25 sinc-Gaussian pulses (bandwidth: 122 ​Hz), each lasting 15 ​ms and with inter-pulse delay of 15 ​ms (Battiston et al., 2018a).

Scan	**TE [ms]**	**TR [ms]**	**RL-AP field-of-view [mm**^**2**^**]**	**Resolution [mm**^**2**^**]**	Slices	**Bandwidth [Hz/pixel]**	Acceleration	Parameters for quantitative imaging	**Nominal scan** time
**DW imaging**	71	12000 (peripheral gating, delay of 150 ​ms)	64 ​× ​48	1 ​× ​1	12 slices, 5 ​mm-thick, 3 packages	2132	Half-scan factor of 0.6	8 b = 0; b = {300, 1000, 2000, 2800}s mm^−2^ with {4, 10, 18, 28} gradient directions	16 ​min: 41 ​s
**qMT** **imaging**	24	7246	64 ​× ​48	1 ​× ​1	12 slices, 5 ​mm-thick, 3 packages	2132	Half-scan factor of 0.6	1 non-MT weighted, 10 ​MT-weighted; 4 repetitions with different slice ordering (same off-resonance pulses as in Table 1)	16 ​min: 18 ​s
**IR imaging**	24	8305	64 ​× ​48	1 ​× ​1	12 slices, 5 ​mm-thick, 3 packages	2132	Half-scan factor of 0.6	12 linearly-spaced inversion times TI in [100; 2300] ms; spacing of 200 ​ms	4 ​min: 50 ​s
**multi-TE** **imaging**	Various	12000 (1 dummy scan per TE)	64 ​× ​48	1 ​× ​1	12 slices, 5 ​mm-thick, 3 packages	2132	Half-scan factor of 0.6	7 echo times TE: {25, 40, 55, 70, 85, 100, 200} ms	2 ​min: 48 ​s

The protocol also included an anatomical 3D FFE scan (flip angle of 7°, TE of 4.1 ​ms, TR of 20 ​ms, resolution of 0.75 ​× ​0.75 ​× ​5 ​mm^3^ and field-of-view of 180 ​× ​240 ​× ​60 ​mm^3^ along R-L, A-P, S–I directions; ProSet fat suppression, 3 signal averages, scan time of 3 ​min: 30 ​s) and standard B0 and B1 field mapping for accurate qMT analysis. Both B0 and B1 mapping were based on 3D FFE acquisitions with resolution of 2.25 ​× ​2.25 ​× ​5 ​mm^3^ and FOV of 215 ​× ​206 ​× ​60 ​mm^3^ along R-L, A-P, S–I directions. B0 mapping was performed with the double-echo method (Jezzard and Balaban, 1995), with parameters: flip angle of 25°, TE of 6.9 ​ms and 9.2 ​ms, TR of 50 ​ms, scan time of 1 ​min: 40 ​s. B1 mapping was instead performed via actual flip angle imaging (Yarnykh, 2007), with parameters: flip angle of 60°, TE of 2.5 ​ms, TR of 30 ​ms, TR extension of 120 ​ms, scan time of 1 ​min: 40 ​s.

For signal reception, the vendor’s 16-channel neurovascular receive-only coil was used. The nominal acquisition time was roughly 47 ​min, with variations depending on subject’s heart rate. We scanned 4 healthy volunteers twice (2 males, age range 28–40), with the rescan performed within one month of the first scan.

#### 3.2.2 MRI: vendor 2

For vendor 2, we performed scans on two separate 3T Siemens Prisma systems, located at the New York University School of Medicine (USA) and at the Neuroimaging Functional Unit of the University of Montreal (Canada).

The protocol consisted in exploiting 2 contrast mechanisms including DW imaging and multi-TE imaging with unified readout based on syngo ZOOMit rFOV technology (Rieseberg et al., 2002) (salient parameters including b-values, TEs and cardiac gating are reported in Table 4). The protocol also included a 3D MEDIC scan for anatomical depiction (flip angle of 30°, TE of 15 ​ms, TR of 625 ​ms, resolution of 0.50 ​× ​0.50 ​× ​5 ​mm^3^ and FOV of 128 ​× ​128 ​× ​60 ​mm^3^ along R-L, A-P, S–I directions; 3 signal averages, scan time of 6 ​min: 24 ​s).

**Table 4:** Salient sequence parameters for the qMRI protocol with unified readout implemented on vendor 2 (Siemens Prisma systems in New York, USA and Montreal, Canada). DW and and multi-TE stand respectively for diffusion-weighted and multi-echo time.

Scan	**TE [ms]**	**TR [ms]**	**RL-AP field-of-view [mm**^**2**^**]**	**Resolution [mm**^**2**^**]**	Slices	**Bandwidth [Hz/pixel]**	Acceleration	Parameters for quantitative imaging	**Nominal scan time**
**DW imaging**	85	9194 (peripheral gating, delay of 200 ​ms)	64 ​× ​64	1 ​× ​1	12 slices, 5 ​mm-thick, 12 concatenations	1240	6/8 Partial Fourier Imaging	4 ​b ​= ​0; b ​= ​{300, 1000, 2000, 2800} s mm^−2^ with {6, 12, 20, 30} directions in NY and {20, 20, 20, 30} directions in Montreal	11 ​min: 02 ​s in NY; 14 ​min: 24 ​s in Montreal (a higher number of DW images acquired in Montreal)
**multi-TE** **imaging**	various	13000 (peripheral gating, delay of 200 ​ms)	64 ​× ​64	1 ​× ​1	12 slices, 5 ​mm-thick, 12 concatenations	1240	6/8 Partial Fourier Imaging	7 echo times TE: {45, 60, 75, 90, 105, 120, 200} ms	1 ​min: 31 ​s

The total scan time was 18 ​min: 57 ​s in the New York Prisma and 22 ​min: 19 ​s in the Montreal Prisma, with the scan time difference due to slightly higher number of diffusion directions being acquired in Montreal. Two subjects were scanned in New York (1 male, 28 years old; 1 female, 25 years old) and one subject (male, 28 years old) in Montreal after obtaining informed written consent. The vendor-provided 64 channel head-neck coil was used in both cases for signal reception.

#### 3.2.3 Denoising

We implemented the same denoising strategies as in simulations:

1. Individual denoising of each modality separately;
2. Joint denoising of all modalities together;
3. Joint denoising of DW imaging concatenated with each of IR, multi-TE and qMT imaging in series (multi-TE only for Prisma).

We performed denoising slice-by-slice to account for the anisotropic voxel-size and to limit the effect of potential between-shot signal fluctuations due to physiological noise (Summers et al., 2006). We proceeded as follows:

• the spinal cord was identified on the mean DW image with SCT **sct_propseg** (De Leener et al., 2014);
• all cord voxels of an MRI slice were arranged as one matrix and denoised with MP-PCA;
• noise floor (Gudbjartsson and Patz, 1995) was subsequently mitigated on the denoised signals with the method of moments (Koay and Basser, 2006). Note that for both vendors we study magnitude images, which exhibit a noise floor. Also, it is well known that scanner reconstruction software may perform certain filtering operations in the background. Therefore, overall the noise distribution in vivo is not expected to be Gaussian.

We estimated voxel-wise SNR in vivo and for both vendors by taking as reference the mean non-DW image (mean *b* ​= ​0 image). For this purpose, we normalised the mean non-DW image by the MP-PCA estimate of noise standard deviation obtained by denoising the DW scan alone. Afterwards, we calculated the mean SNR within the spinal cord for each subject and scan.

#### 3.2.4 Post-processing

We performed motion correction on the concatenation of all acquired EPI images within an MRI session. Practically, we ran slice-wise rigid motion correction with **sct_dmri_moco** on the non-denoised scans, treating qMT, IR and multi-TE images as *b* ​= ​0 scans. The estimated registration transformations were stored and used to correct all the denoised versions of each qMRI modality, as well as the non-denoised data. This was done to focus our analysis on the effect that thermal noise removal has on qMRI metrics.

Afterwards, we segmented the whole cord and the grey matter in the anatomical spinal cord scan respectively with **sct_propseg** and with **sct_deepseg_gm**. We also segmented the spinal cord in the mean DW EPI image with **sct_propseg**.

Lastly, we co-registered the anatomical spinal cord scan to the mean EPI image with **sct_register_multimodal**, using dilated spinal cord masks in the two image spaces to guide registration (dilation performed with NifTK **seg_maths**, available at http://github.com/NifTK/NifTK). The estimated warping field transformation was used to warp the grey matter mask to EPI space, which was subsequently used to obtain a white matter mask by subtracting it from the whole-cord mask. For vendor 1, the warping field was also used to resample the B0 and B1 magnetic field maps to the EPI space for downstream model fitting.

#### 3.2.5 Evaluation of quantitative metrics

We fit quantitative models/signal representations for the different contrasts and obtain popular metrics that are promising imaging biomarkers. These were:

• diffusion kurtosis imaging (Jensen et al., 2005; Veraart et al., 2011) on DW data (both vendors) with DiPy **dipy.reconst.dkimodule** (http://dipy.org), obtaining voxel-wise diffusion and kurtosis tensors, of which fractional anisotropy (FA), mean diffusivity (MD) and mean kurtosis (MK) were considered for downstream analyses;
• mono-exponential ${\text{T}}_{2}$ relaxation on multi-TE data (both vendors) with MyRelax **getT2T2star.py** (http://github.com/fragrussu/myrelax), obtaining voxel-wise macroscopic ${\text{T}}_{2}$;
• mono-exponential ${\text{T}}_{1}$ relaxation on IR data (vendor 1 only), with MyRelax **getT1IR.py**, obtaining voxel-wise macroscopic ${\text{T}}_{1}$;
• two-pool qMT model on qMT data (vendor 1 only) with custom-written Matlab code (Battiston et al., 2018a), obtaining voxel-wise bound pool fraction BPF, exchange rate k and bound pool transverse relaxation ${\text{T}}_{2}^{\text{B}}$, of which BPF and k were considered for downstream analyses. For qMT fitting, static/transmitted fields were corrected on a voxel-by-voxel basis using the B0 and B1 field maps warped to EPI space.

#### 3.2.6 Analysis

We characterised values of all qMRI metrics by calculating the median within grey and white matter for all denoising strategies (including no denoising).

Furthermore, we quantified each metric variability by calculating a percentage coefficient of variation (CoV) within grey matter and within white matter for all denoising strategies (including no denoising). We defined CoV as

(3) $$\text{CoV ​}=\;100\%\;\times \;\;\frac{IQR}{median}\;,$$

where *IQR* is the interquartile range of a metric within grey/white matter, measuring the metric variability, while median is the median value of the metric within the same tissue. We hypothesise that effective denoising would reduce noise-induced metric variability, resulting in lower IQR and unchanged *median* and hence lower CoV, under assumption that variability due to noise is much larger than the biological variability (please see Fig. 4 of (Ades-Aron et al., 2018)). We also provide estimates of the intrinsic scan-rescan variability of each metric m in both WM and GM as

(4) $$\text{variability}=\;100\%\;\times \;\;\frac{IQR\left(m_{1}-\;m_{2}\right)}{median\left(\frac{\;m_{1}+\;m_{2}\;}{2}\right)}\;\;,$$

where $m_{1}$ and $m_{2}$ are the voxel-wise values of the metric at first scan and rescan. For the evaluation of Eq. (4), we warped parametric maps at rescan to the first scan by estimating and affine transformation with NiftyReg **reg_aladin** on the mean DW image.

Finally, we also evaluated the sharpness of the WM/GM contrast-to-noise ratio (CNR) for all metrics, all denoising strategies, all subjects and scans as

(5) $$\text{CNR}=\;\frac{\left|\;median\left(m_{\text{WM}}\right)-\;\;median\left(m_{\text{GM}}\right)\right|}{\sqrt{\;{\left(IQR\left(m_{\text{WM}}\right)/1.349\right)}^{2}+\;{\left(IQR\left(m_{\text{GM}}\right)/1.349\right)}^{2}\;\;}}\;,$$

where $m_{\text{WM}}$ and $m_{\text{GM}}$ respectively represent voxel-wise values of the generic parametric map m in WM and GM.

## 4 Results

### 4.1 In silico study

**Fig. 1:**
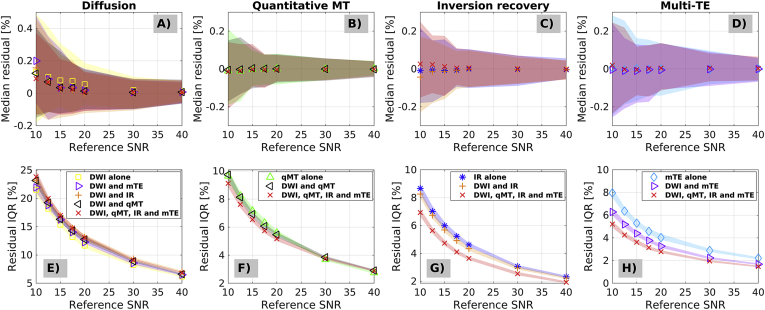
Accuracy and precision of different denoising strategies as obtained from percentage relative errors (percentage errors between denoised signals and noise-free ground truth signals) in simulations. Panels A to D (top) show median percentage at different SNR levels, and represent a measure of accuracy (the closer to zero, the higher the accuracy; DW imaging in A, qMT imaging in B, IR imaging in C, mTE imaging in D). Panels E to H (bottom) show percentage relative error interquartile ranges at different SNR levels, and represent a measure of precision (the closer to zero, the higher the precision; DW imaging in E, qMT imaging in F, IR imaging in G, mTE imaging in H). The SNR is evaluated with respected to the white matter signal on the synthetic DW scan at b ​= ​0.

Fig. 1 shows percentage relative error accuracy (top row: error median) and precision (bottom row: error IQR) of the denoised signals compared to the noise-free ground truth, for different qMRI modalities and different denoising strategies (Gaussian noise). While plots do not highlight any noticeable differences in terms of accuracy for the different denoising strategies (i.e. joint denoising or individual denoising, since their confidence intervals overlap perfectly), they do suggest that better precision (i.e. error IQR closer to zero) can be achieved for modalities that are intrinsically limited in redundancy, when these are denoised jointly with more redundant modalities. For example, IQR drops from 8% to 5% at SNR ​= ​10 for mTE when it is denoised jointly with all modalities, as compared to when mTE is denoised alone. No appreciable improvement of denoising performance is observed with joint multi-contrast denoising for modalities that intrinsically feature high redundancy. This is apparent for qMT and even more so for DW imaging, since their percentage error IQR does not change when these are denoised jointly with other modalites.

**Fig. 2:**
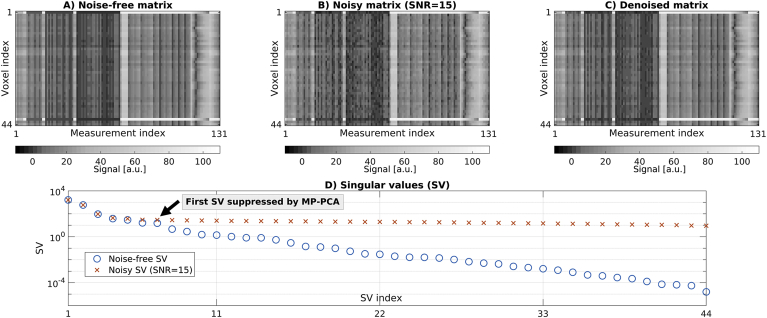
Top: examples of noise-free (A), noisy (B) and denoised (C) matrices from the synthetic spinal cord phantom. Bottom (D): SV decomposition of the noise-free and noisy matrices shown in A and B, alongside MP-PCA cut off (i.e. edge of noisy SV MP distribution). MP-PCA nullifies all SVs starting from the cut off to the right, while it preserves those to the left. The figure reports results from the simulation conducted with Gaussian noise at an SNR of 15, and considers joint denoising of the whole set of 131 MRI measurements from one spinal cord slice made of 44 voxels (concatenation of DW, qMT, IR and mTE imaging).

Fig. 2 shows an illustrative example of noise-free, noisy and denoised signal matrices (Gaussian noise case, simulated SNR of 15, evaluated in white matter for the synthetic diffusion scan at *b* ​= ​0). Note that, strictly speaking, such set of measurements is not redundant, since the noise-free SV spectrum is continuous and none of noise-free SVs are exactly zero. However, the SVs widely vary in their value, over 8 orders of magnitude, such that only a few of them are quite large and dominate the signal. The figure demonstrates that the SV spectrum gets rearranged in the presence of noise in such a way that the contrast exhibited by the smallest SVs is flattened and buried in noise. MP-PCA preserves a few significant SVs that are above the noise floor, while nullifying the rest. The figure focuses on multi-contrast denoising enabled by the unified readout, as this is the main element of novelty of this work. Supplementary Material S3 shows results from all other denoising strategies (including single-contrast denoising), which are in line with Fig. 2.

Supplementary Material S4 shows results from simulations conducted with Rician noise. Results are generally in line with the case considering Gaussian noise. However, residual noise floor biases are apparent at the lowest SNR levels. Supplementary material S5 shows examples of distributions of normalised residuals (i.e. difference between input and output of denoising algorithm).

### 4.2 In vivo study

The SNR in vivo on the *b* ​= ​0 images is estimated to be (mean ​± ​standard deviation across subjects and scans): 21.7 ​± ​2.7 for vendor 1; 17.1 ​± ​2.4 for vendor 2.

**Fig. 3:**
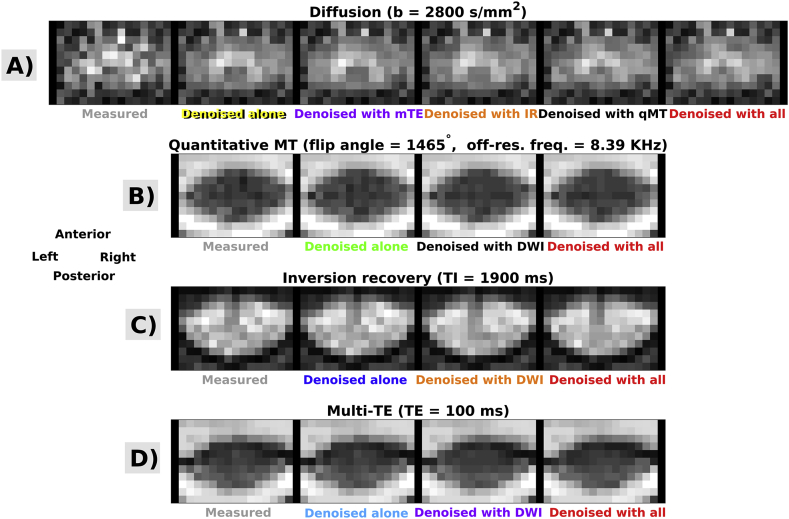
Examples of MP-PCA denoising in one subjects who was scanned with vendor 1. Panels A, B, C, D show raw and denoised images, obtained according to different strategies. DW imaging: panel A; qMT imaging: panel B; IR imaging: panelC; mTE imaging: panelD. *Anterior*, *Posterior*, *Right*, *Left* respectively indicate subject’s anterior, posterior parts and right and left sides.

**Fig. 4:**
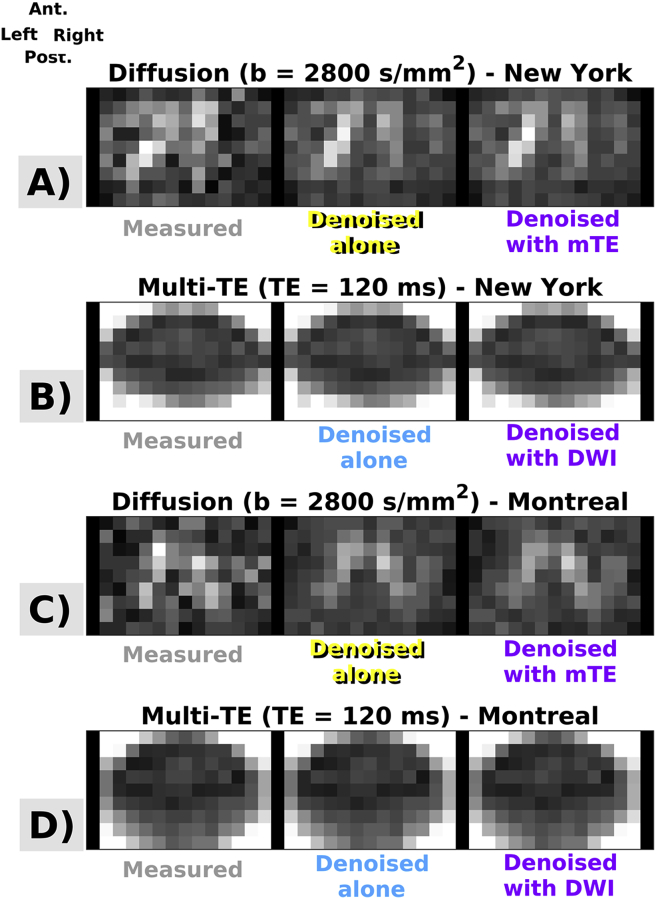
Examples of MP-PCA denoising in two subjects, scanned with vendor 2 respectively in New York and in Montreal. Panels A, B, C, D show raw and denoised images, obtained according to different strategies. DW imaging: images in panels A and C; mTE imaging: images in panels B and D. *Ant.*, *Post.*, *Right*, *Left* respectively indicate subject’s anterior, posterior parts and right and left sides.

Fig. 3, Fig. 4 show examples of acquired and denoised in vivo images. Fig. 3 illustrates information for vendor 1, while Fig. 4 for vendor 2. For both vendors, improvements in image quality are visually and observed, especially for DW imaging. Residual distributions from both simulations and in vivo data exhibit a Gaussian behaviour on visual inspection and are reported in Supplementary Material S5 for illustrative purpose (normally-distributed, spatially-uncorrelated residuals with standard deviation comparable to the noise level are a necessary condition for good denoising (Ades-Aron et al., 2018; Veraart et al., 2016b), although not sufficient).

**Fig. 5:**
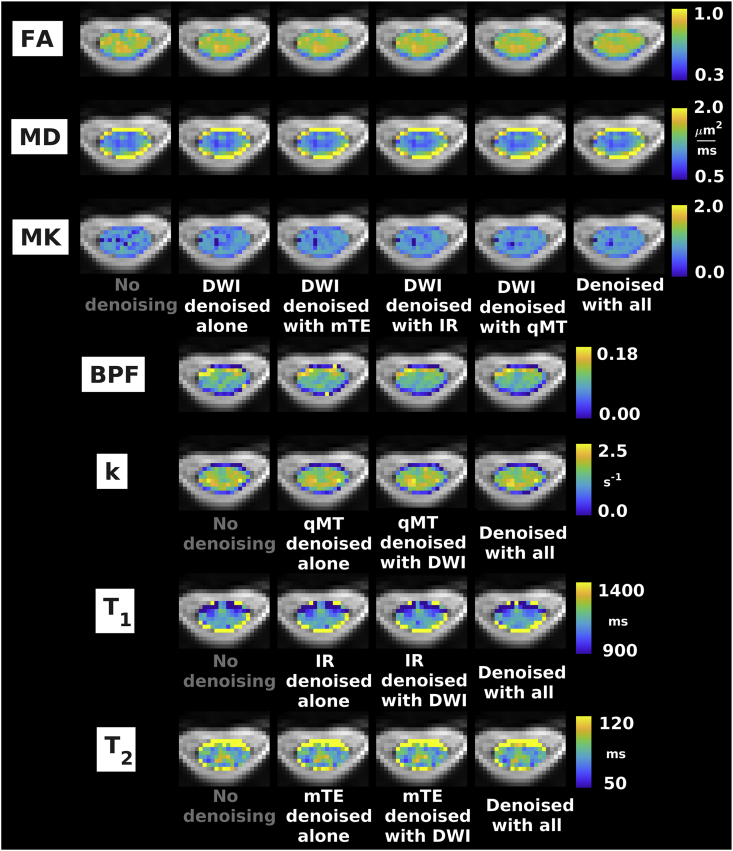
Examples of quantitative maps from vendor 1. From top to bottom: FA, MD, MK (DW imaging); BPF, k (qMT imaging); T1 (IR imaging); T2 (mTE imaging). Different rows illustrate the metrics obtained according to different denoising strategies (no denoising; independent denoising of each modality; various combinations of joint multi-modal denoising). Quantitative maps are overlaid onto the mean non-DW image and shown within the cord only. The same anatomical conventions with regard to subject’s anterior, posterior parts and right and left sides as in Fig. 3 are used.

**Fig. 6:**
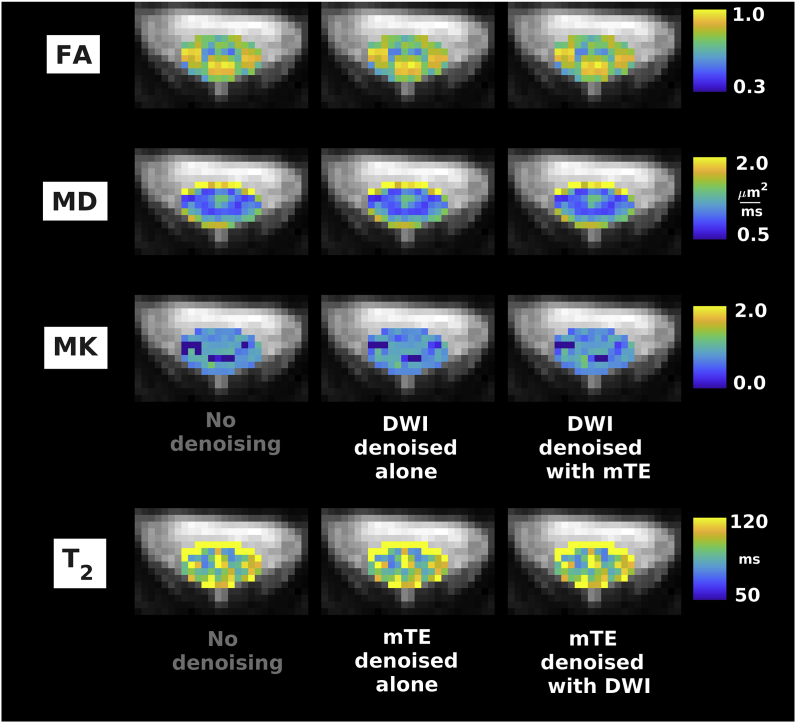
Examples of quantitative maps from vendor 2 (Siemens Prisma system located in New York, USA). From top to bottom: FA, MD, MK (DW imaging); T2 (mTE). Different rows illustrate the metrics obtained according to different denoising strategies (no denoising; independent denoising of each modality; various combinations of joint multi-modal denoising). Quantitative maps are overlaid onto the mean non-DW image and shown within the cord only. The same anatomical conventions with regard to subject’s anterior, posterior parts and right and left sides as in Fig. 4 are used.

**Fig. 7:**
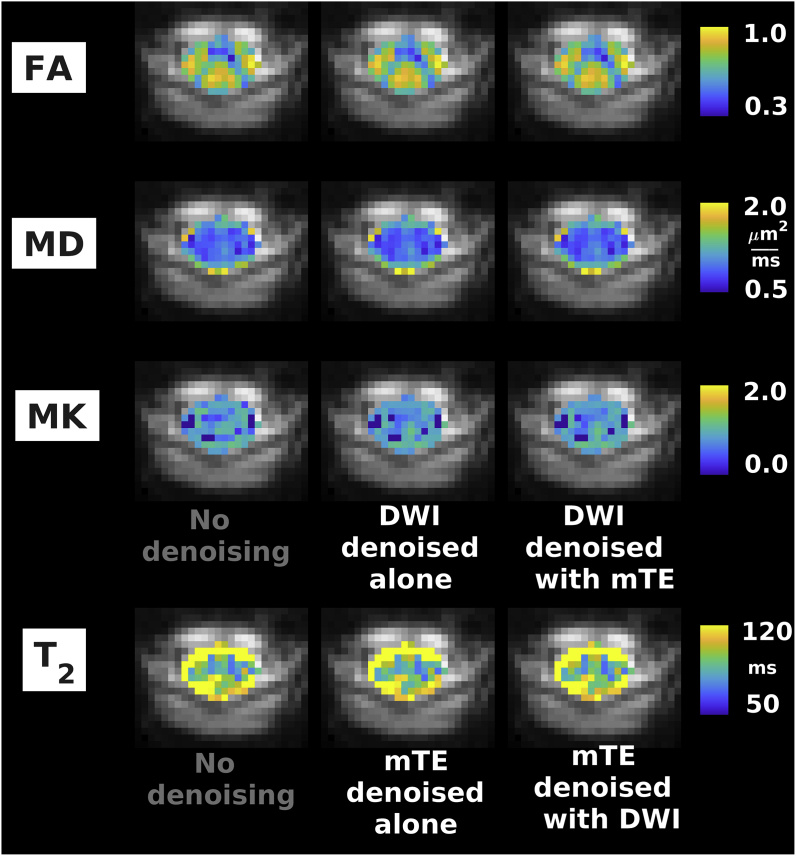
Examples of quantitative maps from vendor 2 (Siemens Prisma system located in Montreal, Canada). From top to bottom: FA, MD, MK (DWI); T2 (mTE). Different rows illustrate the metrics obtained according to different denoising strategies (no denoising; independent denoising of each modality; various combinations of joint multi-modal denoising). Quantitative maps are overlaid onto the mean non-DW image and shown within the cord only. The same anatomical conventions with regard to subject’s anterior, posterior parts and right and left sides as in Fig. 4 are used.

Fig. 5, Fig. 6, Fig. 7 show examples of quantitative parametric maps obtained in one representative subject from vendor 1 (Fig. 5; DW, qMT, IR and mTE imaging) and from the vendor 2 (New York system in Fig. 6, Montreal system in Fig. 7; DW and mTE imaging) for different denoising strategies. Visual inspection suggests that MP-PCA denoising generates less noisy maps, especially for vendor 1. The most striking examples of improved parameter estimation are seen in both vendors for DW imaging parameter MK. Additionally, improvements on visual inspection are apparent for other qMRI metrics such as BPF and ${\text{T}}_{1}$, especially for joint multimodal denoising. Supplementary Material S6 shows voxel-wise differences of all quantitative maps when obtained with and without denoising. No biases in quantitative metrics obtained after denoising are apparent on visual inspection, and the differences between maps obtained with/without denoising appear stronger when multiple contrasts are denoised together.

Table 5, Table 6 report median values of all qMRI metrics in grey and white matter for the different denoising strategies (Table 5: vendor 1; Table 6: vendor 2, pooling together results from the two systems). The tables reveal contrasts between grey and white matter in various metrics. Examples that are consistent between vendors include: higher FA and MD in white compared to grey matter; similar MK in grey/white matter; slightly higher ${\text{T}}_{2}$ in white compared to grey matter. Other examples from vendor 1 include: similar BPF and ${\text{T}}_{1}$ in grey/white matter; higher exchange rate k in grey compared to white matter. The tables also show that systematic differences between the data sets acquired with the two vendors exist, as for example: higher ${\text{T}}_{2}$ and MK and lower MD in data from vendor 2 compared to 1; different grey/white matter contrasts in FA. Notably, Table 5, Table 6 also demonstrate that denoising introduce little to no biases in the quantitative parametric maps. In all cases and for both vendors the tissue-wise medians never differ for more than 5% compared to the values obtained without any denoising.

**Table 5:** Median values in grey and white matter of qMRI metrics obtained with a scanner from vendor 1 following different denoising strategies. Median values from different subjects and scans are pooled so that figures in the table report mean and standard deviation (in brackets) across subjects and scans. In all cases, values of metrics obtained after denoising are less than 5% different from the values obtained with no denoising.

	No denoising	Individual denoising	Joint denoising DWI-mTE	Joint denoising DWI-IR	Joint denoising DWI-qMT	Joint denoising of all
**FA (DWI)**	GM: 0.70 (0.05) WM:0.73 (0.04)	GM: 0.70 (0.06) WM:0.73 (0.04)	GM: 0.70 (0.06) WM:0.74 (0.04)	GM: 0.70 (0.06) WM:0.73 (0.04)	GM: 0.71 (0.06) WM:0.74 (0.04)	GM: 0.71 (0.06) WM:0.73 (0.04)
**MD [μm**^**2**^**/ms] (DWI)**	GM: 1.01 (0.08) WM:1.24 (0.09)	GM: 1.02 (0.08) WM:1.24 (0.09)	GM: 1.02 (0.08) WM:1.24 (0.10)	GM: 1.02 (0.08) WM:1.24 (0.10)	GM: 1.03 (0.08) WM:1.24 (0.10)	GM: 1.03 (0.08) WM:1.24 (0.10)
**MK (DWI)**	GM: 0.75 (0.18) WM:0.75 (0.13)	GM: 0.75 (0.18) WM:0.76 (0.11)	GM: 0.75 (0.20) WM:0.77 (0.11)	GM: 0.74 (0.21) WM:0.76 (0.11)	GM: 0.76 (0.19) WM:0.78 (0.11)	GM: 0.76 (0.19) WM:0.77 (0.11)
**BPF (qMT)**	GM: 0.11 (0.01) WM:0.10 (0.01)	GM: 0.11 (0.01) WM:0.10 (0.01)	NA	NA	GM: 0.11 (0.01) WM:0.10 (0.01)	GM: 0.11 (0.01) WM:0.10 (0.01)
**k [1/s] (qMT)**	GM: 1.73 (0.14) WM:1.52 (0.07)	GM: 1.75 (0.13) WM:1.53 (0.07)	NA	NA	GM: 1.75 (0.14) WM:1.53 (0.08)	GM: 1.75 (0.13) WM:1.54 (0.08)
**T1 [ms] (IR)**	GM: 1108 (22) WM:1131 (5)	GM: 1111 (22) WM:1131 (5)	NA	GM: 1110 (22) WM:1130 (5)	NA	GM: 1108 (21) WM:1130 (6)
**T2 [ms] (mTE)**	GM: 83.8 (5.9) WM: 94.8 (2.9)	GM: 82.7 (5.6) WM: 93.4 (3.1)	GM: 83.3 (5.8) WM: 94.2 (2.9)	NA	NA	GM: 83.0 (6.2) WM: 93.8 (2.8)

**Table 6:** Median values in grey and white matter of qMRI metrics obtained with scanners from vendor 2 following different denoising strategies. Median values from different subjects and scans are pooled so that figures in the table report mean and standard deviation (in brackets) of across subjects and scans. In all cases, values of metrics obtained after denoising are less than 5% different from the values obtained with no denoising.

	**No denoising**	**Individual denoising**	**Joint denoising DWI-mTE**
**FA (DWI)**	GM: 0.68 (0.02) WM: 0.78 (0.01)	GM: 0.68 (0.02) WM: 0.78 (0.01)	GM: 0.68 (0.02) WM: 0.78 (0.01)
**MD [μm**^**2**^**/ms] (DWI)**	GM: 0.99 (0.09) WM: 1.07 (0.08)	GM: 0.98 (0.09) WM: 1.07 (0.08)	GM: 0.98 (0.09) WM: 1.07 (0.08)
**MK (DWI)**	GM: 0.83 (0.05) WM: 0.83 (0.06)	GM: 0.81 (0.07) WM: 0.83 (0.06)	GM: 0.82 (0.06) WM: 0.83 (0.07)
**T2 [ms] (mTE)**	GM: 89.1 (2.7) WM: 104.6 (1.2)	GM: 87.8 (2.7) WM: 102.7 (2.0)	GM: 88.4 (2.7) WM: 104.1 (1.9)

Table 7, Table 8 report within-grey and within-white matter CoV for the various qMRI metrics and for different denoising strategies. Table 7 reports figures from vendor 1, while Table 8 from vendor 2 (data from both systems from vendor 2 pooled together). The tables show that MP-PCA denoising leads to reductions of CoV for various metrics of 5% or more compared to the case with no denoising, as for example for FA, MK, BPF and ${\text{T}}_{1}$ for vendor 1 and MK for vendor 2. Some increases of CoV are observed (for example for MD in white matter for vendor 2). For vendor 1, the strongest reductions in CoV are observed for joint multimodal MP-PCA denoising. For comparison, we also report the intrinsic scan-rescan variability of the metrics when no denoising is applied (vendor 1 only), which is in WM/GM: 25.2/25.5% for FA; 43.6/31.5 for MD; 63.8/63.1% for MK; 77.4/54.9% for BPF; 61.3/43.3% for k; 47.4/14.5% for T1; 48.5/30.4% for T2.

**Table 7:** Percentage CoV in grey and white matter for the various qMRI metrics obtained from vendor 1 (London, UK) with different denoising strategies. The table reports CoV ​= ​100 *iqr/median*, where *iqr* and *median* are respectively the interquartile range and the median of a metric within grey/white matter. CoV from different subjects and scans are pooled so that figures report mean and standard deviation (in brackets) of CoV across subjects and scans. Reductions in mean CoV with more than 5% as compared to the case with no denoising are labelled in bold font, with the percentage reduction of CoV reported explicitly (note that no increase of mean CoV greater than 5% is observed).

	No denoising	Individual denoising	Joint denoising DWI-mTE	Joint denoising DWI-IR	Joint denoising DWI-qMT	Joint denoising of all
**FA CoV [%] (DWI)**	GM: 23.9 (3.3) WM: 24.3 (2.8)	23.7 (3.3) 24.2 (3.0)	23.6 (3.6) 23.8 (3.1)	24.4 (4.5) 23.8 (3.1)	23.2 (4.7) 23.2 (2.7)	**22.6(4.6),-5.4%** 23.3 (2.8)
**MD CoV [%] (DWI)**	GM: 31.2 (15.5) WM: 56.7 (3.3)	30.7 (14.4) 58.3 (4.1)	30.7 (13.5) 58.0 (4.2)	30.5 (13.9) 57.8 (4.1)	29.6 (15.0) 57.6 (2.7)	**29.1(15.8),-6.7%** 57.6 (3.4)
**MK CoV [%] (DWI)**	GM: 70.6 (65.5) WM:58.9 (44.0)	**64.3(59.6),-8.9%** **53.4 (39.7),-9.3%**	69.2 (76.6) **51.9(37.9),-11.9%**	71.1 (81.6) **52.7(39.3),-10.5%**	**61.7(73.2),12.6%** **50.7(39.1),-13.9%**	**59.3(70.0),-16.0%** **50.9(39.2),-13.5%**
**BPF CoV[%](qMT)**	GM: 44.5 (7.4) WM:73.4 (12.3)	42.5 (8.7) 72.5 (14.6)	NA	NA	42.3 (8.1) 71.5 (15.7)	**40.9(7.1),-8.1%** 70.9 (14.9)
**k CoV [%] (qMT)**	GM: 38.0 (9.9) WM:75.6 (16.6)	38.8 (11.0) 74.1 (15.6)	NA	NA	37.9 (10.4) 73.5 (15.8)	37.1 (10.0) 73.2 (15.8)
**T1 CoV [%] (IR)**	GM: 11.7 (6.9) WM:41.7 (39.8)	**10.9(5.4),-6.8%** 41.5 (40.1)	NA	**10.9(6.3),-6.8%** 41.7 (40.2)	NA	**10.2(6.6),-12.8%** 39.9 (40.6)
**T2 CoV [%] (mTE)**	GM:27.8 (9.5) WM:44.4 (13.3)	28.2 (10.2) 44.6 (13.4)	27.5 (10.5) 44.7 (13.8)	NA	NA	26.5 (6.8) 43.7 (13.1)

**Table 8:** Percentage CoV in grey and white matter for the various qMRI metrics obtained from vendor 2 (New York, NY, USA and Montreal, Canada) with different denoising strategies. The table reports CoV ​= ​100 *iqr/median*, where *iqr* and *median* are respectively the interquartile range and the median of a metric within grey/white matter. CoV from different subjects and scans are pooled so that figures report mean and standard deviation (in brackets) of CoV across subjects and scans. Improvements of mean CoV greater than 5% compared to the case with no denoising (i.e. lower CoV) are labelled in bold font, with the percentage reduction of CoV reported explicitly (note that no increase of mean CoV greater than 5% is observed).

	**No denoising**	**Individual denoising**	**Joint denoising DWI-mTE**
**FA CoV [%] (DWI)**	GM: 32.6 (1.0) WM: 23.2 (1.4)	33.1 (1.7) 22.8 (2.0)	33.1 (2.8) 22.9 (1.9)
**MD CoV [%] (DWI)**	GM: 29.7 (7.1) WM: 39.7 (4.1)	30.1 (7.3) 41.0 (3.4)	28.9 (4.7) 40.6 (4.9)
**MK CoV [%] (DWI)**	GM: 46.9 (7.8) WM: 52.0 (6.7)	**39.7 (9.2),-15.4%** **44.1 (4.3),-15.2%**	**38.9 (8.1),-17.1%** **44.3 (5.8),-14.8**
**T2 CoV [%] (mTE)**	GM: 26.3 (1.9) WM: 39.4 (8.1)	25.3 (2.5) 40.1 (8.1)	26.5 (0.9) 39.7 (7.9)

Supplementary Material S7 shows results from WM/GM CNR calculations. Denoising increases WM/GM CNR more than 5% for FA, MK, BPF and T2 in vendor 1 and for MK in vendor 2. Additionally, CNR decreases for MD in vendor 1 more than 5% when DW imaging is denoised jointly with qMT or qMT, IR and mTE imaging.

## 5 Discussion

### 5.1 Summary and key findings

This work demonstrates the advantages of multimodal qMRI of the spinal cord in vivo with unified MRI signal readout. The unified readout enables matching resolution and distortions across different contrasts, thereby also facilitating joint analyses and computational modelling of multi-contrast signal. Here we provide a practical demonstration by studying joint multi-contrast denoising, an example of which is given via MP-PCA denoising. The unified readout enables efficient MP-PCA denoising of modalities that feature a limited number of measurements, when these are denoised jointly with modalities whose protocols is much richer.

Our key findings are that a unified readout enables reliable and detailed microstructural characterisation of the human cervical spinal cord in clinical setting, providing metrics of relaxometry and diffusion as well as myelin-sensitive indices with matched resolution and distortions. Moreover, MP-PCA appears as a valid tool to improve the intrinsic quality of unified readout acquisitions, as supported by both in vivo and in silico data. Finally, this approach is feasible on 3T MRI systems from two major vendors.

### 5.2 In silico study

We have designed and run computer simulations to test whether a unified readout offers opportunities for MP-PCA denoising of qMRI modalities that exhibit limited redundancy (a number of measurements comparable to the number of SVs that would not be zeroed by MP-PCA, i.e. M~P), for which effective MP-PCA denoising remains challenging.

Our simulations suggest that a unified readout has indeed the potential of supporting more efficient MP-PCA denoising for modalities limited in redundancy, as for example mono-exponential and multi-exponential (Does et al., 2019) relaxation mapping. Denoising these modalities jointly with more redundant modalities enables more efficient noise mitigation in the former. Interestingly, joint multimodal denoising did not affect the denoising performance on modalities that are already redundant, as for example DW imaging.

Moreover, calculations on simulated data suggest that MP-PCA finds the SV threshold in a zone of the SV spectrum where the between-SV contrast is flattened by the presence of noise. Importantly, those SVs are various orders of magnitude smaller than the SV that survive MP-PCA thresholding for SNR levels that are realistic in the spinal cord in vivo. Therefore, our analysis suggests that the salient characteristics of the MRI contrast are preserved by MP-PCA.

Finally, the improved denoising accuracy and precision for joint multi-contrast denoising are seen when both Rician and Gaussian noise are considered, giving further confidence on the generalisability of our simulation results to in vivo settings (i.e. in presence of strong noise floors).

### 5.3 In vivo study

In this paper we tested multi-parametric qMRI of the spinal cord with unified readout using 3T MRI scanners from two major vendors (Philips and Siemens), and studied to what extent MP-PCA improves the quality of such MRI data.

Our multi-vendor data demonstrate the feasibility of implementing reliable multi-parametric qMRI of the spinal cord with unified readout. A unified readout provides matched resolution and distortions across MRI contrasts, and ensures comparability of signals across a rich set of qMRI measurements. Moreover, it enables the development of unified analysis pipelines, spanning from motion correction, to data denoising and potentially model fitting, paving the way to joint modelling of multi-contrast signals (Kim et al., 2017). Importantly, it may be useful in techniques that combine information from diffusion with relaxation/myelin-sensitive indices, as for example g-ratio MRI (Campbell et al., 2018; Duval et al., 2017; Stikov et al., 2015)), where matched EPI distortions (Irfanoglu et al., 2015) are crucial (Campbell et al., 2018). Here, we demonstrate our approach in the spinal cord in vivo, but preliminary investigation suggest that it may be useful even in the brain (Grussu et al., 2017).

Innovative elements of this work are the use of MP-PCA denoising across various MRI contrasts, and its application in vivo in the human spinal cord. Our analyses demonstrate that MP-PCA effectively mitigates noise in all modalities and for both vendors. Importantly, quantitative analysis of parametric maps suggests that the performance of MP-PCA in enhancing the quality of modalities with limited redundancy (i.e. IR and mTE imaging) can be improved by denoising these modalities jointly with more redundant schemes.

Our joint multimodal denoising relies on the hypothesis of noise homoscedasticity across MRI contrasts. Supplementary material S8 shows that the estimated noise level on modalities other than DWI follows the same trends as those of estimates from DWI in both simulations and in vivo. The supplementary document also demonstrates that estimating the noise level is a very challenging task: noise level estimates are highly variable per se. Moreover, Supplementary material S8 reveals systematic differences between noise standard deviation estimates from DW imaging compared to other modalities, such as qMT. This is likely attributed to the stronger departures from the hypothesis of Gaussian noise underlying MP-PCA in DW imaging, due to lower SNR and stronger noise floor effects (Koay and Basser, 2006), and to the fact that qMT suffers from stronger physiological noise that may resemble thermal noise (qMT is not cardiac gated). Nonetheless, it should be remembered that MP-PCA noise levels estimated on modalities with limited number of measurements (e.g. multi-TE imaging) are not reliable, as the limited number of measurements does not allow the MP distribution to be detected accurately (Veraart et al., 2016b). Importantly, such differences in terms of noise level estimates among modalities introduce little to no bias in downstream quantitative parameter maps, and therefore do not appear to be a concerning issue for practical MP-PCA deployment.

We also investigated the effect of MP-PCA denoising on the quality of popular parametric maps. To this end, we studied median values of metrics within grey/white matter as well as metric variabilities as quantified by a CoV. Our experiments show that MP-PCA introduces little to no biases in any of the metrics, irrespectively of the chosen denoising strategy (joint multimodal denoising vs modality-wise denoising). The difference in median values between metrics obtained with denoising compared to the case with no denoising are ±5% or less. These differences, which are very low, likely reflect the intrinsic susceptibility of the different model fitting routines to noise fluctuations and noise floors, and are therefore expected since noise-floor mitigation (Koay and Basser, 2006) was performed following MP-PCA denoising. Conversely, MP-PCA does decrease metric variability, as it leads to considerable reductions of tissue-wise CoV, as for example for MK (−16% for vendor1, –17% for vendor 2), FA, BPF and ${\text{T}}_{1}$ (–13% for vendor 1). The reduction in variability is the highest for metrics like MK, which carry important information about tissue microstructure, and that are notoriously difficult to estimate (Veraart et al., 2011). It should be noted the using CoV as a metric to evaluate denoising performance has some intrinsic limitations, since CoV can potentially increase simply because of increased blurring. Our supplementary analysis of sharpness based on GM/WM CNR (Supplementary Material S7) suggests that this is unlikely for most MRI metrics considered here (we observe increases of CNR higher than 5% for FA, MK, BPF and T2). However, it is possible that certain MRI metrics such as MD are more prone to blurring as compared to other metrics, given that we observe a reduction of CNR for this index.

Our parametric maps follow known trends and contrasts, with some differences in terms of relaxometry metrics, e.g. low contrast white/grey matter contrast for T1 and T2. This difference may be explained by residual CSF pulsation that corrupts neighbouring white matter signals, and by the fact that literature values for T1 and T2 are typically obtained with different readout strategies compared to the employed single-shot EPI (Smith et al., 2008). Another explanation, especially for vendor 1, may be related to the coarse resolution of the anatomical scan, required to limit scan time, as this was used for grey matter segmentation potentially introducing partial volume effects in the tissue masks. Overall, while grey/white matter contrasts in parametric maps are similar in data from both vendors, systematic differences between metric values (Table 5 vs Table 6) and variability (Table 7 vs Table 8) are seen. Several factors may have contributed to these differences between vendors, namely in: intrinsic SNR; reduced field-of-view technique; resolution of the anatomical scan used for grey/white matter segmentation; parallel imaging/reconstruction technique; qMRI protocol; between-subject biological differences.

In this study, we employed cardiac gating for some of the contrasts, but not for others, due to practical implementation challenges (e.g. DW imaging was cardiac-gated with a relatively long TR; qMT and IR were not as the variable TR introduced by gating would pose issues to model parameters computation). As a consequence, the non-gated contrasts are likely to suffer from stronger physiological noise than gated modalities. This difference is likely to be preserved after denoising, since MP-PCA is limited to detecting signatures in matrix SVs that are specific to thermal, rather than physiological noise (Veraart et al., 2016a, 2016b). Also, none of our in vivo acquisitions were respiratory-gated. Therefore, all contrasts are likely to be affected by respiratory motion in a similar way.

Finally, we point out that we took care to use the same registration transformations to correct for motion in all denoising strategies, estimating motion on the non-denoised data. We followed this motion correction strategy on purpose, as our focus was to study the effects of thermal noise mitigation on parametric maps. It is possible that the benefits of MP-PCA may extend beyond thermal noise mitigation and may also improve post-processing such as motion correction, as shown in other studies (Ades-Aron et al., 2018), which will be the subject of future investigations. Importantly, we performed motion correction after MP-PCA to avoid altering the noise characteristics. This implies that input MP-PCA signal were affected by motion-induced fluctuations. However, such physiological fluctuations are distinct from thermal noise, and therefore may be still present in the MP-PCA output especially when affecting the part of SV spectrum that survives MP-PCA thresholding (Veraart et al., 2016a, 2016b).

### 5.4 Methodological considerations

We acknowledge a number of potential limitations of our approach.

Firstly, our unified protocol was more comprehensive on the system from vendor 1 as compared to vendor 2. This was due to the practical availability of MRI sequences at the time of acquisition of the data.

Secondly, the DW imaging protocol for the vendor 2 differed between the system in New York and the one in Montreal, with the latter being slightly longer. This was due to a choice in the design of the protocol in Montreal, which would enable the inclusion of the scan in other ongoing group studies.

Thirdly, it must be acknowledged that a unified acquisition based on reduced-field-of-view EPI has some intrinsic limitations. For instance, other readouts could be used for in vivo relaxometry of the spinal cord, as for example spin echo, spoiled gradient echo (SPGR) or gradient echo asymmetric spin echo (GREASE) imaging (Duval et al., 2017; Ljungberg et al., 2017; Smith et al., 2008). In particular the use of EPI for T2 mapping results in substantially longer echo times, which practically limits the possibility of measuring signal from short T2 components. Such components may be relevant contributors to the T2/T2∗ contrast, given the high levels of myelin water in the spinal cord (Ljungberg et al., 2017). On the one hand, a unified readout facilitates the joint computational modelling of multi-contrast signals compared to a mixed readout approach, while also matching distortions. On the other hand, these advantages come at the expense of potential reductions in SNR and hence intrinsic quantitative map quality (e.g. T1 and T2 maps in (Smith et al., 2008); note that single-shot EPI has lower SNR compared to other multi-shot readouts). Importantly, it should be noted that the EPI readout for spinal cord imaging is sensitive to the effects of physiological noise (Summers et al., 2006). These can lead to residual edge artifacts at the WM/CSF boundary, where strong partial volume is likely. This is apparent for instance in our T1 and T2 maps (e.g. higher T2 in WM compared to GM), and is more generally a well-known issue in quantitative spinal cord MRI (Duval et al., 2017; Ljungberg et al., 2017).

An additional limitation of our work regards the use of median, CoV and CNR of parametric maps to evaluate the effect of the denoising. Metrics such as CoV have their own limitations, since they could decrease (i.e. point towards reduced metric variability) simply because of increased blurring. However, this is a common approach when analysing in vivo data, where no “noise-free” ground truth is available. When scan time allows, a different approach to validate an image quality enhancement method could be that of acquiring additional, high SNR scans, which could act as bench mark data.

Finally, we highlight that our analysis on MP-PCA is intended to provide a practical demonstration of the potential of multi-contrast analyses unlocked by a unified readout in the spinal cord. Several different denoising strategies could have also been used (Donoho and Gavish, 2014; Donoho et al., 2018; Gavish and Donoho, 2014; Shabalin and Nobel, 2013), and we aim to consider them in future works focussing on comparisons of denoising methods.

## 6 Conclusions

Multi-parametric qMRI of the spinal cord with unified readout (i.e. with matched resolution and distortions) is advantageous and provides robust microstructural metrics sensitive to axonal characteristics, such as the diffusion propagator, relaxometry and myelin. Our unified acquisition paves the way to joint modelling of multi-contrast signals, and offers unique opportunities for image quality enhancement via joint denoising of multiple contrasts. A practical demonstration of this is provided with the MP-PCA technique, which is shown to be a useful pre-processing step in spinal cord MRI analysis pipelines.

## Author contribution statement

FG: Conceptualization; Methodology; Software; Formal analysis; Investigation; Data Curation; Writing - Original Draft; Visualisation; Funding acquisition; Project administration. MB, JV, TS: Conceptualization; Methodology; Software; Investigation; Writing - Review & Editing. JCA, TMS, DCA, DSN, EF, CGWK: Conceptualization; Methodology; Resources, Supervision, Funding acquisition; Writing - Review & Editing.

## Data and code availability statement

The synthetic spinal cord phantom is made freely available online (http://github.com/fragrussu/PaperScripts/tree/master/sc_unireadout/sc_phantom). All scripts written to analyse the data are also made openly available (http://github.com/fragrussu/PaperScripts/tree/master/sc_unireadout). The Python routines for relaxometry and DKI fitting are made freely available online as part of the open-access GitHub repositories MyRelax (http://github.com/fragrussu/MyRelax) and MRItools (http://github.com/fragrussu/MRItools). All relevant third-party dependencies are clearly indicated.

The in vivo data cannot be made openly available online due to privacy issues of clinical data according to GDPR regulations. Researchers interested in accessing the in vivo data from vendor 1 can contact Prof Claudia Gandini Wheeler-Kingshott (c.wheeler-kingshott@ucl.ac.uk). A data sharing agreement enabling academic and research use will be stipulated. Researchers interested in accessing the in vivo data from vendor 2 can contact: Prof Timothy Shepherd (timothy.shepherd@nyulangone.org) for the New York data; Prof Julien Cohen-Adad (jcohen@polymtl.ca) for the Montreal data. A data sharing agreement will be stipulated with either New York University, Polytechnique Montreal or both, enabling academic and research use.

Other third-party code used in this project is freely available online. This includes: MP-PCA denoising, SCT, NiftyReg, NiftK, DiPy and FSL (MP-PCA: http://github.com/NYU-DiffusionMRI/mppca_denoise; SCT: http://github.com/neuropoly/spinalcordtoolbox; NiftyReg: http://cmictig.cs.ucl.ac.uk/wiki/index.php/NiftyReg; NiftyK: http://github.com/NifTK/NifTK; DiPy: http://dipy.org; FSL: http://fsl.fmrib.ox.ac.uk/fsl/fslwiki).

The code for qMT signal synthesis and analysis (two-pool fitting) is not openly available online. Researchers interested in accessing the code can contact Dr Marco Battiston (marco.battiston@ucl.ac.uk), who would release a copy under a license/sharing agreement enabling academic and research use.

## Funding

This project has received funding under the 10.13039/501100007601European Union’s Horizon 2020 research and innovation programme under grant agreement No. 634541 and 666992, and from: 10.13039/501100000266Engineering and Physical Sciences Research Council (EPSRC EP/R006032/1, M020533/1, G007748, I027084, M020533, N018702); Spinal Research (UK), Wings for Life (Austria), Craig H. Neilsen Foundation (USA) for funding the INSPIRED study; UK Multiple Sclerosis Society (grants 892/08 and 77/2017); Department of Health’s National Institute for Health Research Biomedical Research Centres (UK); Canada Research Chair in Quantitative Magnetic Resonance Imaging (950-230815); 10.13039/501100000024Canadian Institutes of Health Research (CIHR FDN-143263); 10.13039/501100000038Natural Sciences and Engineering Research Council of Canada (RGPIN-2019-07244); Canada First Research Excellence Fund (IVADO and TransMedTech); 10.13039/100010571Quebec Bio-Imaging Network (5886, 35450); the 10.13039/100000002National Institutes of Health NIBIB Biomedical Technology Resource Center grant P41 EB017183 (USA); NIH NIBIB grant R01 EB027075 (USA); NIH NINDS grant R01 NS088040 (USA); 10.13039/501100003130Research Foundation - Flanders 12S1615N (Belgium).

## Declaration of competing interests

TS is an employee of Philips UK. EF, JV, DSN and NYU School of Medicine, are co-inventors in the MP-PCA technology related to this research; a patent application has been filed and is pending. EF, JV, DSN, and TMS are shareholders and hold advisory roles at Microstructure Imaging, Inc.

## Appendix A Supplementary data

Online repository with synthetic data and all code Online repository with synthetic data and all code

S1: the two-pool MT model S1: the two-pool MT model

S2: MRI sequence parameters S2: MRI sequence parameters

S3: SVD of synthetic signals corrupted with Gaussian noise S3: SVD of synthetic signals corrupted with Gaussian noise

S4: Simulations considering Rician noise S4: Simulations considering Rician noise

S5: Distribution of residuals S5: Distribution of residuals

S6: Difference maps S6: Difference maps

S7: Contrast-to-noise ratio S7: Contrast-to-noise ratio

S8: Estimated noise levels S8: Estimated noise levels
