# Supplementary material for: Multi-parametric quantitative in vivo spinal cord MRI with unified signal readout and image denoising
Source: Neuroimage. 2020 Aug 15;217:116884. doi: 10.1016/j.neuroimage.2020.116884 (PMC7378937; doi:10.1016/j.neuroimage.2020.116884)
Supplement: S1: the two-pool MT model [file mmc2.pdf]

Supplementary material S1: the two-pool MT model

The two-pool magnetisation transfer (MT) model is used in this paper in both simulations and in vivo analyses. The model describes the evolution of the magnetisation of two exchanging ^{1}H pools, i.e. free protons in bulk water and protons bound to macromolecules (Henkelman et al., 1993), in presence of off-resonance irradiation $b_{1,\mathrm{off}}(t)$ with carrier frequency $f_0 + \Delta f_c$ (with Δf_c being the offset with respect to water resonance frequency f_0), assumed to be played out at times $0 \le t \le t_{\mathrm{off}}$. On-resonance excitation is then assumed to be played out at $t > t_{\mathrm{off}}$, after a generic delay t_{delay}.

Let us indicate with $\mathbf{m}^{\mathrm{F}}(t) \stackrel{\mathrm{def}}{=} \left[m_x^{\mathrm{F}}(t) \quad m_y^{\mathrm{F}}(t) \quad m_z^{\mathrm{F}}(t)\right]^{\mathrm{T}}$ and $\mathbf{m}^{\mathrm{B}}(t) \stackrel{\mathrm{def}}{=} \left[m_x^{\mathrm{B}}(t) \quad m_y^{\mathrm{B}}(t) \quad m_z^{\mathrm{B}}(t)\right]^{\mathrm{T}}$ the free and bound proton magnetisations during the off-resonance irradiation, and with $\mathrm{T}_1^{\mathrm{F}}$ and $\mathrm{T}_1^{\mathrm{B}}$ and with $\mathrm{T}_2^{\mathrm{F}}$ and $\mathrm{T}_2^{\mathrm{B}}$ their respective longitudinal and transverse relaxation times (Portnoy and Stanisz, 2007). Let us also indicate with k the exchange rate between free to bound protons, with BPF the bound pool fraction (fraction of protons belonging to the bound pool) and with γ the proton gyromagnetic ratio ($\frac{\gamma}{2\pi} \cong \ 42.577 \ \frac{\mathrm{MHz}}{\mathrm{T}}$).

The MT-weighting factor w in Eq. 5 (main manuscript) is defined as the value of the free pool longitudinal magnetisation at the end of off-resonance irradiation, i.e.:

$$w = m_z^{\mathrm{F}}(t = t_{\mathrm{off}}). \qquad (S1.1)$$

The value of w in Eq. S1.1 is obtained directly by numerical integration of the two-pool Bloch equations, which are written as:

$$\frac{d}{dt}\begin{bmatrix} m_x^{\mathrm{F}} \\ m_y^{\mathrm{F}} \\ m_z^{\mathrm{F}} \\ m_z^{\mathrm{B}} \end{bmatrix}(t) = \begin{bmatrix} 0 & 0 & 0 & 0 & 0 \\ 0 & -\frac{1}{\mathrm{T}_2^{\mathrm{F}}} & 2\pi\,\Delta f_c & 0 & 0 \\ 0 & -2\pi\,\Delta f_c & -\frac{1}{\mathrm{T}_2^{\mathrm{F}}} & \gamma\, b_{1,\mathrm{off}}(t) & 0 \\ \frac{1}{\mathrm{T}_1^{\mathrm{F}}} & 0 & -\gamma\, b_{1,\mathrm{off}}(t) & -\left(\frac{1}{\mathrm{T}_1^{\mathrm{F}}} + k\right) & k\,\frac{(1-\mathrm{BPF})}{\mathrm{BPF}} \\ \frac{1}{\mathrm{T}_1^{\mathrm{B}}}\frac{\mathrm{BPF}}{(1-\mathrm{BPF})} & 0 & 0 & k & -\left(\frac{1}{\mathrm{T}_1^{\mathrm{B}}} + R_{\mathrm{RF}}^{\mathrm{B}} + k\,\frac{(1-\mathrm{BPF})}{\mathrm{BPF}}\right) \end{bmatrix} \begin{bmatrix} \frac{1}{2} \\ m_x^{\mathrm{F}} \\ m_y^{\mathrm{F}} \\ m_z^{\mathrm{F}} \\ m_z^{\mathrm{B}} \end{bmatrix}(t).$$

$$(S1.2)$$

For the integration of equation S1.2, it is assumed that $\mathbf{m}^{\mathrm{F}}(t=0) \stackrel{\text{def}}{=} [0 \quad 0 \quad (1-\mathrm{BPF})]^{\mathrm{T}}$, $\mathbf{m}^{\mathrm{B}}(t=0) \stackrel{\text{def}}{=} [0 \quad 0 \quad \mathrm{BPF}]^{\mathrm{T}}$, $m_x^{\mathrm{B}}(t = t_{\mathrm{off}}) = m_y^{\mathrm{B}}(t = t_{\mathrm{off}}) \approx 0$ and that the bound pool off-resonance absorption can be modelled based on a super-Lorentzian line shape. Under this assumption, the absorption term $R_{\mathrm{RF}}^{\mathrm{B}}$ is a function of $(t, \Delta f_c, \mathrm{T}_2^{\mathrm{B}})$ and is modelled as (Portnoy and Stanisz, 2007):

$$R_{\mathrm{RF}}^{\mathrm{B}}(t, \Delta f_c, \mathrm{T}_2^{\mathrm{B}}) = \pi\,\gamma^2 b_{1,\mathrm{off}}^2(t) \int_0^{\frac{\pi}{2}} \sqrt{\frac{2}{\pi}}\,\frac{\sin(\varphi)}{|3\,\cos^2(\varphi)-1|}\,\mathrm{T}_2^{\mathrm{B}} \exp\left(-2\left(\frac{2\pi\,\Delta f_c\,\mathrm{T}_2^{\mathrm{B}}}{3\,\cos^2(\varphi)-1}\right)^2\right) d\varphi \quad (S1.3).$$

For the practical integration of Eq. S1.2, it is assumed that $\mathrm{T}_1^{\mathrm{B}} \approx 1\,s$ (Battiston et al., 2018), and that the free pool longitudinal relaxation time (i.e. $\mathrm{T}_1^{\mathrm{F}}$) is linked to the observable T_1 relaxation time as (Portnoy and Stanisz, 2007):

$$\frac{1}{\mathrm{T}_1^{\mathrm{F}}} \approx \frac{1}{\mathrm{T}_1} - \frac{k\left(\frac{1}{\mathrm{T}_1^{\mathrm{B}}} - \frac{1}{\mathrm{T}_1}\right)}{\frac{1}{\mathrm{T}_1^{\mathrm{B}}} - \frac{1}{\mathrm{T}_1} + k\left(\frac{1-\mathrm{BPF}}{\mathrm{BPF}}\right)}\,. \quad (S1.4)$$

References

Battiston, M., Grussu, F., Ianus, A., Schneider, T., Prados, F., Fairney, J., Ourselin, S., Alexander, D.C., Cercignani, M., Gandini Wheeler-Kingshott, C.A., 2018. An optimized framework for quantitative magnetization transfer imaging of the cervical spinal cord in vivo. Magnetic resonance in medicine 79, 2576-2588.

Henkelman, R.M., Huang, X., Xiang, Q.S., Stanisz, G., Swanson, S.D., Bronskill, M.J., 1993. Quantitative interpretation of magnetization transfer. Magnetic resonance in medicine 29, 759-766.

Portnoy, S., Stanisz, G.J., 2007. Modeling pulsed magnetization transfer. Magnetic Resonance in Medicine: An Official Journal of the International Society for Magnetic Resonance in Medicine 58, 144-155.
