# Supplementary material for: Multi-parametric quantitative in vivo spinal cord MRI with unified signal readout and image denoising
Source: Neuroimage. 2020 Aug 15;217:116884. doi: 10.1016/j.neuroimage.2020.116884 (PMC7378937; doi:10.1016/j.neuroimage.2020.116884)
Supplement: S2: MRI sequence parameters [file mmc3.pdf]

Supplementary material S2
MRI sequence parameters

This document reports the full set of MRI sequence parameters used for both vendors:

- **vendor 1: 3T Philips Achieva, page 1;**
- **vendor 2: 3T Siemens Prisma, page 54.**

Vendor 1 (3T Philips Achieva)

*******   3D FFE T2*-weighted anatomical   *******

Multi-transmit = "yes";
  Active channels = "both";
Nucleus = "H1";
Coil selection = "SENSE-NV-16";
  element selection = "HNACPC";
  connection = "d";
Dual coil = "no";
CLEAR = "yes";
  body tuned = "no";
FOV RL (mm) = 180;
  AP (mm) = 240;
  FH (mm) = 60;
Voxel size RL (mm) = 0.75;
  AP (mm) = 0.75;
  FH (mm) = 5;
Recon voxel size (mm) = 0.75;
Fold-over suppression = "yes";
Slice oversampling = "default";
RF select. FOS = "no";
Reconstruction matrix = 320;
SENSE = "no";
k-t BLAST = "no";
Overcontiguous slices = "no";
Stacks = 1;
  slices = 12;
  slice orientation = "transverse";
  fold-over direction = "AP";
  fat shift direction = "L";
Stack Offc. AP (P=+mm) = -0.332532346;
  RL (L=+mm) = 2.41664815;
  FH (H=+mm) = 9.80785444e-007;
  Ang. AP (deg) = 0;
  RL (deg) = -4.24361372;

FH (deg) = 0;
Chunks = 1;
Large table movement = "no";
PlanAlign = "no";
REST slabs = 1;
type = "free";
orientation = "coronal";
thickness (mm) = 80;
Rest Offc. AP (P=+mm) = -104.381271;
RL (L=+mm) = -15.0501671;
FH (H=+mm) = 15.2842817;
Ang. AP (deg) = 0;
RL (deg) = -0.0405622572;
FH (deg) = 0;
power = "1";
Shim Size AP (mm) = 54.6583862;
RL (mm) = 56.4545441;
FH (mm) = 80;
Offc. AP (P=+mm) = -0.595884502;
RL (L=+mm) = 2.29769874;
FH (H=+mm) = 0.0195419118;
Ang. AP (deg) = 0;
RL (deg) = -4.24361372;
FH (deg) = 0;
Catheter tracking = "no";
Interactive positioning = "no";
Allow table movement = "no";
Patient position = "head first";
orientation = "supine";
Scan type = "Imaging";
Scan mode = "3D";
technique = "FFE";
loop order = "zy_order";
Contrast enhancement = "no";
Acquisition mode = "cartesian";
Fast Imaging mode = "none";
3D non-selective = "no";
Echoes = 1;
partial echo = "yes";
shifted echo = "no";
TE = "shortest";
Flip angle (deg) = 7;
TR = "shortest";
Halfscan = "no";
Water-fat shift = "maximum";
RF Shims = "adaptive";
Shim = "PB-volume";

ShimAlign = "yes";
mDIXON = "no";
Fat suppression = "ProSet";
pulse type = "1331";
Water suppression = "no";
MTC = "no";
Research prepulse = "no";
Diffusion mode = "no";
Elastography mode = "no";
SAR mode = "high";
B1 mode = "default";
SAR Patient data = "auto";
PNS mode = "low";
Gradient mode = "default";
SofTone mode = "no";
Cardiac synchronization = "no";
Heart rate > 250 bpm = "no";
Respiratory compensation = "no";
Navigator respiratory comp = "no";
Flow compensation = "yes";
fMRI echo stabilisation = "no";
NSA = 3;
SMART = "yes";
Angio / Contrast enh. = "no";
Quantitative flow = "no";
Manual start = "no";
Dynamic study = "no";
Arterial Spin labeling = "no";
Preparation phases = "auto";
Interactive F0 = "no";
SmartPlan survey = "no";
B0 field map = "no";
B1 field map = "no";
MIP/MPR = "no";
Images = " M", (3) " no";
Autoview image = " M";
Calculated images = (4) " no";
Reference tissue = "Skeletal muscle";
Preset window contrast = "hard";
Reconstruction mode = "real time";
Save raw data = "no";
Hardcopy protocol = "no";
Ringing filtering = "rectangular";
Geometry correction = "default";
Elliptical k-space shutter = "default";
IF_info_seperator = 1634755923;
Total scan duration = "03:29.5";

Rel. signal level (%) = 100;
Act. TR/TE (ms) = "20 / 4.1";
ACQ matrix M x P = "240 x 320";
ACQ voxel MPS (mm) = "0.75 / 0.75 / 5.00";
REC voxel MPS (mm) = "0.75 / 0.75 / 5.00";
Scan percentage (%) = 100;
Act. WFS (pix) / BW (Hz) = "3.026 / 143.5";
Min. WFS (pix) / Max. BW (Hz) = "0.648 / 670.6";
SAR / local torso = "< 100 %";
Whole body / level = "< 1.6 W/kg / normal";
B1 rms = "2.18 uT / 87 %";
PNS / level = "57 % / normal";
Sound Pressure Level (dB) = 17.3657188;

******* B0 field map *******

Multi-transmit = "no";
Nucleus = "H1";
Coil selection = "Q-Body";
element selection = "12";
Dual coil = "no";
Homogeneity correction = "none";
FOV RL (mm) = 215;
AP (mm) = 206.041672;
FH (mm) = 60;
Voxel size RL (mm) = 2.25;
AP (mm) = 2.25;
FH (mm) = 5;
Recon voxel size (mm) = 2.23958325;
Fold-over suppression = "no";
Slice oversampling = "default";
RF select. FOS = "no";
Reconstruction matrix = 96;
SENSE = "no";
k-t BLAST = "no";
Overcontiguous slices = "no";
Stacks = 1;
slices = 12;
slice orientation = "transverse";
fold-over direction = "AP";
fat shift direction = "L";
Stack Offc. AP (P=+mm) = -0.332532346;
RL (L=+mm) = 2.41664815;
FH (H=+mm) = 9.80785444e-007;
Ang. AP (deg) = 0;
RL (deg) = -4.24361372;

FH (deg) = 0;
Chunks = 1;
Large table movement = "no";
PlanAlign = "no";
REST slabs = 0;
Shim Size AP (mm) = 54.6583862;
RL (mm) = 56.4545441;
FH (mm) = 80;
Offc. AP (P=+mm) = -0.595884502;
RL (L=+mm) = 2.29769874;
FH (H=+mm) = 0.0195419118;
Ang. AP (deg) = 0;
RL (deg) = -4.24361372;
FH (deg) = 0;
Catheter tracking = "no";
Interactive positioning = "no";
Allow table movement = "no";
Patient position = "head first";
orientation = "supine";
Scan type = "Imaging";
Scan mode = "3D";
technique = "FFE";
loop order = "zy_order";
Contrast enhancement = "T1";
Acquisition mode = "cartesian";
Fast Imaging mode = "none";
3D non-selective = "no";
Echoes = 1;
partial echo = "no";
shifted echo = "no";
TE = "in-phase";
(ms) = 6.90782404;
Flip angle (deg) = 25;
TR = "user defined";
(ms) = 50;
Halfscan = "no";
Water-fat shift = "maximum";
Shim = "PB-volume";
ShimAlign = "yes";
mDIXON = "no";
Fat suppression = "no";
Water suppression = "no";
MTC = "no";
Research prepulse = "no";
Diffusion mode = "no";
Elastography mode = "no";
SAR mode = "high";

B1 mode = "default";
SAR Patient data = "auto";
PNS mode = "high";
Gradient mode = "default";
SofTone mode = "no";
Cardiac synchronization = "no";
Heart rate > 250 bpm = "no";
Respiratory compensation = "no";
Navigator respiratory comp = "no";
Flow compensation = "no";
fMRI echo stabilisation = "no";
NSA = 1;
Angio / Contrast enh. = "no";
Quantitative flow = "no";
Manual start = "no";
Dynamic study = "no";
Arterial Spin labeling = "no";
Preparation phases = "auto";
Interactive F0 = "no";
SmartPlan survey = "no";
B0 field map = "yes";
delta TE (ms) = 2.29999995;
B1 field map = "no";
MIP/MPR = "no";
Images = " M", " R", " I", " P";
Autoview image = " M";
Calculated images = (4) " no";
Reference tissue = "Skeletal muscle";
Preset window contrast = "soft";
Reconstruction mode = "real time";
Save raw data = "no";
Hardcopy protocol = "no";
Ringing filtering = "rectangular";
Geometry correction = "default";
Elliptical k-space shutter = "default";
IF_info_seperator = 1634755923;
Total scan duration = "01:39.7";
Rel. signal level (%) = 100;
Act. TR/TE (ms) = "50 / 6.9";
ACQ matrix M x P = "96 x 92";
ACQ voxel MPS (mm) = "2.24 / 2.24 / 5.00";
REC voxel MPS (mm) = "2.24 / 2.24 / 5.00";
Scan percentage (%) = 100;
Act. WFS (pix) / BW (Hz) = "2.862 / 151.7";
Min. WFS (pix) / Max. BW (Hz) = "0.380 / 1144.3";
Min. TR/TE (ms) = "15 / 3.9";
SAR / local torso = "< 15 %";

Whole body / level = "< 0.1 W/kg / normal";
B1 rms = "0.63 uT";
PNS / level = "16 % / normal";
Sound Pressure Level (dB) = 1.37427807;

******* B1 field map *******

Multi-transmit = "yes";
Active channels = "both";
Nucleus = "H1";
Coil selection = "SENSE-NV-16";
element selection = "HNACPC";
connection = "d";
Dual coil = "no";
CLEAR = "yes";
body tuned = "no";
FOV RL (mm) = 215;
AP (mm) = 206.041672;
FH (mm) = 60;
Voxel size RL (mm) = 2.25;
AP (mm) = 2.25;
FH (mm) = 5;
Recon voxel size (mm) = 2.23958325;
Fold-over suppression = "no";
Slice oversampling = "default";
RF select. FOS = "no";
Reconstruction matrix = 96;
SENSE = "no";
k-t BLAST = "no";
Overcontiguous slices = "yes";
Stacks = 1;
slices = 12;
slice orientation = "transverse";
fold-over direction = "AP";
fat shift direction = "L";
Stack Offc. AP (P=+mm) = -0.332532346;
RL (L=+mm) = 2.41664815;
FH (H=+mm) = 9.80785444e-007;
Ang. AP (deg) = 0;
RL (deg) = -4.24361372;
FH (deg) = 0;
Chunks = 1;
Large table movement = "no";
PlanAlign = "no";
REST slabs = 0;
Shim Size AP (mm) = 54.6583862;

RL (mm) = 56.4545441;
FH (mm) = 80;
Offc. AP (P=+mm) = -0.595884502;
RL (L=+mm) = 2.29769874;
FH (H=+mm) = 0.0195419118;
Ang. AP (deg) = 0;
RL (deg) = -4.24361372;
FH (deg) = 0;
Catheter tracking = "no";
Interactive positioning = "no";
Allow table movement = "no";
Patient position = "head first";
orientation = "supine";
Scan type = "Imaging";
Scan mode = "3D";
technique = "FFE";
loop order = "zy_order";
Contrast enhancement = "T1";
Acquisition mode = "cartesian";
Fast Imaging mode = "none";
3D non-selective = "no";
Echoes = 1;
partial echo = "no";
shifted echo = "no";
TE = "shortest";
Flip angle (deg) = 60;
TR = "user defined";
(ms) = 30;
Halfscan = "no";
Water-fat shift = "maximum";
RF Shims = "adaptive";
Shim = "PB-volume";
ShimAlign = "yes";
mDIXON = "no";
Fat suppression = "no";
Water suppression = "no";
MTC = "no";
Research prepulse = "no";
Diffusion mode = "no";
Elastography mode = "no";
SAR mode = "high";
B1 mode = "default";
SAR Patient data = "auto";
PNS mode = "high";
Gradient mode = "default";
SofTone mode = "no";
Cardiac synchronization = "no";

Heart rate > 250 bpm = "no";
Respiratory compensation = "no";
Navigator respiratory comp = "no";
Flow compensation = "no";
fMRI echo stabilisation = "no";
NSA = 1;
Angio / Contrast enh. = "no";
Quantitative flow = "no";
Manual start = "no";
Dynamic study = "no";
Arterial Spin labeling = "no";
Preparation phases = "auto";
Interactive F0 = "select";
SmartPlan survey = "no";
B0 field map = "no";
B1 field map = "yes";
  field map technique = "dual TR";
  TR extension = 120;
  selective = "yes";
  grad spoil factor = 10;
MIP/MPR = "no";
Images = " M", (3) " no";
Autoview image = " M";
Calculated images = " B1", (3) " no";
Reference tissue = "Grey matter";
Preset window contrast = "soft";
Reconstruction mode = "real time";
Save raw data = "no";
Hardcopy protocol = "no";
Ringing filtering = "rectangular";
Geometry correction = "default";
Elliptical k-space shutter = "default";
IF_info_seperator = 1634755923;
Total scan duration = "01:39.9";
Rel. signal level (%) = 100;
Act. TR/TE (ms) = "30 / 2.5";
ACQ matrix M x P = "96 x 92";
ACQ voxel MPS (mm) = "2.24 / 2.24 / 10.0";
REC voxel MPS (mm) = "2.24 / 2.24 / 5.00";
Scan percentage (%) = 100;
Act. WFS (pix) / BW (Hz) = "1.013 / 428.9";
Min. WFS (pix) / Max. BW (Hz) = "0.217 / 2002.5";
Min. TR/TE (ms) = "11 / 2.5";
SAR / local torso = "< 11 %";
Whole body / level = "< 0.2 W/kg / normal";
B1 rms = "0.73 uT / 10 %";
PNS / level = "53 % / normal";

Sound Pressure Level (dB) = 11.9344177;

***** Inversion recovery ZOOM EPI *****

Multi-transmit = "yes";
Active channels = "both";
Nucleus = "H1";
Coil selection = "SENSE-NV-16";
element selection = "HNACPC";
connection = "d";
Dual coil = "no";
CLEAR = "yes";
body tuned = "no";
FOV RL (mm) = 64;
AP (mm) = 48;
FH (mm) = 60;
Voxel size RL (mm) = 1;
AP (mm) = 1;
Slice thickness (mm) = 5;
Recon voxel size (mm) = 1;
Small FOV imaging = "yes";
OVS = "no";
Reconstruction matrix = 64;
SENSE = "no";
k-t BLAST = "no";
Stacks = 1;
type = "parallel";
slices = 12;
slice gap = "user defined";
gap (mm) = 0;
slice orientation = "transverse";
fold-over direction = "AP";
fat shift direction = "P";
Stack Offc. AP (P=+mm) = -0.332532346;
RL (L=+mm) = 2.41664815;
FH (H=+mm) = 9.80785444e-007;
Ang. AP (deg) = 0;
RL (deg) = -4.24361372;
FH (deg) = 0;
Minimum number of packages = 3;
Slice scan order = "interleaved";
Large table movement = "no";
PlanAlign = "no";
REST slabs = 0;
Shim Size AP (mm) = 54.6583862;
RL (mm) = 56.4545441;

FH (mm) =	80;
Offc. AP (P=+mm) =	-0.595884502;
RL (L=+mm) =	2.29769874;
FH (H=+mm) =	0.0195419118;
Ang. AP (deg) =	0;
RL (deg) =	-4.24361372;
FH (deg) =	0;
Catheter tracking =	"no";
Interactive positioning =	"no";
Allow table movement =	"no";
Patient position =	"head first";
orientation =	"supine";
Scan type =	"Imaging";
Scan mode =	"MS";
technique =	"SE";
Modified SE =	"no";
Acquisition mode =	"cartesian";
Fast Imaging mode =	"EPI";
shot mode =	"single-shot";
Echoes =	1;
partial echo =	"no";
TE =	"shortest";
Flip angle (deg) =	90;
TR =	"shortest";
Halfscan =	"yes";
factor =	0.600000024;
Water-fat shift =	"minimum";
shared inversion prepulse =	"yes";
Number of TIs =	3;
Inversion times =	100, 900, 1700, (12) 0;
package slices shuffling =	"yes";
RF Shims =	"adaptive";
Shim =	"PB-volume";
ShimAlign =	"yes";
mDIXON =	"no";
Fat suppression =	"SPIR";
strength =	"strong";
frequency offset =	"default";
Water suppression =	"no";
Grad. rev. offres. supp. =	"yes";
BB pulse =	"no";
MTR optimal =	"no";
MTC =	"no";
Research prepulse =	"no";
Diffusion mode =	"no";
Elastography mode =	"no";

SAR mode = "high";
B1 mode = "default";
SAR Patient data = "auto";
PNS mode = "high";
Gradient mode = "enhanced";
SofTone mode = "no";
Cardiac synchronization = "no";
Heart rate > 250 bpm = "no";
Respiratory compensation = "no";
Navigator respiratory comp = "no";
Flow compensation = "yes";
Temporal slice spacing = "user defined";
Slice interval = 200;
Package recovery time = "user defined";
Recovery time = 6000;
NSA = 1;
Manual start = "no";
Dynamic study = "individual";
dyn scans = 12;
recon multiplier = 1;
dyn scan times = "shortest";
FOV time mode = "default";
dummy scans = 0;
immediate subtraction = "no";
fast next scan = "no";
synch. ext. device = "no";
Noise scan = "no";
dyn stabilization = "no";
prospect. motion corr. = "no";
Keyhole = "no";
Arterial Spin labeling = "no";
Preparation phases = "auto";
Interactive F0 = "no";
SmartPlan survey = "no";
B0 field map = "no";
B1 field map = "no";
MIP/MPR = "no";
Images = " M", " R", " I", " no";
Autoview image = " M";
Calculated images = (4) " no";
Reference tissue = "Grey matter";
EPI 2D phase correction = "no";
Preset window contrast = "soft";
Reconstruction mode = "immediate";
Save raw data = "yes";
Hardcopy protocol = "no";
Ringing filtering = "rectangular";

Geometry correction =	"default";
IF_info_seperator =	1634755923;
Total scan duration =	"04:21.5";
Rel. signal level (%) =	100;
Act. TR (ms) =	"6705";
Act. TE (ms) =	"24";
Dyn. scan time =	"00:20.1";
ACQ matrix M x P =	"64 x 47";
ACQ voxel MPS (mm) =	"1.00 / 1.01 / 5.00";
REC voxel MPS (mm) =	"1.00 / 1.00 / 5.00";
Scan percentage (%) =	98.6111145;
Packages =	3;
Min. slice gap (mm) =	0;
EPI factor =	71;
WFS (pix) / BW (Hz) =	"30.973 / 14.0";
BW in EPI freq. dir. (Hz) =	"2100.3";
Full flow comp. =	"no";
SAR / local torso =	"< 4 %";
Whole body / level =	"< 0.1 W/kg / normal";
B1 rms =	"0.45 uT / 4 %";
PNS / level =	"91 % / 1st level";
Sound Pressure Level (dB) =	13.1894379;

******* Multi-TE ZOOM EPI, 1st TE *******

Multi-transmit =	"yes";
Active channels =	"both";
Nucleus =	"H1";
Coil selection =	"SENSE-NV-16";
element selection =	"HNACPC";
connection =	"d";
Dual coil =	"no";
CLEAR =	"yes";
body tuned =	"no";
FOV RL (mm) =	64;
AP (mm) =	48;
FH (mm) =	60;
Voxel size RL (mm) =	1;
AP (mm) =	1;
Slice thickness (mm) =	5;
Recon voxel size (mm) =	1;
Small FOV imaging =	"yes";
OVS =	"no";
Reconstruction matrix =	64;
SENSE =	"no";
k-t BLAST =	"no";

Stacks = 1;
type = "parallel";
slices = 12;
slice gap = "user defined";
gap (mm) = 0;
slice orientation = "transverse";
fold-over direction = "AP";
fat shift direction = "P";
Stack Offc. AP (P=+mm) = -0.332532346;
RL (L=+mm) = 2.41664815;
FH (H=+mm) = 9.80785444e-007;
Ang. AP (deg) = 0;
RL (deg) = -4.24361372;
FH (deg) = 0;
Minimum number of packages = 3;
Slice scan order = "interleaved";
Large table movement = "no";
PlanAlign = "no";
REST slabs = 0;
Shim Size AP (mm) = 54.6583862;
RL (mm) = 56.4545441;
FH (mm) = 80;
Offc. AP (P=+mm) = -0.595884502;
RL (L=+mm) = 2.29769874;
FH (H=+mm) = 0.0195419118;
Ang. AP (deg) = 0;
RL (deg) = -4.24361372;
FH (deg) = 0;
Catheter tracking = "no";
Interactive positioning = "no";
Allow table movement = "no";
Patient position = "head first";
orientation = "supine";
Scan type = "Imaging";
Scan mode = "MS";
technique = "SE";
Modified SE = "no";
Acquisition mode = "cartesian";
Fast Imaging mode = "EPI";
shot mode = "single-shot";
Echoes = 1;
partial echo = "no";
TE = "user defined";
(ms) = 25;
Flip angle (deg) = 90;
TR = "user defined";
(ms) = 4000;

Halfscan =	"yes";
factor =	0.600000024;
Water-fat shift =	"minimum";
shared inversion prepulse =	"no";
RF Shims =	"adaptive";
Shim =	"PB-volume";
ShimAlign =	"yes";
mDIXON =	"no";
Fat suppression =	"SPIR";
strength =	"strong";
frequency offset =	"default";
Water suppression =	"no";
Grad. rev. offres. supp. =	"no";
BB pulse =	"no";
MTR optimal =	"no";
MTC =	"no";
Research prepulse =	"no";
Diffusion mode =	"no";
Elastography mode =	"no";
SAR mode =	"high";
B1 mode =	"default";
SAR Patient data =	"auto";
PNS mode =	"high";
Gradient mode =	"enhanced";
SofTone mode =	"no";
Cardiac synchronization =	"no";
Heart rate > 250 bpm =	"no";
Respiratory compensation =	"no";
Navigator respiratory comp =	"no";
Flow compensation =	"no";
Temporal slice spacing =	"default";
Package recovery time =	"default";
NSA =	1;
Manual start =	"no";
Dynamic study =	"no";
Arterial Spin labeling =	"no";
Preparation phases =	"auto";
Interactive F0 =	"no";
SmartPlan survey =	"no";
B0 field map =	"no";
B1 field map =	"no";
MIP/MPR =	"no";
Images =	" M", " R", " I", " no";
Autoview image =	" M";
Calculated images =	(4) " no";
Reference tissue =	"White matter";
EPI 2D phase correction =	"no";

Preset window contrast =	"soft";
Reconstruction mode =	"immediate";
Save raw data =	"yes";
Hardcopy protocol =	"no";
Ringing filtering =	"rectangular";
Geometry correction =	"default";
IF_info_seperator =	1634755923;
Total scan duration =	"00:24.0";
Rel. signal level (%) =	100;
Act. TR (ms) =	"4000";
Act. TE (ms) =	"25";
ACQ matrix M x P =	"64 x 47";
ACQ voxel MPS (mm) =	"1.00 / 1.01 / 5.00";
REC voxel MPS (mm) =	"1.00 / 1.00 / 5.00";
Scan percentage (%) =	98.6111145;
Packages =	3;
Min. slice gap (mm) =	0;
Optimal slices =	4;
EPI factor =	71;
WFS (pix) / BW (Hz) =	"30.973 / 14.0";
BW in EPI freq. dir. (Hz) =	"2100.3";
Min. TR (ms) =	"316";
SAR / local torso =	"< 5 %";
Whole body / level =	"< 0.1 W/kg / normal";
B1 rms =	"0.48 uT / 4 %";
PNS / level =	"91 % / 1st level";
Sound Pressure Level (dB) =	12.1325197;

***** Multi-TE ZOOM EPI, 2nd TE *****

Multi-transmit =	"yes";
Active channels =	"both";
Nucleus =	"H1";
Coil selection =	"SENSE-NV-16";
element selection =	"HNACPC";
connection =	"d";
Dual coil =	"no";
CLEAR =	"yes";
body tuned =	"no";
FOV RL (mm) =	64;
AP (mm) =	48;
FH (mm) =	60;
Voxel size RL (mm) =	1;
AP (mm) =	1;
Slice thickness (mm) =	5;
Recon voxel size (mm) =	1;

Small FOV imaging = "yes";
OVS = "no";
Reconstruction matrix = 64;
SENSE = "no";
k-t BLAST = "no";
Stacks = 1;
type = "parallel";
slices = 12;
slice gap = "user defined";
gap (mm) = 0;
slice orientation = "transverse";
fold-over direction = "AP";
fat shift direction = "P";
Stack Offc. AP (P=+mm) = -0.332532346;
RL (L=+mm) = 2.41664815;
FH (H=+mm) = 9.80785444e-007;
Ang. AP (deg) = 0;
RL (deg) = -4.24361372;
FH (deg) = 0;
Minimum number of packages = 3;
Slice scan order = "interleaved";
Large table movement = "no";
PlanAlign = "no";
REST slabs = 0;
Shim Size AP (mm) = 54.6583862;
RL (mm) = 56.4545441;
FH (mm) = 80;
Offc. AP (P=+mm) = -0.595884502;
RL (L=+mm) = 2.29769874;
FH (H=+mm) = 0.0195419118;
Ang. AP (deg) = 0;
RL (deg) = -4.24361372;
FH (deg) = 0;
Catheter tracking = "no";
Interactive positioning = "no";
Allow table movement = "no";
Patient position = "head first";
orientation = "supine";
Scan type = "Imaging";
Scan mode = "MS";
technique = "SE";
Modified SE = "no";
Acquisition mode = "cartesian";
Fast Imaging mode = "EPI";
shot mode = "single-shot";
Echoes = 1;
partial echo = "no";

TE = "user defined";
(ms) = 40;
Flip angle (deg) = 90;
TR = "user defined";
(ms) = 4000;
Halfscan = "yes";
factor = 0.600000024;
Water-fat shift = "minimum";
shared inversion prepulse = "no";
RF Shims = "adaptive";
Shim = "PB-volume";
ShimAlign = "yes";
mDIXON = "no";
Fat suppression = "SPIR";
strength = "strong";
frequency offset = "default";
Water suppression = "no";
Grad. rev. offres. supp. = "no";
BB pulse = "no";
MTR optimal = "no";
MTC = "no";
Research prepulse = "no";
Diffusion mode = "no";
Elastography mode = "no";
SAR mode = "high";
B1 mode = "default";
SAR Patient data = "auto";
PNS mode = "high";
Gradient mode = "enhanced";
SofTone mode = "no";
Cardiac synchronization = "no";
Heart rate > 250 bpm = "no";
Respiratory compensation = "no";
Navigator respiratory comp = "no";
Flow compensation = "no";
Temporal slice spacing = "default";
Package recovery time = "default";
NSA = 1;
Manual start = "no";
Dynamic study = "no";
Arterial Spin labeling = "no";
Preparation phases = "auto";
Interactive F0 = "no";
SmartPlan survey = "no";
B0 field map = "no";
B1 field map = "no";
MIP/MPR = "no";

Images = " M", " R", " I", " no";
Autoview image = " M";
Calculated images = (4) " no";
Reference tissue = "White matter";
EPI 2D phase correction = "no";
Preset window contrast = "soft";
Reconstruction mode = "immediate";
Save raw data = "yes";
Hardcopy protocol = "no";
Ringing filtering = "rectangular";
Geometry correction = "default";
IF_info_seperator = 1634755923;
Total scan duration = "00:24.0";
Rel. signal level (%) = 100;
Act. TR (ms) = "4000";
Act. TE (ms) = "40";
ACQ matrix M x P = "64 x 47";
ACQ voxel MPS (mm) = "1.00 / 1.01 / 5.00";
REC voxel MPS (mm) = "1.00 / 1.00 / 5.00";
Scan percentage (%) = 98.6111145;
Packages = 3;
Min. slice gap (mm) = 0;
Optimal slices = 4;
EPI factor = 71;
WFS (pix) / BW (Hz) = "30.973 / 14.0";
BW in EPI freq. dir. (Hz) = "2100.3";
Min. TR (ms) = "376";
SAR / local torso = "< 5 %";
Whole body / level = "< 0.1 W/kg / normal";
B1 rms = "0.48 uT / 4 %";
PNS / level = "91 % / 1st level";
Sound Pressure Level (dB) = 12.6679831;

*******   Multi-TE ZOOM EPI, 3rd TE   *******

Multi-transmit = "yes";
Active channels = "both";
Nucleus = "H1";
Coil selection = "SENSE-NV-16";
element selection = "HNACPC";
connection = "d";
Dual coil = "no";
CLEAR = "yes";
body tuned = "no";
FOV RL (mm) = 64;
AP (mm) = 48;

FH (mm) = 60;
Voxel size RL (mm) = 1;
AP (mm) = 1;
Slice thickness (mm) = 5;
Recon voxel size (mm) = 1;
Small FOV imaging = "yes";
OVS = "no";
Reconstruction matrix = 64;
SENSE = "no";
k-t BLAST = "no";
Stacks = 1;
type = "parallel";
slices = 12;
slice gap = "user defined";
gap (mm) = 0;
slice orientation = "transverse";
fold-over direction = "AP";
fat shift direction = "P";
Stack Offc. AP (P=+mm) = -0.332532346;
RL (L=+mm) = 2.41664815;
FH (H=+mm) = 9.80785444e-007;
Ang. AP (deg) = 0;
RL (deg) = -4.24361372;
FH (deg) = 0;
Minimum number of packages = 3;
Slice scan order = "interleaved";
Large table movement = "no";
PlanAlign = "no";
REST slabs = 0;
Shim Size AP (mm) = 54.6583862;
RL (mm) = 56.4545441;
FH (mm) = 80;
Offc. AP (P=+mm) = -0.595884502;
RL (L=+mm) = 2.29769874;
FH (H=+mm) = 0.0195419118;
Ang. AP (deg) = 0;
RL (deg) = -4.24361372;
FH (deg) = 0;
Catheter tracking = "no";
Interactive positioning = "no";
Allow table movement = "no";
Patient position = "head first";
orientation = "supine";
Scan type = "Imaging";
Scan mode = "MS";
technique = "SE";
Modified SE = "no";

Acquisition mode =	"cartesian";
Fast Imaging mode =	"EPI";
shot mode =	"single-shot";
Echoes =	1;
partial echo =	"no";
TE =	"user defined";
(ms) =	55;
Flip angle (deg) =	90;
TR =	"user defined";
(ms) =	4000;
Halfscan =	"yes";
factor =	0.600000024;
Water-fat shift =	"minimum";
shared inversion prepulse =	"no";
RF Shims =	"adaptive";
Shim =	"PB-volume";
ShimAlign =	"yes";
mDIXON =	"no";
Fat suppression =	"SPIR";
strength =	"strong";
frequency offset =	"default";
Water suppression =	"no";
Grad. rev. offres. supp. =	"no";
BB pulse =	"no";
MTR optimal =	"no";
MTC =	"no";
Research prepulse =	"no";
Diffusion mode =	"no";
Elastography mode =	"no";
SAR mode =	"high";
B1 mode =	"default";
SAR Patient data =	"auto";
PNS mode =	"high";
Gradient mode =	"enhanced";
SofTone mode =	"no";
Cardiac synchronization =	"no";
Heart rate > 250 bpm =	"no";
Respiratory compensation =	"no";
Navigator respiratory comp =	"no";
Flow compensation =	"no";
Temporal slice spacing =	"default";
Package recovery time =	"default";
NSA =	1;
Manual start =	"no";
Dynamic study =	"no";
Arterial Spin labeling =	"no";
Preparation phases =	"auto";

Interactive F0 =	"no";
SmartPlan survey =	"no";
B0 field map =	"no";
B1 field map =	"no";
MIP/MPR =	"no";
Images =	" M", " R", " I", " no";
Autoview image =	" M";
Calculated images =	(4) " no";
Reference tissue =	"White matter";
EPI 2D phase correction =	"no";
Preset window contrast =	"soft";
Reconstruction mode =	"immediate";
Save raw data =	"yes";
Hardcopy protocol =	"no";
Ringing filtering =	"rectangular";
Geometry correction =	"default";
IF_info_seperator =	1634755923;
Total scan duration =	"00:24.0";
Rel. signal level (%) =	100;
Act. TR (ms) =	"4000";
Act. TE (ms) =	"55";
ACQ matrix M x P =	"64 x 47";
ACQ voxel MPS (mm) =	"1.00 / 1.01 / 5.00";
REC voxel MPS (mm) =	"1.00 / 1.00 / 5.00";
Scan percentage (%) =	98.6111145;
Packages =	3;
Min. slice gap (mm) =	0;
Optimal slices =	4;
EPI factor =	71;
WFS (pix) / BW (Hz) =	"30.973 / 14.0";
BW in EPI freq. dir. (Hz) =	"2100.3";
Min. TR (ms) =	"436";
SAR / local torso =	"< 5 %";
Whole body / level =	"< 0.1 W/kg / normal";
B1 rms =	"0.48 uT / 4 %";
PNS / level =	"91 % / 1st level";
Sound Pressure Level (dB) =	12.1785688;

******* Multi-TE ZOOM EPI, 4th TE *******

Multi-transmit =	"yes";
Active channels =	"both";
Nucleus =	"H1";
Coil selection =	"SENSE-NV-16";
element selection =	"HNACPC";
connection =	"d";

Dual coil = "no";
CLEAR = "yes";
body tuned = "no";
FOV RL (mm) = 64;
AP (mm) = 48;
FH (mm) = 60;
Voxel size RL (mm) = 1;
AP (mm) = 1;
Slice thickness (mm) = 5;
Recon voxel size (mm) = 1;
Small FOV imaging = "yes";
OVS = "no";
Reconstruction matrix = 64;
SENSE = "no";
k-t BLAST = "no";
Stacks = 1;
type = "parallel";
slices = 12;
slice gap = "user defined";
gap (mm) = 0;
slice orientation = "transverse";
fold-over direction = "AP";
fat shift direction = "P";
Stack Offc. AP (P=+mm) = -0.332532346;
RL (L=+mm) = 2.41664815;
FH (H=+mm) = 9.80785444e-007;
Ang. AP (deg) = 0;
RL (deg) = -4.24361372;
FH (deg) = 0;
Minimum number of packages = 3;
Slice scan order = "interleaved";
Large table movement = "no";
PlanAlign = "no";
REST slabs = 0;
Shim Size AP (mm) = 54.6583862;
RL (mm) = 56.4545441;
FH (mm) = 80;
Offc. AP (P=+mm) = -0.595884502;
RL (L=+mm) = 2.29769874;
FH (H=+mm) = 0.0195419118;
Ang. AP (deg) = 0;
RL (deg) = -4.24361372;
FH (deg) = 0;
Catheter tracking = "no";
Interactive positioning = "no";
Allow table movement = "no";
Patient position = "head first";

orientation =	"supine";
Scan type =	"Imaging";
Scan mode =	"MS";
technique =	"SE";
Modified SE =	"no";
Acquisition mode =	"cartesian";
Fast Imaging mode =	"EPI";
shot mode =	"single-shot";
Echoes =	1;
partial echo =	"no";
TE =	"user defined";
(ms) =	70;
Flip angle (deg) =	90;
TR =	"user defined";
(ms) =	4000;
Halfscan =	"yes";
factor =	0.600000024;
Water-fat shift =	"minimum";
shared inversion prepulse =	"no";
RF Shims =	"adaptive";
Shim =	"PB-volume";
ShimAlign =	"yes";
mDIXON =	"no";
Fat suppression =	"SPIR";
strength =	"strong";
frequency offset =	"default";
Water suppression =	"no";
Grad. rev. offres. supp. =	"no";
BB pulse =	"no";
MTR optimal =	"no";
MTC =	"no";
Research prepulse =	"no";
Diffusion mode =	"no";
Elastography mode =	"no";
SAR mode =	"high";
B1 mode =	"default";
SAR Patient data =	"auto";
PNS mode =	"high";
Gradient mode =	"enhanced";
SofTone mode =	"no";
Cardiac synchronization =	"no";
Heart rate > 250 bpm =	"no";
Respiratory compensation =	"no";
Navigator respiratory comp =	"no";
Flow compensation =	"no";
Temporal slice spacing =	"default";
Package recovery time =	"default";

NSA =	1;
Manual start =	"no";
Dynamic study =	"no";
Arterial Spin labeling =	"no";
Preparation phases =	"auto";
Interactive F0 =	"no";
SmartPlan survey =	"no";
B0 field map =	"no";
B1 field map =	"no";
MIP/MPR =	"no";
Images =	" M", " R", " I", " no";
Autoview image =	" M";
Calculated images =	(4) " no";
Reference tissue =	"White matter";
EPI 2D phase correction =	"no";
Preset window contrast =	"soft";
Reconstruction mode =	"immediate";
Save raw data =	"yes";
Hardcopy protocol =	"no";
Ringing filtering =	"rectangular";
Geometry correction =	"default";
IF_info_seperator =	1634755923;
Total scan duration =	"00:24.0";
Rel. signal level (%) =	100;
Act. TR (ms) =	"4000";
Act. TE (ms) =	"70";
ACQ matrix M x P =	"64 x 47";
ACQ voxel MPS (mm) =	"1.00 / 1.01 / 5.00";
REC voxel MPS (mm) =	"1.00 / 1.00 / 5.00";
Scan percentage (%) =	98.6111145;
Packages =	3;
Min. slice gap (mm) =	0;
Optimal slices =	4;
EPI factor =	71;
WFS (pix) / BW (Hz) =	"30.973 / 14.0";
BW in EPI freq. dir. (Hz) =	"2100.3";
Min. TR (ms) =	"496";
SAR / local torso =	"< 5 %";
Whole body / level =	"< 0.1 W/kg / normal";
B1 rms =	"0.48 uT / 4 %";
PNS / level =	"91 % / 1st level";
Sound Pressure Level (dB) =	12.1020241;

******* Multi-TE ZOOM EPI, 5th TE *******

Multi-transmit =	"yes";

Active channels = "both";
Nucleus = "H1";
Coil selection = "SENSE-NV-16";
element selection = "HNACPC";
connection = "d";
Dual coil = "no";
CLEAR = "yes";
body tuned = "no";
FOV RL (mm) = 64;
AP (mm) = 48;
FH (mm) = 60;
Voxel size RL (mm) = 1;
AP (mm) = 1;
Slice thickness (mm) = 5;
Recon voxel size (mm) = 1;
Small FOV imaging = "yes";
OVS = "no";
Reconstruction matrix = 64;
SENSE = "no";
k-t BLAST = "no";
Stacks = 1;
type = "parallel";
slices = 12;
slice gap = "user defined";
gap (mm) = 0;
slice orientation = "transverse";
fold-over direction = "AP";
fat shift direction = "P";
Stack Offc. AP (P=+mm) = -0.332532346;
RL (L=+mm) = 2.41664815;
FH (H=+mm) = 9.80785444e-007;
Ang. AP (deg) = 0;
RL (deg) = -4.24361372;
FH (deg) = 0;
Minimum number of packages = 3;
Slice scan order = "interleaved";
Large table movement = "no";
PlanAlign = "no";
REST slabs = 0;
Shim Size AP (mm) = 54.6583862;
RL (mm) = 56.4545441;
FH (mm) = 80;
Offc. AP (P=+mm) = -0.595884502;
RL (L=+mm) = 2.29769874;
FH (H=+mm) = 0.0195419118;
Ang. AP (deg) = 0;
RL (deg) = -4.24361372;

FH (deg) =	0;
Catheter tracking =	"no";
Interactive positioning =	"no";
Allow table movement =	"no";
Patient position =	"head first";
orientation =	"supine";
Scan type =	"Imaging";
Scan mode =	"MS";
technique =	"SE";
Modified SE =	"no";
Acquisition mode =	"cartesian";
Fast Imaging mode =	"EPI";
shot mode =	"single-shot";
Echoes =	1;
partial echo =	"no";
TE =	"user defined";
(ms) =	85;
Flip angle (deg) =	90;
TR =	"user defined";
(ms) =	4000;
Halfscan =	"yes";
factor =	0.600000024;
Water-fat shift =	"minimum";
shared inversion prepulse =	"no";
RF Shims =	"adaptive";
Shim =	"PB-volume";
ShimAlign =	"yes";
mDIXON =	"no";
Fat suppression =	"SPIR";
strength =	"strong";
frequency offset =	"default";
Water suppression =	"no";
Grad. rev. offres. supp. =	"no";
BB pulse =	"no";
MTR optimal =	"no";
MTC =	"no";
Research prepulse =	"no";
Diffusion mode =	"no";
Elastography mode =	"no";
SAR mode =	"high";
B1 mode =	"default";
SAR Patient data =	"auto";
PNS mode =	"high";
Gradient mode =	"enhanced";
SofTone mode =	"no";
Cardiac synchronization =	"no";
Heart rate > 250 bpm =	"no";

Respiratory compensation = "no";
Navigator respiratory comp = "no";
Flow compensation = "no";
Temporal slice spacing = "default";
Package recovery time = "default";
NSA = 1;
Manual start = "no";
Dynamic study = "no";
Arterial Spin labeling = "no";
Preparation phases = "auto";
Interactive F0 = "no";
SmartPlan survey = "no";
B0 field map = "no";
B1 field map = "no";
MIP/MPR = "no";
Images = " M", " R", " I", " no";
Autoview image = " M";
Calculated images = (4) " no";
Reference tissue = "White matter";
EPI 2D phase correction = "no";
Preset window contrast = "soft";
Reconstruction mode = "immediate";
Save raw data = "yes";
Hardcopy protocol = "no";
Ringing filtering = "rectangular";
Geometry correction = "default";
IF_info_seperator = 1634755923;
Total scan duration = "00:24.0";
Rel. signal level (%) = 100;
Act. TR (ms) = "4000";
Act. TE (ms) = "85";
ACQ matrix M x P = "64 x 47";
ACQ voxel MPS (mm) = "1.00 / 1.01 / 5.00";
REC voxel MPS (mm) = "1.00 / 1.00 / 5.00";
Scan percentage (%) = 98.6111145;
Packages = 3;
Min. slice gap (mm) = 0;
Optimal slices = 4;
EPI factor = 71;
WFS (pix) / BW (Hz) = "30.973 / 14.0";
BW in EPI freq. dir. (Hz) = "2100.3";
Min. TR (ms) = "556";
SAR / local torso = "< 5 %";
Whole body / level = "< 0.1 W/kg / normal";
B1 rms = "0.48 uT / 4 %";
PNS / level = "91 % / 1st level";
Sound Pressure Level (dB) = 12.2095785;

******* Multi-TE ZOOM EPI, 6th TE *******

Multi-transmit = "yes";
Active channels = "both";
Nucleus = "H1";
Coil selection = "SENSE-NV-16";
element selection = "HNACPC";
connection = "d";
Dual coil = "no";
CLEAR = "yes";
body tuned = "no";
FOV RL (mm) = 64;
AP (mm) = 48;
FH (mm) = 60;
Voxel size RL (mm) = 1;
AP (mm) = 1;
Slice thickness (mm) = 5;
Recon voxel size (mm) = 1;
Small FOV imaging = "yes";
OVS = "no";
Reconstruction matrix = 64;
SENSE = "no";
k-t BLAST = "no";
Stacks = 1;
type = "parallel";
slices = 12;
slice gap = "user defined";
gap (mm) = 0;
slice orientation = "transverse";
fold-over direction = "AP";
fat shift direction = "P";
Stack Offc. AP (P=+mm) = -0.332532346;
RL (L=+mm) = 2.41664815;
FH (H=+mm) = 9.80785444e-007;
Ang. AP (deg) = 0;
RL (deg) = -4.24361372;
FH (deg) = 0;
Minimum number of packages = 3;
Slice scan order = "interleaved";
Large table movement = "no";
PlanAlign = "no";
REST slabs = 0;
Shim Size AP (mm) = 54.6583862;
RL (mm) = 56.4545441;
FH (mm) = 80;

Offc. AP (P=+mm) =	-0.595884502;
RL (L=+mm) =	2.29769874;
FH (H=+mm) =	0.0195419118;
Ang. AP (deg) =	0;
RL (deg) =	-4.24361372;
FH (deg) =	0;
Catheter tracking =	"no";
Interactive positioning =	"no";
Allow table movement =	"no";
Patient position =	"head first";
orientation =	"supine";
Scan type =	"Imaging";
Scan mode =	"MS";
technique =	"SE";
Modified SE =	"no";
Acquisition mode =	"cartesian";
Fast Imaging mode =	"EPI";
shot mode =	"single-shot";
Echoes =	1;
partial echo =	"no";
TE =	"user defined";
(ms) =	100;
Flip angle (deg) =	90;
TR =	"user defined";
(ms) =	4000;
Halfscan =	"yes";
factor =	0.600000024;
Water-fat shift =	"minimum";
shared inversion prepulse =	"no";
RF Shims =	"adaptive";
Shim =	"PB-volume";
ShimAlign =	"yes";
mDIXON =	"no";
Fat suppression =	"SPIR";
strength =	"strong";
frequency offset =	"default";
Water suppression =	"no";
Grad. rev. offres. supp. =	"no";
BB pulse =	"no";
MTR optimal =	"no";
MTC =	"no";
Research prepulse =	"no";
Diffusion mode =	"no";
Elastography mode =	"no";
SAR mode =	"high";
B1 mode =	"default";
SAR Patient data =	"auto";

PNS mode =	"high";
Gradient mode =	"enhanced";
SofTone mode =	"no";
Cardiac synchronization =	"no";
Heart rate > 250 bpm =	"no";
Respiratory compensation =	"no";
Navigator respiratory comp =	"no";
Flow compensation =	"no";
Temporal slice spacing =	"default";
Package recovery time =	"default";
NSA =	1;
Manual start =	"no";
Dynamic study =	"no";
Arterial Spin labeling =	"no";
Preparation phases =	"auto";
Interactive F0 =	"no";
SmartPlan survey =	"no";
B0 field map =	"no";
B1 field map =	"no";
MIP/MPR =	"no";
Images =	" M", " R", " I", " no";
Autoview image =	" M";
Calculated images =	(4) " no";
Reference tissue =	"White matter";
EPI 2D phase correction =	"no";
Preset window contrast =	"soft";
Reconstruction mode =	"immediate";
Save raw data =	"yes";
Hardcopy protocol =	"no";
Ringing filtering =	"rectangular";
Geometry correction =	"default";
IF_info_seperator =	1634755923;
Total scan duration =	"00:24.0";
Rel. signal level (%) =	100;
Act. TR (ms) =	"4000";
Act. TE (ms) =	"100";
ACQ matrix M x P =	"64 x 47";
ACQ voxel MPS (mm) =	"1.00 / 1.01 / 5.00";
REC voxel MPS (mm) =	"1.00 / 1.00 / 5.00";
Scan percentage (%) =	98.6111145;
Packages =	3;
Min. slice gap (mm) =	0;
Optimal slices =	4;
EPI factor =	71;
WFS (pix) / BW (Hz) =	"30.973 / 14.0";
BW in EPI freq. dir. (Hz) =	"2100.3";
Min. TR (ms) =	"616";

SAR / local torso = "< 5 %";
Whole body / level = "< 0.1 W/kg / normal";
B1 rms = "0.48 uT / 4 %";
PNS / level = "91 % / 1st level";
Sound Pressure Level (dB) = 12.1201229;

******* Multi-TE ZOOM EPI, 7th TE *******

Multi-transmit = "yes";
Active channels = "both";
Nucleus = "H1";
Coil selection = "SENSE-NV-16";
element selection = "HNACPC";
connection = "d";
Dual coil = "no";
CLEAR = "yes";
body tuned = "no";
FOV RL (mm) = 64;
AP (mm) = 48;
FH (mm) = 60;
Voxel size RL (mm) = 1;
AP (mm) = 1;
Slice thickness (mm) = 5;
Recon voxel size (mm) = 1;
Small FOV imaging = "yes";
OVS = "no";
Reconstruction matrix = 64;
SENSE = "no";
k-t BLAST = "no";
Stacks = 1;
type = "parallel";
slices = 12;
slice gap = "user defined";
gap (mm) = 0;
slice orientation = "transverse";
fold-over direction = "AP";
fat shift direction = "P";
Stack Offc. AP (P=+mm) = -0.332532346;
RL (L=+mm) = 2.41664815;
FH (H=+mm) = 9.80785444e-007;
Ang. AP (deg) = 0;
RL (deg) = -4.24361372;
FH (deg) = 0;
Minimum number of packages = 3;
Slice scan order = "interleaved";
Large table movement = "no";

PlanAlign = "no";
REST slabs = 0;
Shim Size AP (mm) = 54.6583862;
RL (mm) = 56.4545441;
FH (mm) = 80;
Offc. AP (P=+mm) = -0.595884502;
RL (L=+mm) = 2.29769874;
FH (H=+mm) = 0.0195419118;
Ang. AP (deg) = 0;
RL (deg) = -4.24361372;
FH (deg) = 0;
Catheter tracking = "no";
Interactive positioning = "no";
Allow table movement = "no";
Patient position = "head first";
orientation = "supine";
Scan type = "Imaging";
Scan mode = "MS";
technique = "SE";
Modified SE = "no";
Acquisition mode = "cartesian";
Fast Imaging mode = "EPI";
shot mode = "single-shot";
Echoes = 1;
partial echo = "no";
TE = "user defined";
(ms) = 200;
Flip angle (deg) = 90;
TR = "user defined";
(ms) = 4000;
Halfscan = "yes";
factor = 0.600000024;
Water-fat shift = "minimum";
shared inversion prepulse = "no";
RF Shims = "adaptive";
Shim = "PB-volume";
ShimAlign = "yes";
mDIXON = "no";
Fat suppression = "SPIR";
strength = "strong";
frequency offset = "default";
Water suppression = "no";
Grad. rev. offres. supp. = "no";
BB pulse = "no";
MTR optimal = "no";
MTC = "no";
Research prepulse = "no";

Diffusion mode =	"no";
Elastography mode =	"no";
SAR mode =	"high";
B1 mode =	"default";
SAR Patient data =	"auto";
PNS mode =	"high";
Gradient mode =	"enhanced";
SofTone mode =	"no";
Cardiac synchronization =	"no";
Heart rate > 250 bpm =	"no";
Respiratory compensation =	"no";
Navigator respiratory comp =	"no";
Flow compensation =	"no";
Temporal slice spacing =	"default";
Package recovery time =	"default";
NSA =	1;
Manual start =	"no";
Dynamic study =	"no";
Arterial Spin labeling =	"no";
Preparation phases =	"auto";
Interactive F0 =	"no";
SmartPlan survey =	"no";
B0 field map =	"no";
B1 field map =	"no";
MIP/MPR =	"no";
Images =	" M", " R", " I", " no";
Autoview image =	" M";
Calculated images =	(4) " no";
Reference tissue =	"White matter";
EPI 2D phase correction =	"no";
Preset window contrast =	"soft";
Reconstruction mode =	"immediate";
Save raw data =	"yes";
Hardcopy protocol =	"no";
Ringing filtering =	"rectangular";
Geometry correction =	"default";
IF_info_seperator =	1634755923;
Total scan duration =	"00:24.0";
Rel. signal level (%) =	100;
Act. TR (ms) =	"4000";
Act. TE (ms) =	"200";
ACQ matrix M x P =	"64 x 47";
ACQ voxel MPS (mm) =	"1.00 / 1.01 / 5.00";
REC voxel MPS (mm) =	"1.00 / 1.00 / 5.00";
Scan percentage (%) =	98.6111145;
Packages =	3;
Min. slice gap (mm) =	0;

Optimal slices = 4;
EPI factor = 71;
WFS (pix) / BW (Hz) = "30.973 / 14.0";
BW in EPI freq. dir. (Hz) = "2100.3";
Min. TR (ms) = "1016";
SAR / local torso = "< 5 %";
Whole body / level = "< 0.1 W/kg / normal";
B1 rms = "0.48 uT / 4 %";
PNS / level = "91 % / 1st level";
Sound Pressure Level (dB) = 11.6565809;

******* DW ZOOM EPI, b = 1000 s /mm^2 *******

Multi-transmit = "yes";
Active channels = "both";
Nucleus = "H1";
Coil selection = "SENSE-NV-16";
element selection = "HNACPC";
connection = "d";
Dual coil = "no";
CLEAR = "yes";
body tuned = "no";
FOV RL (mm) = 64;
AP (mm) = 48;
FH (mm) = 60;
Voxel size RL (mm) = 1;
AP (mm) = 1;
Slice thickness (mm) = 5;
Recon voxel size (mm) = 1;
Small FOV imaging = "yes";
OVS = "no";
Reconstruction matrix = 64;
SENSE = "no";
k-t BLAST = "no";
Stacks = 1;
type = "parallel";
slices = 12;
slice gap = "user defined";
gap (mm) = 0;
slice orientation = "transverse";
fold-over direction = "AP";
fat shift direction = "P";
Stack Offc. AP (P=+mm) = -0.332532346;
RL (L=+mm) = 2.41664815;
FH (H=+mm) = 9.80785444e-007;
Ang. AP (deg) = 0;

RL (deg) = -4.24361372;
FH (deg) = 0;
Minimum number of packages = 3;
Slice scan order = "interleaved";
Large table movement = "no";
PlanAlign = "no";
REST slabs = 0;
Shim Size AP (mm) = 54.6583862;
RL (mm) = 56.4545441;
FH (mm) = 80;
Offc. AP (P=+mm) = -0.595884502;
RL (L=+mm) = 2.29769874;
FH (H=+mm) = 0.0195419118;
Ang. AP (deg) = 0;
RL (deg) = -4.24361372;
FH (deg) = 0;
Catheter tracking = "no";
Interactive positioning = "no";
Allow table movement = "no";
Patient position = "head first";
orientation = "supine";
Scan type = "Imaging";
Scan mode = "MS";
technique = "SE";
Modified SE = "no";
Acquisition mode = "cartesian";
Fast Imaging mode = "EPI";
shot mode = "single-shot";
Echoes = 1;
partial echo = "no";
TE = "user defined";
(ms) = 71;
Flip angle (deg) = 90;
TR (beats) = 4;
Halfscan = "yes";
factor = 0.600000024;
Water-fat shift = "minimum";
shared inversion prepulse = "no";
RF Shims = "adaptive";
Shim = "PB-volume";
ShimAlign = "yes";
mDIXON = "no";
Fat suppression = "SPIR";
strength = "strong";
frequency offset = "default";
Water suppression = "no";
Grad. rev. offres. supp. = "no";

```
BB pulse =                    "no";
MTR optimal =                 "no";
MTC =                         "no";
Research prepulse =           "no";
Diffusion mode =              "DTI";
   sequence =                 "SE";
   gradient duration =        "maximum";
   gradient overplus =        "no";
   directional resolution =   "user defined";
      nr of directions = 12;
      dirs in MPS =           "no";
      schemefile =            "H:\schemes\dir10.txt";
   nr of b-factors =          1;
   max b-factor =             1000;
   average high b =           "no";
   interleaved b0 =           "yes";
   allow complex images =     "no";
Elastography mode =           "no";
SAR mode =                    "high";
B1 mode =                     "default";
SAR Patient data =            "auto";
PNS mode =                    "high";
Gradient mode =                        "enhanced";
SofTone mode =                         "no";
Cardiac synchronization =     "trigger";
      device =           "PPU";
Cardiac frequency =           60;
   R-R window (%) =           30, 40;
   Number of heart phases =   "user defined";
      defined phases = 1;
   arrhythmia rejection =     "no";
   no trig period (beats) =   1;
   trigger delay =            "user defined";
      (ms) =                  150;
      adaptive =              "no";
   cycled MS =                "no";
Heart rate > 250 bpm =                 "no";
Slice following =             "no";
REST grid =                   "no";
Respiratory compensation =    "no";
Navigator respiratory comp =  "no";
Flow compensation =           "no";
Temporal slice spacing =      "default";
Package recovery time =                "default";
NSA =                         1;
Manual start =                "no";
Dynamic study =                        "no";
```

dyn stabilization =	"yes";
Arterial Spin labeling =	"no";
Preparation phases =	"auto";
Interactive F0 =	"no";
SmartPlan survey =	"no";
B0 field map =	"no";
B1 field map =	"no";
MIP/MPR =	"no";
Images =	" M", " R", " I", " no";
Autoview image =	" M";
Calculated images =	(4) " no";
Reference tissue =	"White matter";
EPI 2D phase correction =	"no";
Preset window contrast =	"soft";
Reconstruction mode =	"immediate";
Save raw data =	"yes";
Hardcopy protocol =	"no";
Ringing filtering =	"rectangular";
Geometry correction =	"default";
IF_info_seperator =	1634755923;
Total scan duration =	"03:00.3";
Rel. signal level (%) =	100;
Act. TR (ms) =	"4000";
Act. TE (ms) =	"71";
ACQ matrix M x P =	"64 x 47";
ACQ voxel MPS (mm) =	"1.00 / 1.01 / 5.00";
REC voxel MPS (mm) =	"1.00 / 1.00 / 5.00";
Scan percentage (%) =	98.6111145;
Packages =	3;
Min. slice gap (mm) =	0;
Optimal slices =	4;
Diffusion gradient timing DELTA / delta (ms) =	"33.4 / 20.1";
EPI factor =	71;
Entered heartrate =	60;
Trigger delay max. / act. (ms) =	"700.0 / 150.0";
WFS (pix) / BW (Hz) =	"30.973 / 14.0";
BW in EPI freq. dir. (Hz) =	"2100.3";
SAR / local torso =	"< 7 %";
Whole body / level =	"< 0.1 W/kg / normal";
B1 rms =	"0.58 uT / 6 %";
PNS / level =	"91 % / 1st level";
Sound Pressure Level (dB) =	10.035491;

*******   DW ZOOM EPI, b = 2000 s /mm^2 *******

Multi-transmit = "yes";
Active channels = "both";
Nucleus = "H1";
Coil selection = "SENSE-NV-16";
element selection = "HNACPC";
connection = "d";
Dual coil = "no";
CLEAR = "yes";
body tuned = "no";
FOV RL (mm) = 64;
AP (mm) = 48;
FH (mm) = 60;
Voxel size RL (mm) = 1;
AP (mm) = 1;
Slice thickness (mm) = 5;
Recon voxel size (mm) = 1;
Small FOV imaging = "yes";
OVS = "no";
Reconstruction matrix = 64;
SENSE = "no";
k-t BLAST = "no";
Stacks = 1;
type = "parallel";
slices = 12;
slice gap = "user defined";
gap (mm) = 0;
slice orientation = "transverse";
fold-over direction = "AP";
fat shift direction = "P";
Stack Offc. AP (P=+mm) = -0.332532346;
RL (L=+mm) = 2.41664815;
FH (H=+mm) = 9.80785444e-007;
Ang. AP (deg) = 0;
RL (deg) = -4.24361372;
FH (deg) = 0;
Minimum number of packages = 3;
Slice scan order = "interleaved";
Large table movement = "no";
PlanAlign = "no";
REST slabs = 0;
Shim Size AP (mm) = 54.6583862;
RL (mm) = 56.4545441;
FH (mm) = 80;
Offc. AP (P=+mm) = -0.595884502;
RL (L=+mm) = 2.29769874;
FH (H=+mm) = 0.0195419118;
Ang. AP (deg) = 0;

RL (deg) =	-4.24361372;
FH (deg) =	0;
Catheter tracking =	"no";
Interactive positioning =	"no";
Allow table movement =	"no";
Patient position =	"head first";
orientation =	"supine";
Scan type =	"Imaging";
Scan mode =	"MS";
technique =	"SE";
Modified SE =	"no";
Acquisition mode =	"cartesian";
Fast Imaging mode =	"EPI";
shot mode =	"single-shot";
Echoes =	1;
partial echo =	"no";
TE =	"user defined";
(ms) =	71;
Flip angle (deg) =	90;
TR (beats) =	4;
Halfscan =	"yes";
factor =	0.600000024;
Water-fat shift =	"minimum";
shared inversion prepulse =	"no";
RF Shims =	"adaptive";
Shim =	"PB-volume";
ShimAlign =	"yes";
mDIXON =	"no";
Fat suppression =	"SPIR";
strength =	"strong";
frequency offset =	"default";
Water suppression =	"no";
Grad. rev. offres. supp. =	"no";
BB pulse =	"no";
MTR optimal =	"no";
MTC =	"no";
Research prepulse =	"no";
Diffusion mode =	"DTI";
sequence =	"SE";
gradient duration =	"maximum";
gradient overplus =	"no";
directional resolution =	"user defined";
nr of directions = 20;	
dirs in MPS =	"no";
schemefile =	"H:\schemes\dir18.txt";
nr of b-factors =	1;
max b-factor =	2000;

average high b = "no";
interleaved b0 = "yes";
allow complex images = "no";
Elastography mode = "no";
SAR mode = "high";
B1 mode = "default";
SAR Patient data = "auto";
PNS mode = "high";
Gradient mode = "enhanced";
SofTone mode = "no";
Cardiac synchronization = "trigger";
device = "PPU";
Cardiac frequency = 60;
R-R window (%) = 30, 40;
Number of heart phases = "user defined";
defined phases = 1;
arrhythmia rejection = "no";
no trig period (beats) = 1;
trigger delay = "user defined";
(ms) = 150;
adaptive = "no";
cycled MS = "no";
Heart rate > 250 bpm = "no";
Slice following = "no";
REST grid = "no";
Respiratory compensation = "no";
Navigator respiratory comp = "no";
Flow compensation = "no";
Temporal slice spacing = "default";
Package recovery time = "default";
NSA = 1;
Manual start = "no";
Dynamic study = "no";
dyn stabilization = "yes";
Arterial Spin labeling = "no";
Preparation phases = "auto";
Interactive F0 = "no";
SmartPlan survey = "no";
B0 field map = "no";
B1 field map = "no";
MIP/MPR = "no";
Images = " M", " R", " I", " no";
Autoview image = " M";
Calculated images = (4) " no";
Reference tissue = "White matter";
EPI 2D phase correction = "no";
Preset window contrast = "soft";

Reconstruction mode = "immediate";
Save raw data = "yes";
Hardcopy protocol = "no";
Ringing filtering = "rectangular";
Geometry correction = "default";
IF_info_seperator = 1634755923;
Total scan duration = "04:52.5";
Rel. signal level (%) = 100;
Act. TR (ms) = "4000";
Act. TE (ms) = "71";
ACQ matrix M x P = "64 x 47";
ACQ voxel MPS (mm) = "1.00 / 1.01 / 5.00";
REC voxel MPS (mm) = "1.00 / 1.00 / 5.00";
Scan percentage (%) = 98.6111145;
Packages = 3;
Min. slice gap (mm) = 0;
Optimal slices = 4;
Diffusion gradient timing DELTA / delta (ms) =
"33.4 / 20.1";
EPI factor = 71;
Entered heartrate = 60;
Trigger delay max. / act. (ms) = "700.0 / 150.0";
WFS (pix) / BW (Hz) = "30.973 / 14.0";
BW in EPI freq. dir. (Hz) = "2100.3";
SAR / local torso = "< 7 %";
Whole body / level = "< 0.1 W/kg / normal";
B1 rms = "0.58 uT / 6 %";
PNS / level = "91 % / 1st level";
Sound Pressure Level (dB) = 10.035491;

******* DW ZOOM EPI, b = 2800 s /mm^2 *******

Multi-transmit = "yes";
Active channels = "both";
Nucleus = "H1";
Coil selection = "SENSE-NV-16";
element selection = "HNACPC";
connection = "d";
Dual coil = "no";
CLEAR = "yes";
body tuned = "no";
FOV RL (mm) = 64;
AP (mm) = 48;
FH (mm) = 60;
Voxel size RL (mm) = 1;
AP (mm) = 1;

Slice thickness (mm) = 5;
Recon voxel size (mm) = 1;
Small FOV imaging = "yes";
OVS = "no";
Reconstruction matrix = 64;
SENSE = "no";
k-t BLAST = "no";
Stacks = 1;
type = "parallel";
slices = 12;
slice gap = "user defined";
gap (mm) = 0;
slice orientation = "transverse";
fold-over direction = "AP";
fat shift direction = "P";
Stack Offc. AP (P=+mm) = -0.332532346;
RL (L=+mm) = 2.41664815;
FH (H=+mm) = 9.80785444e-007;
Ang. AP (deg) = 0;
RL (deg) = -4.24361372;
FH (deg) = 0;
Minimum number of packages = 3;
Slice scan order = "interleaved";
Large table movement = "no";
PlanAlign = "no";
REST slabs = 0;
Shim Size AP (mm) = 54.6583862;
RL (mm) = 56.4545441;
FH (mm) = 80;
Offc. AP (P=+mm) = -0.595884502;
RL (L=+mm) = 2.29769874;
FH (H=+mm) = 0.0195419118;
Ang. AP (deg) = 0;
RL (deg) = -4.24361372;
FH (deg) = 0;
Catheter tracking = "no";
Interactive positioning = "no";
Allow table movement = "no";
Patient position = "head first";
orientation = "supine";
Scan type = "Imaging";
Scan mode = "MS";
technique = "SE";
Modified SE = "no";
Acquisition mode = "cartesian";
Fast Imaging mode = "EPI";
shot mode = "single-shot";

Echoes =	1;
partial echo =	"no";
TE =	"user defined";
(ms) =	71;
Flip angle (deg) =	90;
TR (beats) =	4;
Halfscan =	"yes";
factor =	0.600000024;
Water-fat shift =	"minimum";
shared inversion prepulse =	"no";
RF Shims =	"adaptive";
Shim =	"PB-volume";
ShimAlign =	"yes";
mDIXON =	"no";
Fat suppression =	"SPIR";
strength =	"strong";
frequency offset =	"default";
Water suppression =	"no";
Grad. rev. offres. supp. =	"no";
BB pulse =	"no";
MTR optimal =	"no";
MTC =	"no";
Research prepulse =	"no";
Diffusion mode =	"DTI";
sequence =	"SE";
gradient duration =	"maximum";
gradient overplus =	"no";
directional resolution =	"user defined";
nr of directions = 30;	
dirs in MPS =	"no";
schemefile =	"H:\schemes\dir28.txt";
nr of b-factors =	1;
max b-factor =	2800;
average high b =	"no";
interleaved b0 =	"yes";
allow complex images =	"no";
Elastography mode =	"no";
SAR mode =	"high";
B1 mode =	"default";
SAR Patient data =	"auto";
PNS mode =	"high";
Gradient mode =	"enhanced";
SofTone mode =	"no";
Cardiac synchronization =	"trigger";
device = "PPU";	
Cardiac frequency =	60;
R-R window (%) =	30, 40;

Number of heart phases = "user defined";
defined phases = 1;
arrhythmia rejection = "no";
no trig period (beats) = 1;
trigger delay = "user defined";
(ms) = 150;
adaptive = "no";
cycled MS = "no";
Heart rate > 250 bpm = "no";
Slice following = "no";
REST grid = "no";
Respiratory compensation = "no";
Navigator respiratory comp = "no";
Flow compensation = "no";
Temporal slice spacing = "default";
Package recovery time = "default";
NSA = 1;
Manual start = "no";
Dynamic study = "no";
dyn stabilization = "yes";
Arterial Spin labeling = "no";
Preparation phases = "auto";
Interactive F0 = "no";
SmartPlan survey = "no";
B0 field map = "no";
B1 field map = "no";
MIP/MPR = "no";
Images = " M", " R", " I", " no";
Autoview image = " M";
Calculated images = (4) " no";
Reference tissue = "White matter";
EPI 2D phase correction = "no";
Preset window contrast = "soft";
Reconstruction mode = "immediate";
Save raw data = "yes";
Hardcopy protocol = "no";
Ringing filtering = "rectangular";
Geometry correction = "default";
IF_info_seperator = 1634755923;
Total scan duration = "07:12.8";
Rel. signal level (%) = 100;
Act. TR (ms) = "4000";
Act. TE (ms) = "71";
ACQ matrix M x P = "64 x 47";
ACQ voxel MPS (mm) = "1.00 / 1.01 / 5.00";
REC voxel MPS (mm) = "1.00 / 1.00 / 5.00";
Scan percentage (%) = 98.6111145;

Packages = 3;
Min. slice gap (mm) = 0;
Optimal slices = 4;
Diffusion gradient timing DELTA / delta (ms) =
"33.4 / 20.1";
EPI factor = 71;
Entered heartrate = 60;
Trigger delay max. / act. (ms) = "700.0 / 150.0";
WFS (pix) / BW (Hz) = "30.973 / 14.0";
BW in EPI freq. dir. (Hz) = "2100.3";
SAR / local torso = "< 7 %";
Whole body / level = "< 0.1 W/kg / normal";
B1 rms = "0.58 uT / 6 %";
PNS / level = "91 % / 1st level";
Sound Pressure Level (dB) = 10.035491;

******* DW ZOOM EPI, b = 300 s /mm^2 *******

Multi-transmit = "yes";
Active channels = "both";
Nucleus = "H1";
Coil selection = "SENSE-NV-16";
element selection = "HNACPC";
connection = "d";
Dual coil = "no";
CLEAR = "yes";
body tuned = "no";
FOV RL (mm) = 64;
AP (mm) = 48;
FH (mm) = 60;
Voxel size RL (mm) = 1;
AP (mm) = 1;
Slice thickness (mm) = 5;
Recon voxel size (mm) = 1;
Small FOV imaging = "yes";
OVS = "no";
Reconstruction matrix = 64;
SENSE = "no";
k-t BLAST = "no";
Stacks = 1;
type = "parallel";
slices = 12;
slice gap = "user defined";
gap (mm) = 0;
slice orientation = "transverse";
fold-over direction = "AP";

fat shift direction = "P";
Stack Offc. AP (P=+mm) = -0.332532346;
RL (L=+mm) = 2.41664815;
FH (H=+mm) = 9.80785444e-007;
Ang. AP (deg) = 0;
RL (deg) = -4.24361372;
FH (deg) = 0;
Minimum number of packages = 3;
Slice scan order = "interleaved";
Large table movement = "no";
PlanAlign = "no";
REST slabs = 0;
Shim Size AP (mm) = 54.6583862;
RL (mm) = 56.4545441;
FH (mm) = 80;
Offc. AP (P=+mm) = -0.595884502;
RL (L=+mm) = 2.29769874;
FH (H=+mm) = 0.0195419118;
Ang. AP (deg) = 0;
RL (deg) = -4.24361372;
FH (deg) = 0;
Catheter tracking = "no";
Interactive positioning = "no";
Allow table movement = "no";
Patient position = "head first";
orientation = "supine";
Scan type = "Imaging";
Scan mode = "MS";
technique = "SE";
Modified SE = "no";
Acquisition mode = "cartesian";
Fast Imaging mode = "EPI";
shot mode = "single-shot";
Echoes = 1;
partial echo = "no";
TE = "user defined";
(ms) = 71;
Flip angle (deg) = 90;
TR (beats) = 4;
Halfscan = "yes";
factor = 0.600000024;
Water-fat shift = "minimum";
shared inversion prepulse = "no";
RF Shims = "adaptive";
Shim = "PB-volume";
ShimAlign = "yes";
mDIXON = "no";

Fat suppression = "SPIR";
  strength = "strong";
  frequency offset = "default";
Water suppression = "no";
Grad. rev. offres. supp. = "no";
BB pulse = "no";
MTR optimal = "no";
MTC = "no";
Research prepulse = "no";
Diffusion mode = "DTI";
  sequence = "SE";
  gradient duration = "maximum";
  gradient overplus = "no";
  directional resolution = "user defined";
    nr of directions = 6;
    dirs in MPS = "no";
    schemefile = "H:\schemes\dir4.txt";
  nr of b-factors = 1;
  max b-factor = 300;
  average high b = "no";
  interleaved b0 = "yes";
  allow complex images = "no";
Elastography mode = "no";
SAR mode = "high";
B1 mode = "default";
SAR Patient data = "auto";
PNS mode = "high";
Gradient mode = "enhanced";
SofTone mode = "no";
Cardiac synchronization = "trigger";
    device = "PPU";
Cardiac frequency = 60;
  R-R window (%) = 30, 40;
  Number of heart phases = "user defined";
    defined phases = 1;
  arrhythmia rejection = "no";
  no trig period (beats) = 1;
  trigger delay = "user defined";
    (ms) = 150;
    adaptive = "no";
  cycled MS = "no";
Heart rate > 250 bpm = "no";
Slice following = "no";
REST grid = "no";
Respiratory compensation = "no";
Navigator respiratory comp = "no";
Flow compensation = "no";

Temporal slice spacing = "default";
Package recovery time = "default";
NSA = 1;
Manual start = "no";
Dynamic study = "no";
  dyn stabilization = "yes";
Arterial Spin labeling = "no";
Preparation phases = "auto";
Interactive F0 = "no";
SmartPlan survey = "no";
B0 field map = "no";
B1 field map = "no";
MIP/MPR = "no";
Images = " M", " R", " I", " no";
Autoview image = " M";
Calculated images = (4) " no";
Reference tissue = "White matter";
EPI 2D phase correction = "no";
Preset window contrast = "soft";
Reconstruction mode = "immediate";
Save raw data = "yes";
Hardcopy protocol = "no";
Ringing filtering = "rectangular";
Geometry correction = "default";
IF_info_seperator = 1634755923;
Total scan duration = "01:36.2";
Rel. signal level (%) = 100;
Act. TR (ms) = "4000";
Act. TE (ms) = "71";
ACQ matrix M x P = "64 x 47";
ACQ voxel MPS (mm) = "1.00 / 1.01 / 5.00";
REC voxel MPS (mm) = "1.00 / 1.00 / 5.00";
Scan percentage (%) = 98.6111145;
Packages = 3;
Min. slice gap (mm) = 0;
Optimal slices = 4;
Diffusion gradient timing DELTA / delta (ms) ="33.4 / 20.1";
EPI factor = 71;
Entered heartrate = 60;
Trigger delay max. / act. (ms) = "700.0 / 150.0";
WFS (pix) / BW (Hz) = "30.973 / 14.0";
BW in EPI freq. dir. (Hz) = "2100.3";
SAR / local torso = "< 7 %";
Whole body / level = "< 0.1 W/kg / normal";
B1 rms = "0.58 uT / 6 %";
PNS / level = "91 % / 1st level";
Sound Pressure Level (dB) = 10.035491;

*******   Quantitative Magnetisation Transfer ZOOM EPI   *******

Multi-transmit = "yes";
Active channels = "both";
Nucleus = "H1";
Coil selection = "SENSE-NV-16";
element selection = "HNACPC";
connection = "d";
Dual coil = "no";
CLEAR = "yes";
body tuned = "no";
FOV RL (mm) = 64;
AP (mm) = 48;
FH (mm) = 60;
Voxel size RL (mm) = 1;
AP (mm) = 1;
Slice thickness (mm) = 5;
Recon voxel size (mm) = 1;
Small FOV imaging = "yes";
OVS = "no";
Reconstruction matrix = 64;
SENSE = "no";
k-t BLAST = "no";
Stacks = 1;
type = "parallel";
slices = 12;
slice gap = "user defined";
gap (mm) = 0;
slice orientation = "transverse";
fold-over direction = "AP";
fat shift direction = "P";
Stack Offc. AP (P=+mm) = -0.332532346;
RL (L=+mm) = 2.41664815;
FH (H=+mm) = 9.80785444e-007;
Ang. AP (deg) = 0;
RL (deg) = -4.24361372;
FH (deg) = 0;
Minimum number of packages = 3;
Slice scan order = "interleaved";
Large table movement = "no";
PlanAlign = "no";
REST slabs = 0;
Shim Size AP (mm) = 54.6583862;
RL (mm) = 56.4545441;
FH (mm) = 80;
Offc. AP (P=+mm) = -0.595884502;

RL (L=+mm) = 2.29769874;
FH (H=+mm) = 0.0195419118;
Ang. AP (deg) = 0;
RL (deg) = -4.24361372;
FH (deg) = 0;
Catheter tracking = "no";
Interactive positioning = "no";
Allow table movement = "no";
Patient position = "head first";
orientation = "supine";
Scan type = "Imaging";
Scan mode = "MS";
technique = "SE";
Modified SE = "no";
Acquisition mode = "cartesian";
Fast Imaging mode = "EPI";
shot mode = "single-shot";
Echoes = 1;
partial echo = "no";
TE = "shortest";
Flip angle (deg) = 90;
TR = "shortest";
Halfscan = "yes";
factor = 0.600000024;
Water-fat shift = "minimum";
shared inversion prepulse = "no";
RF Shims = "adaptive";
Shim = "PB-volume";
ShimAlign = "yes";
mDIXON = "no";
Fat suppression = "SPIR";
strength = "strong";
frequency offset = "default";
Water suppression = "no";
Grad. rev. offres. supp. = "yes";
BB pulse = "no";
MTR optimal = "no";
MTC = "no";
Research prepulse = "yes";
pulse shape = "sg_100_100_0";
Shared per package = "yes";
Slice shuffling = "dynamic loop";
elements = 25;
all same = "yes";
repeated pulse angle = 1471;
repeated pulse dur = 15;
repeated pulse phase = 0;

repeated pulse interval = 15;
delay time = 0;
freq offset = 0;
select grad in (uT/m) = 0;
Diffusion mode = "no";
Elastography mode = "no";
SAR mode = "high";
B1 mode = "default";
SAR Patient data = "auto";
PNS mode = "high";
Gradient mode = "enhanced";
SofTone mode = "no";
Cardiac synchronization = "no";
Heart rate > 250 bpm = "no";
Respiratory compensation = "no";
Navigator respiratory comp = "no";
Flow compensation = "yes";
Temporal slice spacing = "minimal";
Package recovery time = "user defined";
Recovery time = 6260;
NSA = 1;
Manual start = "no";
Dynamic study = "individual";
dyn scans = 44;
recon multiplier = 1;
dyn scan times = "shortest";
FOV time mode = "default";
dummy scans = 0;
immediate subtraction = "no";
fast next scan = "no";
synch. ext. device = "no";
Noise scan = "no";
Research Prepulse = "yes";
REPP Freq Series = "no";
Manual frequency offests = "yes";
Frequency Offsets = (4) 96000,
(4) 8393, (4) 13597, (4) 1070,
(4) 14125, (4) 1007, (4) 1013,
(4) 2698, (4) 3763, (4) 1053,
(4) 3779, (20) 0;
Manual flip angles = "yes";
Flip angles = (4) 300, (4) 1465,
(4) 1440, (4) 426, (4) 1429,
(4) 433, (4) 1460, (4) 524,
(4) 1462, (4) 1459, (4) 1438,
(20) 0;
Manual train length = "no";

dyn stabilization = "no";
prospect. motion corr. = "no";
Keyhole = "no";
Arterial Spin labeling = "no";
Preparation phases = "auto";
Interactive F0 = "no";
SmartPlan survey = "no";
B0 field map = "no";
B1 field map = "no";
MIP/MPR = "no";
Images = " M", " R", " I", " no";
Autoview image = " M";
Calculated images = (4) " no";
Reference tissue = "Grey matter";
EPI 2D phase correction = "no";
Preset window contrast = "soft";
Reconstruction mode = "real time";
reuse memory = "no";
Save raw data = "yes";
Hardcopy protocol = "no";
Ringing filtering = "rectangular";
Geometry correction = "default";
IF_info_seperator = 1634755923;
Total scan duration = "16:18.3";
Rel. signal level (%) = 100;
Act. TR (ms) = "7246";
Act. TE (ms) = "24";
Dyn. scan time = "00:21.7";
ACQ matrix M x P = "64 x 47";
ACQ voxel MPS (mm) = "1.00 / 1.01 / 5.00";
REC voxel MPS (mm) = "1.00 / 1.00 / 5.00";
Scan percentage (%) = 98.6111145;
Packages = 3;
Min. slice gap (mm) = 0;
EPI factor = 71;
WFS (pix) / BW (Hz) = "30.973 / 14.0";
BW in EPI freq. dir. (Hz) = "2100.3";
Full flow comp. = "no";
SAR / local torso = "< 64 %";
Whole body / level = "< 1.0 W/kg / normal";
B1 rms = "1.75 uT / 56 %";
PNS / level = "91 % / 1st level";
Sound Pressure Level (dB) = 15.4398813;

Vendor 2 (3T Siemens Prisma)

***** DWI b = 2800 s/mm^2 *****

Properties

Prio recon	Off
Load images to viewer	On
Inline movie	Off
Auto store images	On
Load images to stamp segments	Off
Load images to graphic segments	Off
Auto open inline display	Off
Auto close inline display	Off
Start measurement without further preparation	Off
Wait for user to start	Off
Start measurements	Single measurement

Routine

Slice group	1
Slices	12
Dist. factor	0 %
Position	L2.2 P5.2 H52.0 mm
Orientation	T > C5.2
Phase enc. dir.	A >> P
AutoAlign	---
Phase oversampling	0 %
FoV read	64 mm
FoV phase	78.1 %
Slice thickness	5.0 mm
TR	150 ms
TE	85.0 ms
Averages	1
Concatenations	12
Filter	Raw filter, Prescan Normalize
Coil elements	HC7;NC1,2

Contrast - Common

TR	150 ms
TE	85.0 ms
MTC	Off
Magn. preparation	None
Fat suppr.	Fat sat.
Fat sat. mode	Strong

Contrast - Dynamic

Averages	1
Averaging mode	Long term
Reconstruction	Magnitude
Measurements	1
Delay in TR	0 ms
Multiple series	Off

Resolution - Common

FoV read	64 mm
FoV phase	78.1 %
Slice thickness	5.0 mm
Base resolution	64
Phase resolution	100 %
Phase partial Fourier	6/8
Interpolation	Off

Resolution - iPAT

Accel. mode	None

Resolution - Filter Image

Distortion Corr.	Off
Prescan Normalize	On
Dynamic Field Corr.	Off

Resolution - Filter Rawdata

Raw filter	On
Elliptical filter	Off

Geometry - Common

Slice group	1
Slices	12
Dist. factor	0 %
Position	L2.2 P5.2 H52.0 mm
Orientation	T > C5.2
Phase enc. dir.	A >> P
FoV read	64 mm
FoV phase	78.1 %
Slice thickness	5.0 mm
TR	150 ms
Multi-slice mode	Interleaved
Series	Interleaved
Concatenations	12

Geometry - AutoAlign

Slice group	1
Position	L2.2 P5.2 H52.0 mm
Orientation	T > C5.2
Phase enc. dir.	A >> P
AutoAlign	---
Initial Position	L2.2 P5.2 H38.0
Phase	8.6 mm
Read	-2.2 mm
Shift	37.4 mm
Initial Rotation	0.00 deg
Initial Orientation	T > C
T > C	5.2
> S	0.0

Geometry - Saturation

Fat suppr.	Fat sat.
Fat sat. mode	Strong
Special sat.	None

Geometry - Navigator

Geometry - Tim Planning Suite

Set-n-Go Protocol	Off
Table position	H
Table position	14 mm
Inline Composing	Off

System - Miscellaneous

Positioning mode	REF
Table position	H
Table position	14 mm
MSMA	S - C - T
Sagittal	L >> R
Coronal	A >> P
Transversal	H >> F
Coil Combine Mode	Adaptive Combine

System - Miscellaneous

Matrix Optimization	Off
AutoAlign	---
Coil Select Mode	Off - AutoCoilSelect

System - Adjustments

B0 Shim mode	Standard
B1 Shim mode	TrueForm
Adjust with body coil	Off
Confirm freq. adjustment	Off
Assume Dominant Fat	Off
Assume Silicone	Off
Adjustment Tolerance	Auto

System - Adjust Volume

Position	L2.2 P5.2 H52.0 mm
Orientation	T > C5.2
Rotation	0.00 deg
A >> P	50 mm
R >> L	64 mm
F >> H	60 mm
Reset	Off
Couple to	Acquisition volume

System - pTx Volumes

B1 Shim mode	TrueForm
Excitation	ZOOMit
pTx Volume	1
Vol. Property	Optimization Vol.
Position	L2.2 P5.2 H52.0 mm
Orientation	T > C5.2
Rotation	0 deg
A >> P	250 mm
R >> L	64 mm
F >> H	60 mm
Vol. Visibility	On

System - Tx/Rx

Frequency 1H	123.256882 MHz
Correction factor	1
Gain	High
Img. Scale Cor.	1.000
Reset	Off
? Ref. amplitude 1H	0.000 V

Physio - Signal1

1st Signal/Mode	Pulse/Trigger
Average cycle	973 ± 45 ms
Average cycle	996 ± 45 ms
Acquisition window	650 ms
Trigger pulse	1
Trigger delay	200 ms
TR	150 ms
Concatenations	12
Phases	1

Physio - PACE

Resp. control	Off
Concatenations	12

Diff - Neuro

Diffusion mode	MDDW
Diff. directions	30
Diffusion Scheme	Monopolar

Diff - Neuro

Diff. weightings	2
b-value 1	0 s/mm²
b-value 2	2800 s/mm²
b-value 1	1
b-value 2	1
Diff. weighted images	On
Trace weighted images	On
ADC maps	Off
FA maps	Off
Mosaic	On
Tensor	Off
Noise level	0

Diff - Body

Diffusion mode	MDDW
Diff. directions	30
Diffusion Scheme	Monopolar
Diff. weightings	2
b-value 1	0 s/mm²
b-value 2	2800 s/mm²
b-value 1	1
b-value 2	1
Diff. weighted images	On
Trace weighted images	On
ADC maps	Off
Exponential ADC Maps	Off
FA maps	Off
Invert Gray Scale	Off
Calculated Image	Off
b-Value >=	0 s/mm²
Noise level	0

Diff - Composing

Inline Composing	Off
Distortion Corr.	Off

Sequence - Part 1

Introduction	On
Optimization	None
Multi-slice mode	Interleaved
Free echo spacing	Off
Echo spacing	0.93 ms
Bandwidth	1240 Hz/Px

Sequence - Part 2

EPI factor	50
RF pulse type	Normal
Gradient mode	Performance
Excitation	ZOOMit

Sequence - pTX Pulses

pTX Pulse	1
TX acceleration	1.0

***** DWI b = 2000 s/mm^2 *****

Properties

Prio recon	Off
Load images to viewer	On
Inline movie	Off
Auto store images	On
Load images to stamp segments	Off
Load images to graphic segments	Off
Auto open inline display	Off
Auto close inline display	Off
Start measurement without further preparation	Off
Wait for user to start	Off
Start measurements	Single measurement

Routine

Slice group	1
Slices	12
Dist. factor	0 %
Position	L2.2 P5.2 H52.0 mm
Orientation	T > C5.2
Phase enc. dir.	A >> P
AutoAlign	---
Phase oversampling	0 %
FoV read	64 mm
FoV phase	78.1 %
Slice thickness	5.0 mm
TR	150 ms
TE	85.0 ms
Averages	1
Concatenations	12
Filter	Raw filter, Prescan Normalize
Coil elements	HC7;NC1,2

Contrast - Common

TR	150 ms
TE	85.0 ms
MTC	Off
Magn. preparation	None
Fat suppr.	Fat sat.
Fat sat. mode	Strong

Contrast - Dynamic

Averages	1
Averaging mode	Long term
Reconstruction	Magnitude
Measurements	1
Delay in TR	0 ms
Multiple series	Off

Resolution - Common

FoV read	64 mm
FoV phase	78.1 %
Slice thickness	5.0 mm
Base resolution	64
Phase resolution	100 %
Phase partial Fourier	6/8
Interpolation	Off

Resolution - iPAT

Accel. mode	None

Resolution - Filter Image

Distortion Corr.	Off
Prescan Normalize	On
Dynamic Field Corr.	Off

Resolution - Filter Rawdata

Raw filter	On
Elliptical filter	Off

Geometry - Common

Slice group	1
Slices	12
Dist. factor	0 %
Position	L2.2 P5.2 H52.0 mm
Orientation	T > C5.2
Phase enc. dir.	A >> P
FoV read	64 mm
FoV phase	78.1 %
Slice thickness	5.0 mm
TR	150 ms
Multi-slice mode	Interleaved
Series	Interleaved
Concatenations	12

Geometry - AutoAlign

Slice group	1
Position	L2.2 P5.2 H52.0 mm
Orientation	T > C5.2
Phase enc. dir.	A >> P
AutoAlign	---
Initial Position	L2.2 P5.2 H38.0
Phase	8.6 mm
Read	-2.2 mm
Shift	37.4 mm
Initial Rotation	0.00 deg
Initial Orientation	T > C
T > C	5.2
> S	0.0

Geometry - Saturation

Fat suppr.	Fat sat.
Fat sat. mode	Strong
Special sat.	None

Geometry - Navigator

Geometry - Tim Planning Suite

Set-n-Go Protocol	Off
Table position	H
Table position	14 mm
Inline Composing	Off

System - Miscellaneous

Positioning mode	REF
Table position	H
Table position	14 mm
MSMA	S - C - T
Sagittal	L >> R
Coronal	A >> P
Transversal	H >> F
Coil Combine Mode	Adaptive Combine

System - Miscellaneous

Matrix Optimization	Off
AutoAlign	---
Coil Select Mode	Off - AutoCoilSelect

System - Adjustments

B0 Shim mode	Standard
B1 Shim mode	TrueForm
Adjust with body coil	Off
Confirm freq. adjustment	Off
Assume Dominant Fat	Off
Assume Silicone	Off
Adjustment Tolerance	Auto

System - Adjust Volume

Position	L2.2 P5.2 H52.0 mm
Orientation	T > C5.2
Rotation	0.00 deg
A >> P	50 mm
R >> L	64 mm
F >> H	60 mm
Reset	Off
Couple to	Acquisition volume

System - pTx Volumes

B1 Shim mode	TrueForm
Excitation	ZOOMit
pTx Volume	1
Vol. Property	Optimization Vol.
Position	L2.2 P5.2 H52.0 mm
Orientation	T > C5.2
Rotation	0 deg
A >> P	250 mm
R >> L	64 mm
F >> H	60 mm
Vol. Visibility	On

System - Tx/Rx

Frequency 1H	123.256882 MHz
Correction factor	1
Gain	High
Img. Scale Cor.	1.000
Reset	Off
? Ref. amplitude 1H	0.000 V

Physio - Signal1

1st Signal/Mode	Pulse/Trigger
Average cycle	975 ± 43 ms
Average cycle	993 ± 43 ms
Acquisition window	650 ms
Trigger pulse	1
Trigger delay	200 ms
TR	150 ms
Concatenations	12
Phases	1

Physio - PACE

Resp. control	Off
Concatenations	12

Diff - Neuro

Diffusion mode	MDDW
Diff. directions	20
Diffusion Scheme	Monopolar

Diff - Neuro

Diff. weightings	2
b-value 1	0 s/mm²
b-value 2	2000 s/mm²
b-value 1	1
b-value 2	1
Diff. weighted images	On
Trace weighted images	On
ADC maps	Off
FA maps	Off
Mosaic	On
Tensor	Off
Noise level	0

Diff - Body

Diffusion mode	MDDW
Diff. directions	20
Diffusion Scheme	Monopolar
Diff. weightings	2
b-value 1	0 s/mm²
b-value 2	2000 s/mm²
b-value 1	1
b-value 2	1
Diff. weighted images	On
Trace weighted images	On
ADC maps	Off
Exponential ADC Maps	Off
FA maps	Off
Invert Gray Scale	Off
Calculated Image	Off
b-Value >=	0 s/mm²
Noise level	0

Diff - Composing

Inline Composing	Off
Distortion Corr.	Off

Sequence - Part 1

Introduction	On
Optimization	None
Multi-slice mode	Interleaved
Free echo spacing	Off
Echo spacing	0.93 ms
Bandwidth	1240 Hz/Px

Sequence - Part 2

EPI factor	50
RF pulse type	Normal
Gradient mode	Performance
Excitation	ZOOMit

Sequence - pTX Pulses

pTX Pulse	1
TX acceleration	1.0

***** DWI b = 1000 s/mm² *****

Properties

Prio recon	Off
Load images to viewer	On
Inline movie	Off
Auto store images	On
Load images to stamp segments	Off
Load images to graphic segments	Off
Auto open inline display	Off
Auto close inline display	Off
Start measurement without further preparation	Off
Wait for user to start	Off
Start measurements	Single measurement

Routine

Slice group	1
Slices	12
Dist. factor	0 %
Position	L2.2 P5.2 H52.0 mm
Orientation	T > C5.2
Phase enc. dir.	A >> P
AutoAlign	---
Phase oversampling	0 %
FoV read	64 mm
FoV phase	78.1 %
Slice thickness	5.0 mm
TR	150 ms
TE	85.0 ms
Averages	1
Concatenations	12
Filter	Raw filter, Prescan Normalize
Coil elements	HC7;NC1,2

Contrast - Common

TR	150 ms
TE	85.0 ms
MTC	Off
Magn. preparation	None
Fat suppr.	Fat sat.
Fat sat. mode	Strong

Contrast - Dynamic

Averages	1
Averaging mode	Long term
Reconstruction	Magnitude
Measurements	1
Delay in TR	0 ms
Multiple series	Off

Resolution - Common

FoV read	64 mm
FoV phase	78.1 %
Slice thickness	5.0 mm
Base resolution	64
Phase resolution	100 %
Phase partial Fourier	6/8
Interpolation	Off

Resolution - iPAT

Accel. mode	None

Resolution - Filter Image

Distortion Corr.	Off
Prescan Normalize	On
Dynamic Field Corr.	Off

Resolution - Filter Rawdata

Raw filter	On
Elliptical filter	Off

Geometry - Common

Slice group	1
Slices	12
Dist. factor	0 %
Position	L2.2 P5.2 H52.0 mm
Orientation	T > C5.2
Phase enc. dir.	A >> P
FoV read	64 mm
FoV phase	78.1 %
Slice thickness	5.0 mm
TR	150 ms
Multi-slice mode	Interleaved
Series	Interleaved
Concatenations	12

Geometry - AutoAlign

Slice group	1
Position	L2.2 P5.2 H52.0 mm
Orientation	T > C5.2
Phase enc. dir.	A >> P
AutoAlign	---
Initial Position	L2.2 P5.2 H38.0
Phase	8.6 mm
Read	-2.2 mm
Shift	37.4 mm
Initial Rotation	0.00 deg
Initial Orientation	T > C
T > C	5.2
> S	0.0

Geometry - Saturation

Fat suppr.	Fat sat.
Fat sat. mode	Strong
Special sat.	None

Geometry - Navigator

Geometry - Tim Planning Suite

Set-n-Go Protocol	Off
Table position	H
Table position	14 mm
Inline Composing	Off

System - Miscellaneous

Positioning mode	REF
Table position	H
Table position	14 mm
MSMA	S - C - T
Sagittal	L >> R
Coronal	A >> P
Transversal	H >> F
Coil Combine Mode	Adaptive Combine

System - Miscellaneous

Matrix Optimization	Off
AutoAlign	---
Coil Select Mode	Off - AutoCoilSelect

System - Adjustments

B0 Shim mode	Standard
B1 Shim mode	TrueForm
Adjust with body coil	Off
Confirm freq. adjustment	Off
Assume Dominant Fat	Off
Assume Silicone	Off
Adjustment Tolerance	Auto

System - Adjust Volume

Position	L2.2 P5.2 H52.0 mm
Orientation	T > C5.2
Rotation	0.00 deg
A >> P	50 mm
R >> L	64 mm
F >> H	60 mm
Reset	Off
Couple to	Acquisition volume

System - pTx Volumes

B1 Shim mode	TrueForm
Excitation	ZOOMit
pTx Volume	1
Vol. Property	Optimization Vol.
Position	L2.2 P5.2 H52.0 mm
Orientation	T > C5.2
Rotation	0 deg
A >> P	250 mm
R >> L	64 mm
F >> H	60 mm
Vol. Visibility	On

System - Tx/Rx

Frequency 1H	123.256882 MHz
Correction factor	1
Gain	High
Img. Scale Cor.	1.000
Reset	Off
? Ref. amplitude 1H	0.000 V

Physio - Signal1

1st Signal/Mode	Pulse/Trigger
Average cycle	974 ± 41 ms
Average cycle	971 ± 41 ms
Acquisition window	650 ms
Trigger pulse	1
Trigger delay	200 ms
TR	150 ms
Concatenations	12
Phases	1

Physio - PACE

Resp. control	Off
Concatenations	12

Diff - Neuro

Diffusion mode	MDDW
Diff. directions	12
Diffusion Scheme	Monopolar

Diff - Neuro

Diff. weightings	2
b-value 1	0 s/mm²
b-value 2	1000 s/mm²
b-value 1	1
b-value 2	1
Diff. weighted images	On
Trace weighted images	On
ADC maps	Off
FA maps	Off
Mosaic	On
Tensor	Off
Noise level	0

Diff - Body

Diffusion mode	MDDW
Diff. directions	12
Diffusion Scheme	Monopolar
Diff. weightings	2
b-value 1	0 s/mm²
b-value 2	1000 s/mm²
b-value 1	1
b-value 2	1
Diff. weighted images	On
Trace weighted images	On
ADC maps	Off
Exponential ADC Maps	Off
FA maps	Off
Invert Gray Scale	Off
Calculated Image	Off
b-Value >=	0 s/mm²
Noise level	0

Diff - Composing

Inline Composing	Off
Distortion Corr.	Off

Sequence - Part 1

Introduction	On
Optimization	None
Multi-slice mode	Interleaved
Free echo spacing	Off
Echo spacing	0.93 ms
Bandwidth	1240 Hz/Px

Sequence - Part 2

EPI factor	50
RF pulse type	Normal
Gradient mode	Performance
Excitation	ZOOMit

Sequence - pTX Pulses

pTX Pulse	1
TX acceleration	1.0

***** DWI b = 300 s/mm^2 *****

Properties

Prio recon	Off
Load images to viewer	On
Inline movie	Off
Auto store images	On
Load images to stamp segments	Off
Load images to graphic segments	Off
Auto open inline display	Off
Auto close inline display	Off
Start measurement without further preparation	Off
Wait for user to start	Off
Start measurements	Single measurement

Routine

Slice group	1
Slices	12
Dist. factor	0 %
Position	L2.2 P5.2 H52.0 mm
Orientation	T > C5.2
Phase enc. dir.	A >> P
AutoAlign	---
Phase oversampling	0 %
FoV read	64 mm
FoV phase	78.1 %
Slice thickness	5.0 mm
TR	150 ms
TE	85.0 ms
Averages	1
Concatenations	12
Filter	Raw filter, Prescan Normalize
Coil elements	HC7;NC1,2

Contrast - Common

TR	150 ms
TE	85.0 ms
MTC	Off
Magn. preparation	None
Fat suppr.	Fat sat.
Fat sat. mode	Strong

Contrast - Dynamic

Averages	1
Averaging mode	Long term
Reconstruction	Magnitude
Measurements	1
Delay in TR	0 ms
Multiple series	Off

Resolution - Common

FoV read	64 mm
FoV phase	78.1 %
Slice thickness	5.0 mm
Base resolution	64
Phase resolution	100 %
Phase partial Fourier	6/8
Interpolation	Off

Resolution - iPAT

Accel. mode	None

Resolution - Filter Image

Distortion Corr.	Off
Prescan Normalize	On
Dynamic Field Corr.	Off

Resolution - Filter Rawdata

Raw filter	On
Elliptical filter	Off

Geometry - Common

Slice group	1
Slices	12
Dist. factor	0 %
Position	L2.2 P5.2 H52.0 mm
Orientation	T > C5.2
Phase enc. dir.	A >> P
FoV read	64 mm
FoV phase	78.1 %
Slice thickness	5.0 mm
TR	150 ms
Multi-slice mode	Interleaved
Series	Interleaved
Concatenations	12

Geometry - AutoAlign

Slice group	1
Position	L2.2 P5.2 H52.0 mm
Orientation	T > C5.2
Phase enc. dir.	A >> P
AutoAlign	---
Initial Position	L2.2 P5.2 H38.0
Phase	8.6 mm
Read	-2.2 mm
Shift	37.4 mm
Initial Rotation	0.00 deg
Initial Orientation	T > C
T > C	5.2
> S	0.0

Geometry - Saturation

Fat suppr.	Fat sat.
Fat sat. mode	Strong
Special sat.	None

Geometry - Navigator

Geometry - Tim Planning Suite

Set-n-Go Protocol	Off
Table position	H
Table position	14 mm
Inline Composing	Off

System - Miscellaneous

Positioning mode	REF
Table position	H
Table position	14 mm
MSMA	S - C - T
Sagittal	L >> R
Coronal	A >> P
Transversal	H >> F
Coil Combine Mode	Adaptive Combine

System - Miscellaneous

Matrix Optimization	Off
AutoAlign	---
Coil Select Mode	Off - AutoCoilSelect

System - Adjustments

B0 Shim mode	Standard
B1 Shim mode	TrueForm
Adjust with body coil	Off
Confirm freq. adjustment	Off
Assume Dominant Fat	Off
Assume Silicone	Off
Adjustment Tolerance	Auto

System - Adjust Volume

Position	L2.2 P5.2 H52.0 mm
Orientation	T > C5.2
Rotation	0.00 deg
A >> P	50 mm
R >> L	64 mm
F >> H	60 mm
Reset	Off
Couple to	Acquisition volume

System - pTx Volumes

B1 Shim mode	TrueForm
Excitation	ZOOMit
pTx Volume	1
Vol. Property	Optimization Vol.
Position	L2.2 P5.2 H52.0 mm
Orientation	T > C5.2
Rotation	0 deg
A >> P	250 mm
R >> L	64 mm
F >> H	60 mm
Vol. Visibility	On

System - Tx/Rx

Frequency 1H	123.256882 MHz
Correction factor	1
Gain	High
Img. Scale Cor.	1.000
Reset	Off
? Ref. amplitude 1H	0.000 V

Physio - Signal1

1st Signal/Mode	Pulse/Trigger
Average cycle	973 ± 40 ms
Average cycle	971 ± 40 ms
Acquisition window	650 ms
Trigger pulse	1
Trigger delay	200 ms
TR	150 ms
Concatenations	12
Phases	1

Physio - PACE

Resp. control	Off
Concatenations	12

Diff - Neuro

Diffusion mode	MDDW
Diff. directions	6
Diffusion Scheme	Monopolar

Diff - Neuro

Diff. weightings	2
b-value 1	0 s/mm²
b-value 2	300 s/mm²
b-value 1	1
b-value 2	1
Diff. weighted images	On
Trace weighted images	On
ADC maps	Off
FA maps	Off
Mosaic	On
Tensor	Off
Noise level	0

Diff - Body

Diffusion mode	MDDW
Diff. directions	6
Diffusion Scheme	Monopolar
Diff. weightings	2
b-value 1	0 s/mm²
b-value 2	300 s/mm²
b-value 1	1
b-value 2	1
Diff. weighted images	On
Trace weighted images	On
ADC maps	Off
Exponential ADC Maps	Off
FA maps	Off
Invert Gray Scale	Off
Calculated Image	Off
b-Value >=	0 s/mm²
Noise level	0

Diff - Composing

Inline Composing	Off
Distortion Corr.	Off

Sequence - Part 1

Introduction	On
Optimization	None
Multi-slice mode	Interleaved
Free echo spacing	Off
Echo spacing	0.93 ms
Bandwidth	1240 Hz/Px

Sequence - Part 2

EPI factor	50
RF pulse type	Normal
Gradient mode	Performance
Excitation	ZOOMit

Sequence - pTX Pulses

pTX Pulse	1
TX acceleration	1.0

***** Anatomical T2*-weighted (MEDIC) *****

Properties

Prio recon	Off
Load images to viewer	On
Inline movie	Off
Auto store images	On
Load images to stamp segments	Off
Load images to graphic segments	Off
Auto open inline display	Off
Auto close inline display	Off
Start measurement without further preparation	Off
Wait for user to start	Off
Start measurements	Single measurement

Routine

Slice group	1
Slices	12
Dist. factor	0 %
Position	L2.2 P5.2 H52.0 mm
Orientation	T > C5.2
Phase enc. dir.	A >> P
AutoAlign	---
Phase oversampling	50 %
FoV read	128 mm
FoV phase	100.0 %
Slice thickness	5.0 mm
TR	625.0 ms
TE	15.0 ms
Averages	3
Concatenations	1
Filter	Distortion Corr.(2D), Normalize, Elliptical filter
Coil elements	HC7;NC1,2

Contrast - Common

TR	625.0 ms
TE	15.0 ms
MTC	Off
Flip angle	30 deg
Fat suppr.	None
Water suppr.	None

Contrast - Dynamic

Averages	3
Averaging mode	Long term
Reconstruction	Magnitude
Measurements	1
Multiple series	Off

Resolution - Common

FoV read	128 mm
FoV phase	100.0 %
Slice thickness	5.0 mm
Base resolution	256
Phase resolution	100 %
Phase partial Fourier	Off
Interpolation	Off

Resolution - iPAT

PAT mode	GRAPPA
Accel. factor PE	2

Resolution - iPAT

Ref. lines PE	24
Reference scan mode	Integrated

Resolution - Filter Image

Image Filter	Off
Distortion Corr.	On
Mode	2D
Unfiltered images	Off
Prescan Normalize	Off
Normalize	On
B1 filter	Off

Resolution - Filter Rawdata

Raw filter	Off
Elliptical filter	On

Geometry - Common

Slice group	1
Slices	12
Dist. factor	0 %
Position	L2.2 P5.2 H52.0 mm
Orientation	T > C5.2
Phase enc. dir.	A >> P
FoV read	128 mm
FoV phase	100.0 %
Slice thickness	5.0 mm
TR	625.0 ms
Multi-slice mode	Interleaved
Series	Interleaved
Concatenations	1

Geometry - AutoAlign

Slice group	1
Position	L2.2 P5.2 H52.0 mm
Orientation	T > C5.2
Phase enc. dir.	A >> P
AutoAlign	---
Initial Position	L2.2 P5.2 H38.0
Phase	8.6 mm
Read	-2.2 mm
Shift	37.4 mm
Initial Rotation	0.00 deg
Initial Orientation	T > C
T > C	5.2
> S	0.0

Geometry - Saturation

Sat. region	1
Thickness	59 mm
Position	R0.5 A46.1 F6.6 mm
Orientation	C > T-8.2 > S-0.6
Water suppr.	None
Special sat.	None

Geometry - Tim Planning Suite

Set-n-Go Protocol	Off
Table position	H
Table position	14 mm
Inline Composing	Off

System - Miscellaneous

Positioning mode	REF
Table position	H
Table position	14 mm
MSMA	S - C - T
Sagittal	L >> R
Coronal	A >> P
Transversal	H >> F
Coil Combine Mode	Adaptive Combine
Save uncombined	Off
Matrix Optimization	Off
AutoAlign	---
Coil Select Mode	Default

System - Adjustments

B0 Shim mode	Standard
B1 Shim mode	TrueForm
Adjust with body coil	Off
Confirm freq. adjustment	Off
Assume Dominant Fat	Off
Assume Silicone	Off
Adjustment Tolerance	Auto

System - Adjust Volume

Position	L2.2 P5.2 H52.0 mm
Orientation	T > C5.2
Rotation	0.00 deg
A >> P	128 mm
R >> L	128 mm
F >> H	60 mm
Reset	Off

System - pTx Volumes

B1 Shim mode	TrueForm

System - Tx/Rx

Frequency 1H	123.256882 MHz
Correction factor	1
Gain	High
Img. Scale Cor.	1.000
Reset	Off
? Ref. amplitude 1H	0.000 V

Physio - Signal1

1st Signal/Mode	None
TR	625.0 ms
Concatenations	1

Inline - Common

Subtract	Off
Measurements	1
StdDev	Off
Save original images	On

Inline - MIP

MIP-Sag	Off
MIP-Cor	Off
MIP-Tra	Off
MIP-Time	Off
Save original images	On

Inline - Composing

Inline Composing	Off
Distortion Corr.	On

Inline - Composing

Mode	2D
Unfiltered images	Off

Sequence - Part 1

Introduction	On
Dimension	2D
Combined echoes	3
Flow comp.	Yes
Multi-slice mode	Interleaved
Bandwidth	260 Hz/Px

Sequence - Part 2

RF pulse type	Normal
Gradient mode	Fast
RF spoiling	On

Sequence - Assistant

Mode	Off

***** Multi-TE, TE = 200 ms *****

Properties

Prio recon	Off
Load images to viewer	On
Inline movie	Off
Auto store images	On
Load images to stamp segments	Off
Load images to graphic segments	Off
Auto open inline display	Off
Auto close inline display	Off
Start measurement without further preparation	Off
Wait for user to start	Off
Start measurements	Single measurement

Routine

Slice group	1
Slices	12
Dist. factor	0 %
Position	L2.2 P5.2 H52.0 mm
Orientation	T > C5.2
Phase enc. dir.	A >> P
AutoAlign	---
Phase oversampling	0 %
FoV read	64 mm
FoV phase	78.1 %
Slice thickness	5.0 mm
TR	270 ms
TE	200.0 ms
Averages	1
Concatenations	12
Filter	Raw filter, Prescan Normalize
Coil elements	HC7;NC1,2

Contrast - Common

TR	270 ms
TE	200.0 ms
MTC	Off
Magn. preparation	None
Fat suppr.	Fat sat.
Fat sat. mode	Strong

Contrast - Dynamic

Averages	1
Averaging mode	Long term
Reconstruction	Magnitude
Measurements	1
Delay in TR	0 ms
Multiple series	Off

Resolution - Common

FoV read	64 mm
FoV phase	78.1 %
Slice thickness	5.0 mm
Base resolution	64
Phase resolution	100 %
Phase partial Fourier	6/8
Interpolation	Off

Resolution - iPAT

Accel. mode	None

Resolution - Filter Image

Distortion Corr.	Off
Prescan Normalize	On
Dynamic Field Corr.	Off

Resolution - Filter Rawdata

Raw filter	On
Elliptical filter	Off

Geometry - Common

Slice group	1
Slices	12
Dist. factor	0 %
Position	L2.2 P5.2 H52.0 mm
Orientation	T > C5.2
Phase enc. dir.	A >> P
FoV read	64 mm
FoV phase	78.1 %
Slice thickness	5.0 mm
TR	270 ms
Multi-slice mode	Interleaved
Series	Interleaved
Concatenations	12

Geometry - AutoAlign

Slice group	1
Position	L2.2 P5.2 H52.0 mm
Orientation	T > C5.2
Phase enc. dir.	A >> P
AutoAlign	---
Initial Position	L2.2 P5.2 H38.0
Phase	8.6 mm
Read	-2.2 mm
Shift	37.4 mm
Initial Rotation	0.00 deg
Initial Orientation	T > C
T > C	5.2
> S	0.0

Geometry - Saturation

Fat suppr.	Fat sat.
Fat sat. mode	Strong
Special sat.	None

Geometry - Navigator

Geometry - Tim Planning Suite

Set-n-Go Protocol	Off
Table position	H
Table position	14 mm
Inline Composing	Off

System - Miscellaneous

Positioning mode	REF
Table position	H
Table position	14 mm
MSMA	S - C - T
Sagittal	L >> R
Coronal	A >> P
Transversal	H >> F
Coil Combine Mode	Adaptive Combine

System - Miscellaneous

Matrix Optimization	Off
AutoAlign	---
Coil Select Mode	Off - AutoCoilSelect

System - Adjustments

B0 Shim mode	Standard
B1 Shim mode	TrueForm
Adjust with body coil	Off
Confirm freq. adjustment	Off
Assume Dominant Fat	Off
Assume Silicone	Off
Adjustment Tolerance	Auto

System - Adjust Volume

Position	L2.2 P5.2 H52.0 mm
Orientation	T > C5.2
Rotation	0.00 deg
A >> P	50 mm
R >> L	64 mm
F >> H	60 mm
Reset	Off
Couple to	Acquisition volume

System - pTx Volumes

B1 Shim mode	TrueForm
Excitation	ZOOMit
pTx Volume	1
Vol. Property	Optimization Vol.
Position	L2.2 P5.2 H52.0 mm
Orientation	T > C5.2
Rotation	0 deg
A >> P	250 mm
R >> L	64 mm
F >> H	60 mm
Vol. Visibility	On

System - Tx/Rx

Frequency 1H	123.256882 MHz
Correction factor	1
Gain	High
Img. Scale Cor.	1.000
Reset	Off
? Ref. amplitude 1H	0.000 V

Physio - Signal1

1st Signal/Mode	Pulse/Trigger
Average cycle	970 ± 37 ms
Average cycle	960 ± 37 ms
Acquisition window	650 ms
Trigger pulse	1
Trigger delay	200 ms
TR	270 ms
Concatenations	12
Phases	1

Physio - PACE

Resp. control	Off
Concatenations	12

Diff - Neuro

Diffusion mode	MDDW
Diff. directions	30
Diffusion Scheme	Monopolar

Diff - Neuro

Diff. weightings	1
b-value	0 s/mm²
b-value	1
Diff. weighted images	On
Trace weighted images	Off
ADC maps	Off
FA maps	Off
Mosaic	Off
Tensor	Off
Noise level	0

Diff - Body

Diffusion mode	MDDW
Diff. directions	30
Diffusion Scheme	Monopolar
Diff. weightings	1
b-value	0 s/mm²
b-value	1
Diff. weighted images	On
Trace weighted images	Off
ADC maps	Off
Exponential ADC Maps	Off
FA maps	Off
Invert Gray Scale	Off
Calculated Image	Off
b-Value >=	0 s/mm²
Noise level	0

Diff - Composing

Inline Composing	Off
Distortion Corr.	Off

Sequence - Part 1

Introduction	On
Optimization	None
Multi-slice mode	Interleaved
Free echo spacing	Off
Echo spacing	0.93 ms
Bandwidth	1240 Hz/Px

Sequence - Part 2

EPI factor	50
RF pulse type	Normal
Gradient mode	Performance
Excitation	ZOOMit

Sequence - pTX Pulses

pTX Pulse	1
TX acceleration	1.0

***** Multi-TE, TE = 120 ms *****

Properties

Prio recon	Off
Load images to viewer	On
Inline movie	Off
Auto store images	On
Load images to stamp segments	Off
Load images to graphic segments	Off
Auto open inline display	Off
Auto close inline display	Off
Start measurement without further preparation	Off
Wait for user to start	Off
Start measurements	Single measurement

Routine

Slice group	1
Slices	12
Dist. factor	0 %
Position	L2.2 P5.2 H52.0 mm
Orientation	T > C5.2
Phase enc. dir.	A >> P
AutoAlign	---
Phase oversampling	0 %
FoV read	64 mm
FoV phase	78.1 %
Slice thickness	5.0 mm
TR	270 ms
TE	120.0 ms
Averages	1
Concatenations	12
Filter	Raw filter, Prescan Normalize
Coil elements	HC7;NC1,2

Contrast - Common

TR	270 ms
TE	120.0 ms
MTC	Off
Magn. preparation	None
Fat suppr.	Fat sat.
Fat sat. mode	Strong

Contrast - Dynamic

Averages	1
Averaging mode	Long term
Reconstruction	Magnitude
Measurements	1
Delay in TR	0 ms
Multiple series	Off

Resolution - Common

FoV read	64 mm
FoV phase	78.1 %
Slice thickness	5.0 mm
Base resolution	64
Phase resolution	100 %
Phase partial Fourier	6/8
Interpolation	Off

Resolution - iPAT

Accel. mode	None

Resolution - Filter Image

Distortion Corr.	Off
Prescan Normalize	On
Dynamic Field Corr.	Off

Resolution - Filter Rawdata

Raw filter	On
Elliptical filter	Off

Geometry - Common

Slice group	1
Slices	12
Dist. factor	0 %
Position	L2.2 P5.2 H52.0 mm
Orientation	T > C5.2
Phase enc. dir.	A >> P
FoV read	64 mm
FoV phase	78.1 %
Slice thickness	5.0 mm
TR	270 ms
Multi-slice mode	Interleaved
Series	Interleaved
Concatenations	12

Geometry - AutoAlign

Slice group	1
Position	L2.2 P5.2 H52.0 mm
Orientation	T > C5.2
Phase enc. dir.	A >> P
AutoAlign	---
Initial Position	L2.2 P5.2 H38.0
Phase	8.6 mm
Read	-2.2 mm
Shift	37.4 mm
Initial Rotation	0.00 deg
Initial Orientation	T > C
T > C	5.2
> S	0.0

Geometry - Saturation

Fat suppr.	Fat sat.
Fat sat. mode	Strong
Special sat.	None

Geometry - Navigator

Geometry - Tim Planning Suite

Set-n-Go Protocol	Off
Table position	H
Table position	14 mm
Inline Composing	Off

System - Miscellaneous

Positioning mode	REF
Table position	H
Table position	14 mm
MSMA	S - C - T
Sagittal	L >> R
Coronal	A >> P
Transversal	H >> F
Coil Combine Mode	Adaptive Combine

System - Miscellaneous

Matrix Optimization	Off
AutoAlign	---
Coil Select Mode	Off - AutoCoilSelect

System - Adjustments

B0 Shim mode	Standard
B1 Shim mode	TrueForm
Adjust with body coil	Off
Confirm freq. adjustment	Off
Assume Dominant Fat	Off
Assume Silicone	Off
Adjustment Tolerance	Auto

System - Adjust Volume

Position	L2.2 P5.2 H52.0 mm
Orientation	T > C5.2
Rotation	0.00 deg
A >> P	50 mm
R >> L	64 mm
F >> H	60 mm
Reset	Off
Couple to	Acquisition volume

System - pTx Volumes

B1 Shim mode	TrueForm
Excitation	ZOOMit
pTx Volume	1
Vol. Property	Optimization Vol.
Position	L2.2 P5.2 H52.0 mm
Orientation	T > C5.2
Rotation	0 deg
A >> P	250 mm
R >> L	64 mm
F >> H	60 mm
Vol. Visibility	On

System - Tx/Rx

Frequency 1H	123.256882 MHz
Correction factor	1
Gain	High
Img. Scale Cor.	1.000
Reset	Off
? Ref. amplitude 1H	0.000 V

Physio - Signal1

1st Signal/Mode	Pulse/Trigger
Average cycle	969 ± 29 ms
Average cycle	985 ± 29 ms
Acquisition window	650 ms
Trigger pulse	1
Trigger delay	200 ms
TR	270 ms
Concatenations	12
Phases	1

Physio - PACE

Resp. control	Off
Concatenations	12

Diff - Neuro

Diffusion mode	MDDW
Diff. directions	30
Diffusion Scheme	Monopolar

Diff - Neuro

Diff. weightings	1
b-value	0 s/mm²
b-value	1
Diff. weighted images	On
Trace weighted images	Off
ADC maps	Off
FA maps	Off
Mosaic	Off
Tensor	Off
Noise level	0

Diff - Body

Diffusion mode	MDDW
Diff. directions	30
Diffusion Scheme	Monopolar
Diff. weightings	1
b-value	0 s/mm²
b-value	1
Diff. weighted images	On
Trace weighted images	Off
ADC maps	Off
Exponential ADC Maps	Off
FA maps	Off
Invert Gray Scale	Off
Calculated Image	Off
b-Value >=	0 s/mm²
Noise level	0

Diff - Composing

Inline Composing	Off
Distortion Corr.	Off

Sequence - Part 1

Introduction	On
Optimization	None
Multi-slice mode	Interleaved
Free echo spacing	Off
Echo spacing	0.93 ms
Bandwidth	1240 Hz/Px

Sequence - Part 2

EPI factor	50
RF pulse type	Normal
Gradient mode	Performance
Excitation	ZOOMit

Sequence - pTX Pulses

pTX Pulse	1
TX acceleration	1.0

***** Multi-TE, TE = 105 ms *****

Properties

Prio recon	Off
Load images to viewer	On
Inline movie	Off
Auto store images	On
Load images to stamp segments	Off
Load images to graphic segments	Off
Auto open inline display	Off
Auto close inline display	Off
Start measurement without further preparation	Off
Wait for user to start	Off
Start measurements	Single measurement

Routine

Slice group	1
Slices	12
Dist. factor	0 %
Position	L2.2 P5.2 H52.0 mm
Orientation	T > C5.2
Phase enc. dir.	A >> P
AutoAlign	---
Phase oversampling	0 %
FoV read	64 mm
FoV phase	78.1 %
Slice thickness	5.0 mm
TR	270 ms
TE	105.0 ms
Averages	1
Concatenations	12
Filter	Raw filter, Prescan Normalize
Coil elements	HC7;NC1,2

Contrast - Common

TR	270 ms
TE	105.0 ms
MTC	Off
Magn. preparation	None
Fat suppr.	Fat sat.
Fat sat. mode	Strong

Contrast - Dynamic

Averages	1
Averaging mode	Long term
Reconstruction	Magnitude
Measurements	1
Delay in TR	0 ms
Multiple series	Off

Resolution - Common

FoV read	64 mm
FoV phase	78.1 %
Slice thickness	5.0 mm
Base resolution	64
Phase resolution	100 %
Phase partial Fourier	6/8
Interpolation	Off

Resolution - iPAT

Accel. mode	None

Resolution - Filter Image

Distortion Corr.	Off
Prescan Normalize	On
Dynamic Field Corr.	Off

Resolution - Filter Rawdata

Raw filter	On
Elliptical filter	Off

Geometry - Common

Slice group	1
Slices	12
Dist. factor	0 %
Position	L2.2 P5.2 H52.0 mm
Orientation	T > C5.2
Phase enc. dir.	A >> P
FoV read	64 mm
FoV phase	78.1 %
Slice thickness	5.0 mm
TR	270 ms
Multi-slice mode	Interleaved
Series	Interleaved
Concatenations	12

Geometry - AutoAlign

Slice group	1
Position	L2.2 P5.2 H52.0 mm
Orientation	T > C5.2
Phase enc. dir.	A >> P
AutoAlign	---
Initial Position	L2.2 P5.2 H38.0
Phase	8.6 mm
Read	-2.2 mm
Shift	37.4 mm
Initial Rotation	0.00 deg
Initial Orientation	T > C
T > C	5.2
> S	0.0

Geometry - Saturation

Fat suppr.	Fat sat.
Fat sat. mode	Strong
Special sat.	None

Geometry - Navigator

Geometry - Tim Planning Suite

Set-n-Go Protocol	Off
Table position	H
Table position	14 mm
Inline Composing	Off

System - Miscellaneous

Positioning mode	REF
Table position	H
Table position	14 mm
MSMA	S - C - T
Sagittal	L >> R
Coronal	A >> P
Transversal	H >> F
Coil Combine Mode	Adaptive Combine

System - Miscellaneous

Matrix Optimization	Off
AutoAlign	---
Coil Select Mode	Off - AutoCoilSelect

System - Adjustments

B0 Shim mode	Standard
B1 Shim mode	TrueForm
Adjust with body coil	Off
Confirm freq. adjustment	Off
Assume Dominant Fat	Off
Assume Silicone	Off
Adjustment Tolerance	Auto

System - Adjust Volume

Position	L2.2 P5.2 H52.0 mm
Orientation	T > C5.2
Rotation	0.00 deg
A >> P	50 mm
R >> L	64 mm
F >> H	60 mm
Reset	Off
Couple to	Acquisition volume

System - pTx Volumes

B1 Shim mode	TrueForm
Excitation	ZOOMit
pTx Volume	1
Vol. Property	Optimization Vol.
Position	L2.2 P5.2 H52.0 mm
Orientation	T > C5.2
Rotation	0 deg
A >> P	250 mm
R >> L	64 mm
F >> H	60 mm
Vol. Visibility	On

System - Tx/Rx

Frequency 1H	123.256882 MHz
Correction factor	1
Gain	High
Img. Scale Cor.	1.000
Reset	Off
? Ref. amplitude 1H	0.000 V

Physio - Signal1

1st Signal/Mode	Pulse/Trigger
Average cycle	969 ± 36 ms
Average cycle	968 ± 36 ms
Acquisition window	650 ms
Trigger pulse	1
Trigger delay	200 ms
TR	270 ms
Concatenations	12
Phases	1

Physio - PACE

Resp. control	Off
Concatenations	12

Diff - Neuro

Diffusion mode	MDDW
Diff. directions	30
Diffusion Scheme	Monopolar

Diff - Neuro

Diff. weightings	1
b-value	0 s/mm²
b-value	1
Diff. weighted images	On
Trace weighted images	Off
ADC maps	Off
FA maps	Off
Mosaic	Off
Tensor	Off
Noise level	0

Diff - Body

Diffusion mode	MDDW
Diff. directions	30
Diffusion Scheme	Monopolar
Diff. weightings	1
b-value	0 s/mm²
b-value	1
Diff. weighted images	On
Trace weighted images	Off
ADC maps	Off
Exponential ADC Maps	Off
FA maps	Off
Invert Gray Scale	Off
Calculated Image	Off
b-Value >=	0 s/mm²
Noise level	0

Diff - Composing

Inline Composing	Off
Distortion Corr.	Off

Sequence - Part 1

Introduction	On
Optimization	None
Multi-slice mode	Interleaved
Free echo spacing	Off
Echo spacing	0.93 ms
Bandwidth	1240 Hz/Px

Sequence - Part 2

EPI factor	50
RF pulse type	Normal
Gradient mode	Performance
Excitation	ZOOMit

Sequence - pTX Pulses

pTX Pulse	1
TX acceleration	1.0

***** Multi-TE, TE = 90 ms *****

Properties

Prio recon	Off
Load images to viewer	On
Inline movie	Off
Auto store images	On
Load images to stamp segments	Off
Load images to graphic segments	Off
Auto open inline display	Off
Auto close inline display	Off
Start measurement without further preparation	Off
Wait for user to start	Off
Start measurements	Single measurement

Routine

Slice group	1
Slices	12
Dist. factor	0 %
Position	L2.2 P5.2 H52.0 mm
Orientation	T > C5.2
Phase enc. dir.	A >> P
AutoAlign	---
Phase oversampling	0 %
FoV read	64 mm
FoV phase	78.1 %
Slice thickness	5.0 mm
TR	270 ms
TE	90.0 ms
Averages	1
Concatenations	12
Filter	Raw filter, Prescan Normalize
Coil elements	HC7;NC1,2

Contrast - Common

TR	270 ms
TE	90.0 ms
MTC	Off
Magn. preparation	None
Fat suppr.	Fat sat.
Fat sat. mode	Strong

Contrast - Dynamic

Averages	1
Averaging mode	Long term
Reconstruction	Magnitude
Measurements	1
Delay in TR	0 ms
Multiple series	Off

Resolution - Common

FoV read	64 mm
FoV phase	78.1 %
Slice thickness	5.0 mm
Base resolution	64
Phase resolution	100 %
Phase partial Fourier	6/8
Interpolation	Off

Resolution - iPAT

Accel. mode	None

Resolution - Filter Image

Distortion Corr.	Off
Prescan Normalize	On
Dynamic Field Corr.	Off

Resolution - Filter Rawdata

Raw filter	On
Elliptical filter	Off

Geometry - Common

Slice group	1
Slices	12
Dist. factor	0 %
Position	L2.2 P5.2 H52.0 mm
Orientation	T > C5.2
Phase enc. dir.	A >> P
FoV read	64 mm
FoV phase	78.1 %
Slice thickness	5.0 mm
TR	270 ms
Multi-slice mode	Interleaved
Series	Interleaved
Concatenations	12

Geometry - AutoAlign

Slice group	1
Position	L2.2 P5.2 H52.0 mm
Orientation	T > C5.2
Phase enc. dir.	A >> P
AutoAlign	---
Initial Position	L2.2 P5.2 H38.0
Phase	8.6 mm
Read	-2.2 mm
Shift	37.4 mm
Initial Rotation	0.00 deg
Initial Orientation	T > C
T > C	5.2
> S	0.0

Geometry - Saturation

Fat suppr.	Fat sat.
Fat sat. mode	Strong
Special sat.	None

Geometry - Navigator

Geometry - Tim Planning Suite

Set-n-Go Protocol	Off
Table position	H
Table position	14 mm
Inline Composing	Off

System - Miscellaneous

Positioning mode	REF
Table position	H
Table position	14 mm
MSMA	S - C - T
Sagittal	L >> R
Coronal	A >> P
Transversal	H >> F
Coil Combine Mode	Adaptive Combine

System - Miscellaneous

Matrix Optimization	Off
AutoAlign	---
Coil Select Mode	Off - AutoCoilSelect

System - Adjustments

B0 Shim mode	Standard
B1 Shim mode	TrueForm
Adjust with body coil	Off
Confirm freq. adjustment	Off
Assume Dominant Fat	Off
Assume Silicone	Off
Adjustment Tolerance	Auto

System - Adjust Volume

Position	L2.2 P5.2 H52.0 mm
Orientation	T > C5.2
Rotation	0.00 deg
A >> P	50 mm
R >> L	64 mm
F >> H	60 mm
Reset	Off
Couple to	Acquisition volume

System - pTx Volumes

B1 Shim mode	TrueForm
Excitation	ZOOMit
pTx Volume	1
Vol. Property	Optimization Vol.
Position	L2.2 P5.2 H52.0 mm
Orientation	T > C5.2
Rotation	0 deg
A >> P	250 mm
R >> L	64 mm
F >> H	60 mm
Vol. Visibility	On

System - Tx/Rx

Frequency 1H	123.256882 MHz
Correction factor	1
Gain	High
Img. Scale Cor.	1.000
Reset	Off
? Ref. amplitude 1H	0.000 V

Physio - Signal1

1st Signal/Mode	Pulse/Trigger
Average cycle	968 ± 34 ms
Average cycle	958 ± 34 ms
Acquisition window	650 ms
Trigger pulse	1
Trigger delay	200 ms
TR	270 ms
Concatenations	12
Phases	1

Physio - PACE

Resp. control	Off
Concatenations	12

Diff - Neuro

Diffusion mode	MDDW
Diff. directions	30
Diffusion Scheme	Monopolar
Diff. weightings	1
b-value	0 s/mm²
b-value	1
Diff. weighted images	On
Trace weighted images	Off
ADC maps	Off
FA maps	Off
Mosaic	Off
Tensor	Off
Noise level	0

Diff - Body

Diffusion mode	MDDW
Diff. directions	30
Diffusion Scheme	Monopolar
Diff. weightings	1
b-value	0 s/mm²
b-value	1
Diff. weighted images	On
Trace weighted images	Off
ADC maps	Off
Exponential ADC Maps	Off
FA maps	Off
Invert Gray Scale	Off
Calculated Image	Off
b-Value >=	0 s/mm²
Noise level	0

Diff - Composing

Inline Composing	Off
Distortion Corr.	Off

Sequence - Part 1

Introduction	On
Optimization	None
Multi-slice mode	Interleaved
Free echo spacing	Off
Echo spacing	0.93 ms
Bandwidth	1240 Hz/Px

Sequence - Part 2

EPI factor	50
RF pulse type	Normal
Gradient mode	Performance
Excitation	ZOOMit

Sequence - pTX Pulses

pTX Pulse	1
TX acceleration	1.0

***** Multi-TE, TE = 75 ms *****

Properties

Prio recon	Off
Load images to viewer	On
Inline movie	Off
Auto store images	On
Load images to stamp segments	Off
Load images to graphic segments	Off
Auto open inline display	Off
Auto close inline display	Off
Start measurement without further preparation	Off
Wait for user to start	Off
Start measurements	Single measurement

Routine

Slice group	1
Slices	12
Dist. factor	0 %
Position	L2.2 P5.2 H52.0 mm
Orientation	T > C5.2
Phase enc. dir.	A >> P
AutoAlign	---
Phase oversampling	0 %
FoV read	64 mm
FoV phase	78.1 %
Slice thickness	5.0 mm
TR	270 ms
TE	75.0 ms
Averages	1
Concatenations	12
Filter	Raw filter, Prescan Normalize
Coil elements	HC7;NC1,2

Contrast - Common

TR	270 ms
TE	75.0 ms
MTC	Off
Magn. preparation	None
Fat suppr.	Fat sat.
Fat sat. mode	Strong

Contrast - Dynamic

Averages	1
Averaging mode	Long term
Reconstruction	Magnitude
Measurements	1
Delay in TR	0 ms
Multiple series	Off

Resolution - Common

FoV read	64 mm
FoV phase	78.1 %
Slice thickness	5.0 mm
Base resolution	64
Phase resolution	100 %
Phase partial Fourier	6/8
Interpolation	Off

Resolution - iPAT

Accel. mode	None

Resolution - Filter Image

Distortion Corr.	Off
Prescan Normalize	On
Dynamic Field Corr.	Off

Resolution - Filter Rawdata

Raw filter	On
Elliptical filter	Off

Geometry - Common

Slice group	1
Slices	12
Dist. factor	0 %
Position	L2.2 P5.2 H52.0 mm
Orientation	T > C5.2
Phase enc. dir.	A >> P
FoV read	64 mm
FoV phase	78.1 %
Slice thickness	5.0 mm
TR	270 ms
Multi-slice mode	Interleaved
Series	Interleaved
Concatenations	12

Geometry - AutoAlign

Slice group	1
Position	L2.2 P5.2 H52.0 mm
Orientation	T > C5.2
Phase enc. dir.	A >> P
AutoAlign	---
Initial Position	L2.2 P5.2 H38.0
Phase	8.6 mm
Read	-2.2 mm
Shift	37.4 mm
Initial Rotation	0.00 deg
Initial Orientation	T > C
T > C	5.2
> S	0.0

Geometry - Saturation

Fat suppr.	Fat sat.
Fat sat. mode	Strong
Special sat.	None

Geometry - Navigator

Geometry - Tim Planning Suite

Set-n-Go Protocol	Off
Table position	H
Table position	14 mm
Inline Composing	Off

System - Miscellaneous

Positioning mode	REF
Table position	H
Table position	14 mm
MSMA	S - C - T
Sagittal	L >> R
Coronal	A >> P
Transversal	H >> F
Coil Combine Mode	Adaptive Combine

System - Miscellaneous

Matrix Optimization	Off
AutoAlign	---
Coil Select Mode	Off - AutoCoilSelect

System - Adjustments

B0 Shim mode	Standard
B1 Shim mode	TrueForm
Adjust with body coil	Off
Confirm freq. adjustment	Off
Assume Dominant Fat	Off
Assume Silicone	Off
Adjustment Tolerance	Auto

System - Adjust Volume

Position	L2.2 P5.2 H52.0 mm
Orientation	T > C5.2
Rotation	0.00 deg
A >> P	50 mm
R >> L	64 mm
F >> H	60 mm
Reset	Off
Couple to	Acquisition volume

System - pTx Volumes

B1 Shim mode	TrueForm
Excitation	ZOOMit
pTx Volume	1
Vol. Property	Optimization Vol.
Position	L2.2 P5.2 H52.0 mm
Orientation	T > C5.2
Rotation	0 deg
A >> P	250 mm
R >> L	64 mm
F >> H	60 mm
Vol. Visibility	On

System - Tx/Rx

Frequency 1H	123.256882 MHz
Correction factor	1
Gain	High
Img. Scale Cor.	1.000
Reset	Off
? Ref. amplitude 1H	0.000 V

Physio - Signal1

1st Signal/Mode	Pulse/Trigger
Average cycle	967 ± 33 ms
Average cycle	967 ± 33 ms
Acquisition window	650 ms
Trigger pulse	1
Trigger delay	200 ms
TR	270 ms
Concatenations	12
Phases	1

Physio - PACE

Resp. control	Off
Concatenations	12

Diff - Neuro

Diffusion mode	MDDW
Diff. directions	30
Diffusion Scheme	Monopolar

Diff - Neuro

Diff. weightings	1
b-value	0 s/mm²
b-value	1
Diff. weighted images	On
Trace weighted images	Off
ADC maps	Off
FA maps	Off
Mosaic	Off
Tensor	Off
Noise level	0

Diff - Body

Diffusion mode	MDDW
Diff. directions	30
Diffusion Scheme	Monopolar
Diff. weightings	1
b-value	0 s/mm²
b-value	1
Diff. weighted images	On
Trace weighted images	Off
ADC maps	Off
Exponential ADC Maps	Off
FA maps	Off
Invert Gray Scale	Off
Calculated Image	Off
b-Value >=	0 s/mm²
Noise level	0

Diff - Composing

Inline Composing	Off
Distortion Corr.	Off

Sequence - Part 1

Introduction	On
Optimization	None
Multi-slice mode	Interleaved
Free echo spacing	Off
Echo spacing	0.93 ms
Bandwidth	1240 Hz/Px

Sequence - Part 2

EPI factor	50
RF pulse type	Normal
Gradient mode	Performance
Excitation	ZOOMit

Sequence - pTX Pulses

pTX Pulse	1
TX acceleration	1.0

***** Multi-TE, TE = 60 ms *****

Properties

Prio recon	Off
Load images to viewer	On
Inline movie	Off
Auto store images	On
Load images to stamp segments	Off
Load images to graphic segments	Off
Auto open inline display	Off
Auto close inline display	Off
Start measurement without further preparation	Off
Wait for user to start	Off
Start measurements	Single measurement

Routine

Slice group	1
Slices	12
Dist. factor	0 %
Position	L2.2 P5.2 H52.0 mm
Orientation	T > C5.2
Phase enc. dir.	A >> P
AutoAlign	---
Phase oversampling	0 %
FoV read	64 mm
FoV phase	78.1 %
Slice thickness	5.0 mm
TR	270 ms
TE	60.0 ms
Averages	1
Concatenations	12
Filter	Raw filter, Prescan Normalize
Coil elements	HC7;NC1,2

Contrast - Common

TR	270 ms
TE	60.0 ms
MTC	Off
Magn. preparation	None
Fat suppr.	Fat sat.
Fat sat. mode	Strong

Contrast - Dynamic

Averages	1
Averaging mode	Long term
Reconstruction	Magnitude
Measurements	1
Delay in TR	0 ms
Multiple series	Off

Resolution - Common

FoV read	64 mm
FoV phase	78.1 %
Slice thickness	5.0 mm
Base resolution	64
Phase resolution	100 %
Phase partial Fourier	6/8
Interpolation	Off

Resolution - iPAT

Accel. mode	None

Resolution - Filter Image

Distortion Corr.	Off
Prescan Normalize	On
Dynamic Field Corr.	Off

Resolution - Filter Rawdata

Raw filter	On
Elliptical filter	Off

Geometry - Common

Slice group	1
Slices	12
Dist. factor	0 %
Position	L2.2 P5.2 H52.0 mm
Orientation	T > C5.2
Phase enc. dir.	A >> P
FoV read	64 mm
FoV phase	78.1 %
Slice thickness	5.0 mm
TR	270 ms
Multi-slice mode	Interleaved
Series	Interleaved
Concatenations	12

Geometry - AutoAlign

Slice group	1
Position	L2.2 P5.2 H52.0 mm
Orientation	T > C5.2
Phase enc. dir.	A >> P
AutoAlign	---
Initial Position	L2.2 P5.2 H38.0
Phase	8.6 mm
Read	-2.2 mm
Shift	37.4 mm
Initial Rotation	0.00 deg
Initial Orientation	T > C
T > C	5.2
> S	0.0

Geometry - Saturation

Fat suppr.	Fat sat.
Fat sat. mode	Strong
Special sat.	None

Geometry - Navigator

Geometry - Tim Planning Suite

Set-n-Go Protocol	Off
Table position	H
Table position	14 mm
Inline Composing	Off

System - Miscellaneous

Positioning mode	REF
Table position	H
Table position	14 mm
MSMA	S - C - T
Sagittal	L >> R
Coronal	A >> P
Transversal	H >> F
Coil Combine Mode	Adaptive Combine

System - Miscellaneous

Matrix Optimization	Off
AutoAlign	---
Coil Select Mode	Off - AutoCoilSelect

System - Adjustments

B0 Shim mode	Standard
B1 Shim mode	TrueForm
Adjust with body coil	Off
Confirm freq. adjustment	Off
Assume Dominant Fat	Off
Assume Silicone	Off
Adjustment Tolerance	Auto

System - Adjust Volume

Position	L2.2 P5.2 H52.0 mm
Orientation	T > C5.2
Rotation	0.00 deg
A >> P	50 mm
R >> L	64 mm
F >> H	60 mm
Reset	Off
Couple to	Acquisition volume

System - pTx Volumes

B1 Shim mode	TrueForm
Excitation	ZOOMit
pTx Volume	1
Vol. Property	Optimization Vol.
Position	L2.2 P5.2 H52.0 mm
Orientation	T > C5.2
Rotation	0 deg
A >> P	250 mm
R >> L	64 mm
F >> H	60 mm
Vol. Visibility	On

System - Tx/Rx

Frequency 1H	123.256882 MHz
Correction factor	1
Gain	High
Img. Scale Cor.	1.000
Reset	Off
? Ref. amplitude 1H	0.000 V

Physio - Signal1

1st Signal/Mode	Pulse/Trigger
Average cycle	967 ± 32 ms
Average cycle	971 ± 32 ms
Acquisition window	650 ms
Trigger pulse	1
Trigger delay	200 ms
TR	270 ms
Concatenations	12
Phases	1

Physio - PACE

Resp. control	Off
Concatenations	12

Diff - Neuro

Diffusion mode	MDDW
Diff. directions	30
Diffusion Scheme	Monopolar

Diff - Neuro

Diff. weightings	1
b-value	0 s/mm²
b-value	1
Diff. weighted images	On
Trace weighted images	Off
ADC maps	Off
FA maps	Off
Mosaic	Off
Tensor	Off
Noise level	0

Diff - Body

Diffusion mode	MDDW
Diff. directions	30
Diffusion Scheme	Monopolar
Diff. weightings	1
b-value	0 s/mm²
b-value	1
Diff. weighted images	On
Trace weighted images	Off
ADC maps	Off
Exponential ADC Maps	Off
FA maps	Off
Invert Gray Scale	Off
Calculated Image	Off
b-Value >=	0 s/mm²
Noise level	0

Diff - Composing

Inline Composing	Off
Distortion Corr.	Off

Sequence - Part 1

Introduction	On
Optimization	None
Multi-slice mode	Interleaved
Free echo spacing	Off
Echo spacing	0.93 ms
Bandwidth	1240 Hz/Px

Sequence - Part 2

EPI factor	50
RF pulse type	Normal
Gradient mode	Performance
Excitation	ZOOMit

Sequence - pTX Pulses

pTX Pulse	1
TX acceleration	1.0

***** Multi-TE, TE = 45 ms *****

Properties

Prio recon	Off
Load images to viewer	On
Inline movie	Off
Auto store images	On
Load images to stamp segments	Off
Load images to graphic segments	Off
Auto open inline display	Off
Auto close inline display	Off
Start measurement without further preparation	Off
Wait for user to start	Off
Start measurements	Single measurement

Routine

Slice group	1
Slices	12
Dist. factor	0 %
Position	L2.2 P5.2 H52.0 mm
Orientation	T > C5.2
Phase enc. dir.	A >> P
AutoAlign	---
Phase oversampling	0 %
FoV read	64 mm
FoV phase	78.1 %
Slice thickness	5.0 mm
TR	270 ms
TE	45.0 ms
Averages	1
Concatenations	12
Filter	Raw filter, Prescan Normalize
Coil elements	HC7;NC1,2

Contrast - Common

TR	270 ms
TE	45.0 ms
MTC	Off
Magn. preparation	None
Fat suppr.	Fat sat.
Fat sat. mode	Strong

Contrast - Dynamic

Averages	1
Averaging mode	Long term
Reconstruction	Magnitude
Measurements	1
Delay in TR	0 ms
Multiple series	Off

Resolution - Common

FoV read	64 mm
FoV phase	78.1 %
Slice thickness	5.0 mm
Base resolution	64
Phase resolution	100 %
Phase partial Fourier	6/8
Interpolation	Off

Resolution - iPAT

Accel. mode	None

Resolution - Filter Image

Distortion Corr.	Off
Prescan Normalize	On
Dynamic Field Corr.	Off

Resolution - Filter Rawdata

Raw filter	On
Elliptical filter	Off

Geometry - Common

Slice group	1
Slices	12
Dist. factor	0 %
Position	L2.2 P5.2 H52.0 mm
Orientation	T > C5.2
Phase enc. dir.	A >> P
FoV read	64 mm
FoV phase	78.1 %
Slice thickness	5.0 mm
TR	270 ms
Multi-slice mode	Interleaved
Series	Interleaved
Concatenations	12

Geometry - AutoAlign

Slice group	1
Position	L2.2 P5.2 H52.0 mm
Orientation	T > C5.2
Phase enc. dir.	A >> P
AutoAlign	---
Initial Position	L2.2 P5.2 H38.0
Phase	8.6 mm
Read	-2.2 mm
Shift	37.4 mm
Initial Rotation	0.00 deg
Initial Orientation	T > C
T > C	5.2
> S	0.0

Geometry - Saturation

Fat suppr.	Fat sat.
Fat sat. mode	Strong
Special sat.	None

Geometry - Navigator

Geometry - Tim Planning Suite

Set-n-Go Protocol	Off
Table position	H
Table position	14 mm
Inline Composing	Off

System - Miscellaneous

Positioning mode	REF
Table position	H
Table position	14 mm
MSMA	S - C - T
Sagittal	L >> R
Coronal	A >> P
Transversal	H >> F
Coil Combine Mode	Adaptive Combine

System - Miscellaneous

Matrix Optimization	Off
AutoAlign	---
Coil Select Mode	Off - AutoCoilSelect

System - Adjustments

B0 Shim mode	Standard
B1 Shim mode	TrueForm
Adjust with body coil	Off
Confirm freq. adjustment	Off
Assume Dominant Fat	Off
Assume Silicone	Off
Adjustment Tolerance	Auto

System - Adjust Volume

Position	L2.2 P5.2 H52.0 mm
Orientation	T > C5.2
Rotation	0.00 deg
A >> P	50 mm
R >> L	64 mm
F >> H	60 mm
Reset	Off
Couple to	Acquisition volume

System - pTx Volumes

B1 Shim mode	TrueForm
Excitation	ZOOMit
pTx Volume	1
Vol. Property	Optimization Vol.
Position	L2.2 P5.2 H52.0 mm
Orientation	T > C5.2
Rotation	0 deg
A >> P	250 mm
R >> L	64 mm
F >> H	60 mm
Vol. Visibility	On

System - Tx/Rx

Frequency 1H	123.256882 MHz
Correction factor	1
Gain	High
Img. Scale Cor.	1.000
Reset	Off
? Ref. amplitude 1H	0.000 V

Physio - Signal1

1st Signal/Mode	Pulse/Trigger
Average cycle	968 ± 30 ms
Average cycle	982 ± 30 ms
Acquisition window	650 ms
Trigger pulse	1
Trigger delay	200 ms
TR	270 ms
Concatenations	12
Phases	1

Physio - PACE

Resp. control	Off
Concatenations	12

Diff - Neuro

Diffusion mode	MDDW
Diff. directions	30
Diffusion Scheme	Monopolar

Diff - Neuro

Diff. weightings	1
b-value	0 s/mm²
b-value	1
Diff. weighted images	On
Trace weighted images	Off
ADC maps	Off
FA maps	Off
Mosaic	Off
Tensor	Off
Noise level	0

Diff - Body

Diffusion mode	MDDW
Diff. directions	30
Diffusion Scheme	Monopolar
Diff. weightings	1
b-value	0 s/mm²
b-value	1
Diff. weighted images	On
Trace weighted images	Off
ADC maps	Off
Exponential ADC Maps	Off
FA maps	Off
Invert Gray Scale	Off
Calculated Image	Off
b-Value >=	0 s/mm²
Noise level	0

Diff - Composing

Inline Composing	Off
Distortion Corr.	Off

Sequence - Part 1

Introduction	On
Optimization	None
Multi-slice mode	Interleaved
Free echo spacing	Off
Echo spacing	0.93 ms
Bandwidth	1240 Hz/Px

Sequence - Part 2

EPI factor	50
RF pulse type	Normal
Gradient mode	Performance
Excitation	ZOOMit

Sequence - pTX Pulses

pTX Pulse	1
TX acceleration	1.0
