# Supplementary material for: Multi-parametric quantitative in vivo spinal cord MRI with unified signal readout and image denoising
Source: Neuroimage. 2020 Aug 15;217:116884. doi: 10.1016/j.neuroimage.2020.116884 (PMC7378937; doi:10.1016/j.neuroimage.2020.116884)
Supplement: S3: SVD of synthetic signals corrupted with Gaussian noise [file mmc4.pdf]

Supplementary material S3
Singular value decomposition of synthetic signals corrupted with Gaussian noise

This document reports plots obtained from the singular value decomposition of a matrix obtained stacking voxels from one synthetic spinal cord spinal cord slice along rows, with measurements along columns.

The document contains 5 figures, referring to SNR levels of 10, 15, 20, 30 and 40. Each figure contains 8 panels, referring to 8 different denoising strategies (left: denoising of concatenation of multi-contrast MRI signals; right: denoising of each MRI contrast individually), i.e. A): joint denoising of all contrasts; B): denoising of diffusion-weighted imaging (DWI) alone; C): joint denoising of DWI and quantitative magnetisation transfer (qMT); D): denoising of qMT alone; E): joint denoising of DWI and inversion recovery (IR); F) denoising of IR alone; G): joint denoising of DWI and the multi-TE acquisition (mTE); H): denoising of mTE alone.

Finally, in each plot there are 3 dotted curves, respectively referring to singular values of noise-free signals, noisy signals and denoised signals. Additionally, machine precision with respect to noisy signals is also reported as a dashed curve.

This supplementary document refers to Gaussian noise.

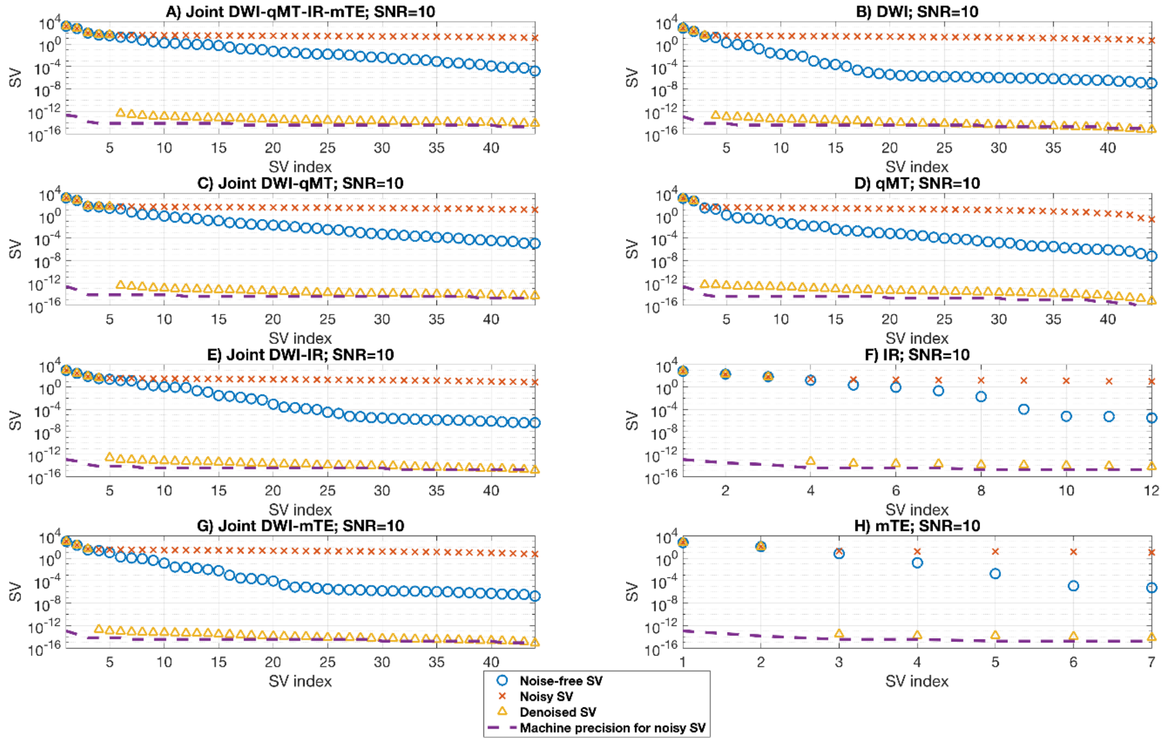

Fig. S3.1. Singular value decomposition of noise-free, noisy and denoised signals for an SNR of 10.

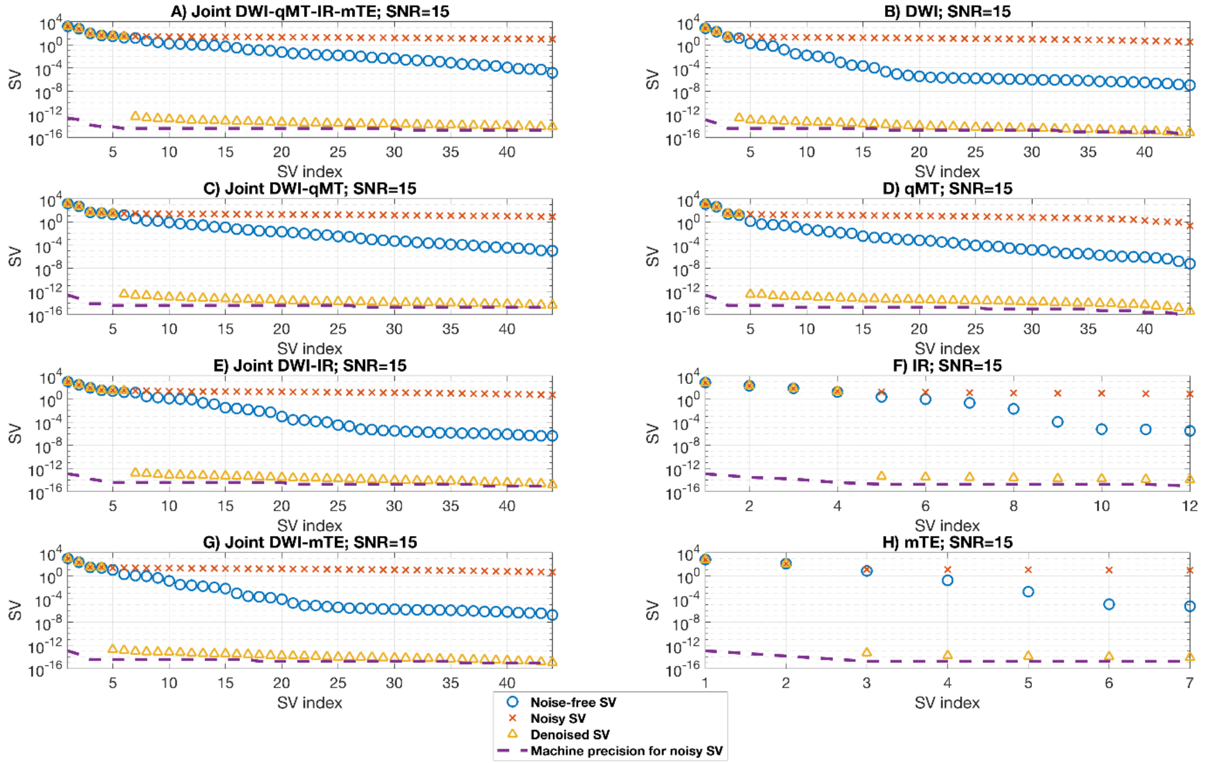

Fig. S3.2. Singular value decomposition of noise-free, noisy and denoised signals for an SNR of 15.

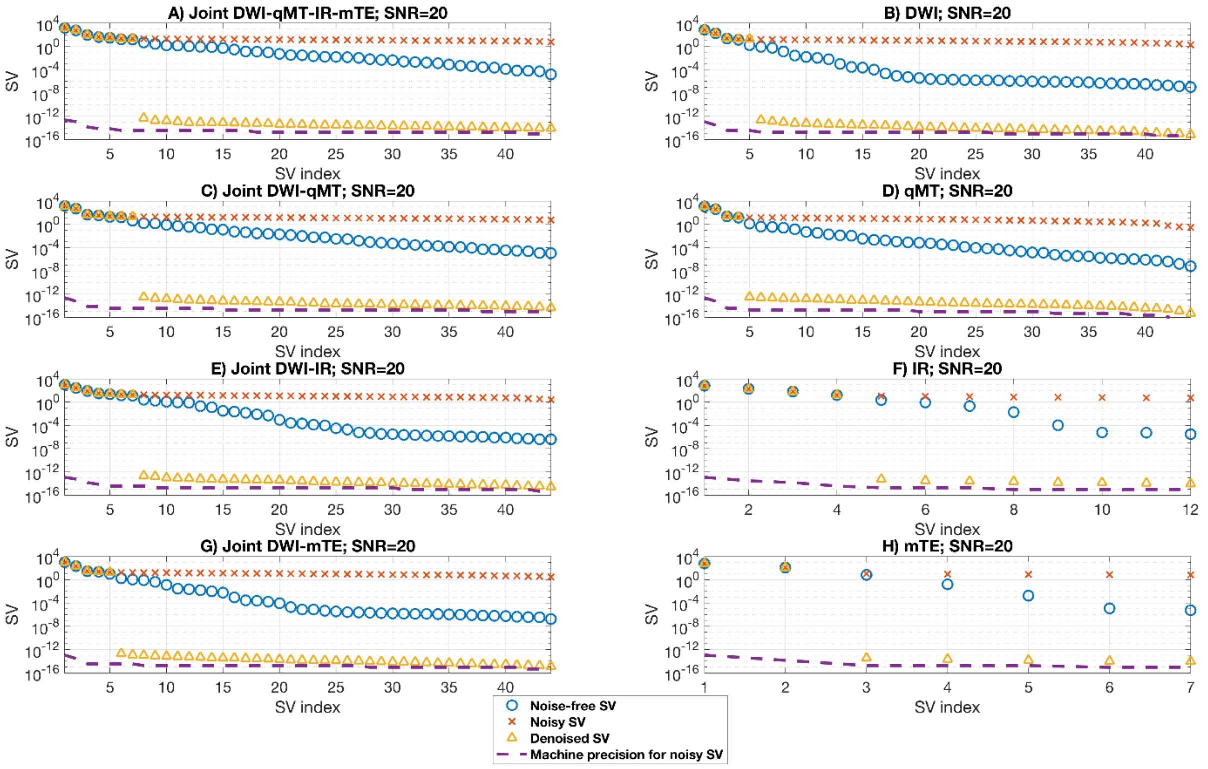

Fig. S3.3. Singular value decomposition of noise-free, noisy and denoised signals for an SNR of 20.

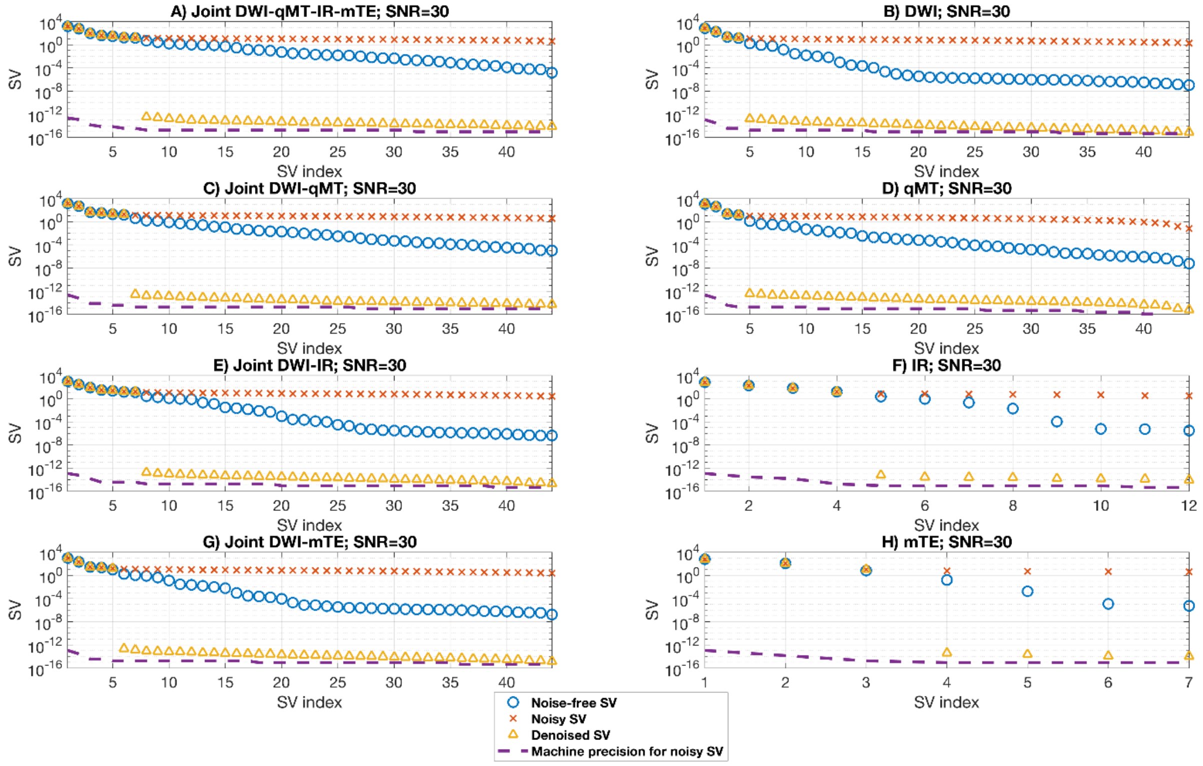

Fig. S3.4. Singular value decomposition of noise-free, noisy and denoised signals for an SNR of 30.

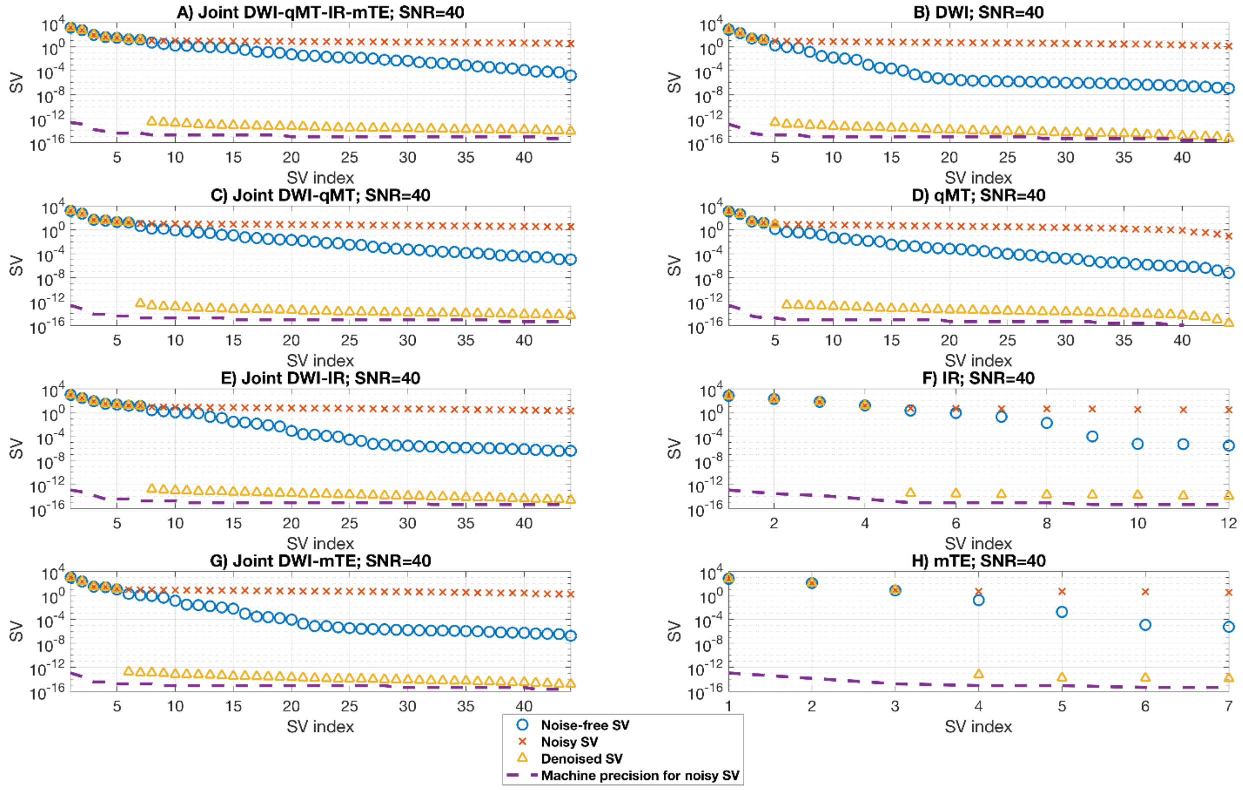

Fig. S3.5. Singular value decomposition of noise-free, noisy and denoised signals for an SNR of 40.
