# Supplementary material for: Multi-parametric quantitative in vivo spinal cord MRI with unified signal readout and image denoising
Source: Neuroimage. 2020 Aug 15;217:116884. doi: 10.1016/j.neuroimage.2020.116884 (PMC7378937; doi:10.1016/j.neuroimage.2020.116884)
Supplement: S4: Simulations considering Rician noise [file mmc5.pdf]

Supplementary material S4
Simulations considering Rician noise

This document reports results from simulations performed by corrupting synthetic, noise-free magnitude signals with Rician noise at different SNR levels.

Supplementary figures S4.1 and S4.2 in this document correspond to figures 1 and 2 of the main manuscript, but they are obtained considering Rician rather than Gaussian noise (i.e. accuracy and precision of denoising; examples of denoising input/output matrices against ground truth, with corresponding singular values (SV)).

Supplementary figures S4.3 to S4.7 correspond to the figures in Supplementary Material S3, but also consider Rician rather than Gaussian noise. They refer to the singular value decomposition of a matrix obtained from a synthetic spinal cord slice. In figures S4.3 to S4.7, machine precision is reported for completeness.

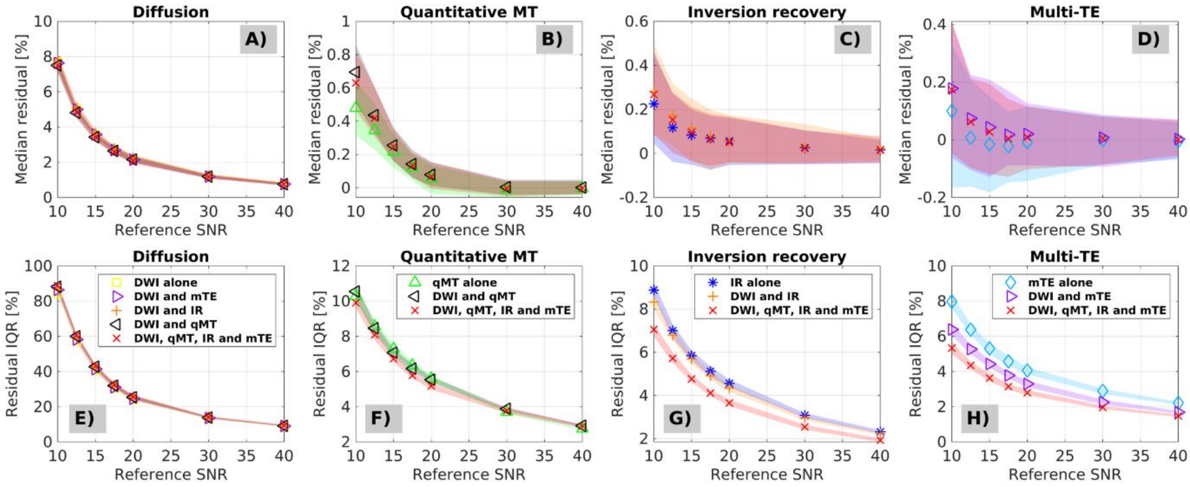

Fig. S4.1. Accuracy and precision of MP-PCA in estimating the underlying ground truth signal. Please also refer to figure 1 of the main manuscript for detailed description of each panel in the artwork.

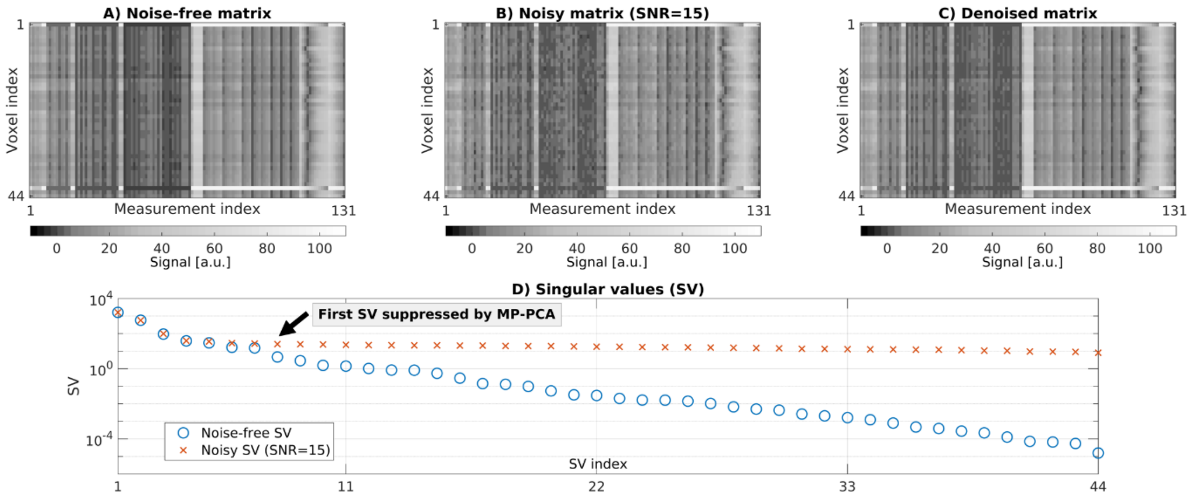

Fig. S4.2. Top: examples of noise-free (A), noisy (B) and denoised (C) matrices from the synthetic spinal cord phantom. Bottom (D): SV decomposition of the noise-free and noisy matrices shown in A and B, alongside MP-PCA cut off (i.e. edge of noisy SVs MP distribution). MP-PCA nullifies all SVs starting from the cut off to the right, while it preserves those to the left. The figure reports results from the simulation conducted with Rician noise at an SNR of 15, and considers joint denoising of the whole set of 131 MRI measurements from one spinal cord slice made of 44 voxels (concatenation of DW, qMT, IR and mTE imaging). Note that one additional component is kept as compared to the equivalent case with additive Gaussian noise (see Fig. 2 of main manuscript), likely an effect of noise floor bias.

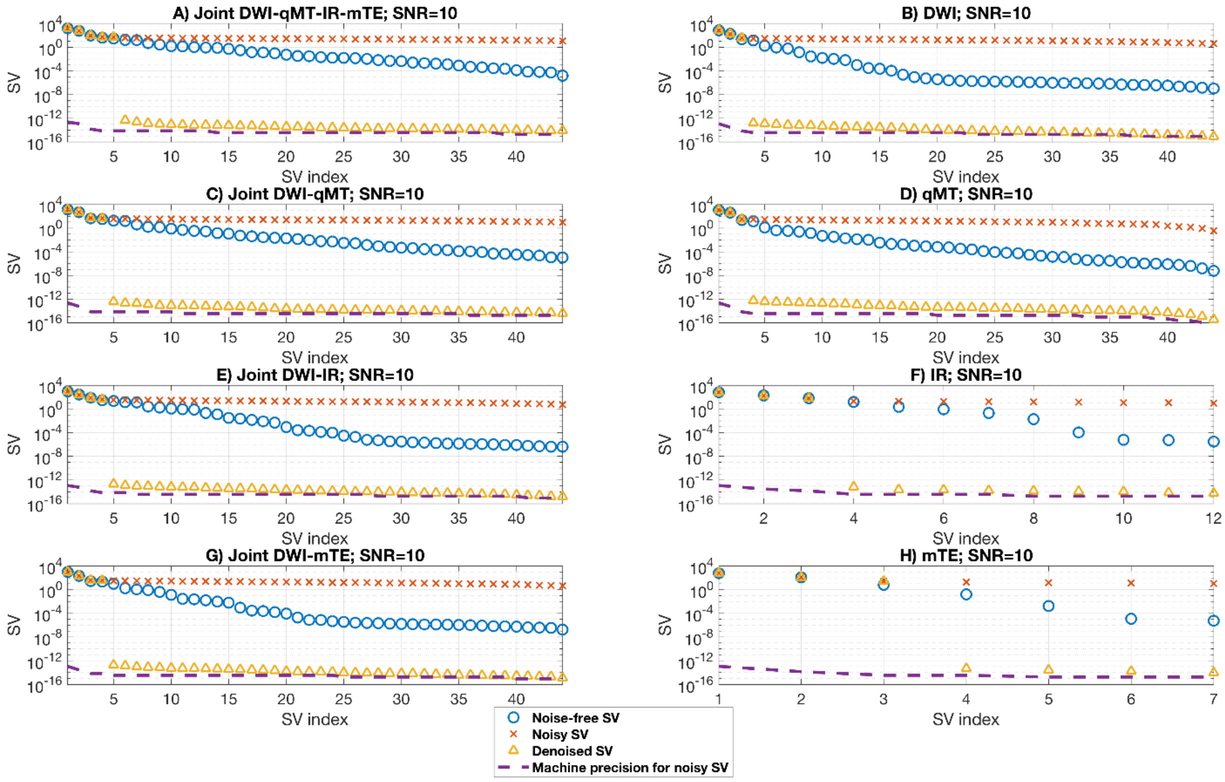

Fig. S4.3. Singular value decomposition of noise-free, noisy and denoised signals for an SNR of 10. Please also refer to Supplementary Material S3.

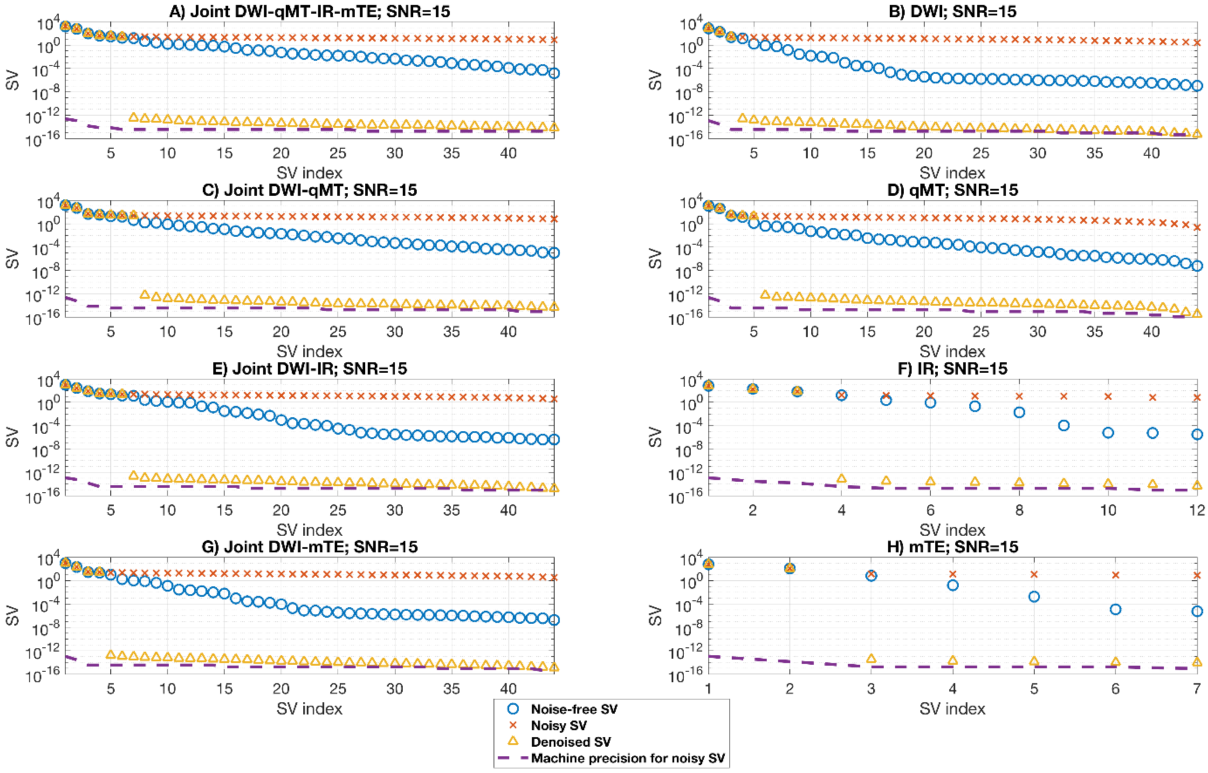

Fig. S4.4. Singular value decomposition of noise-free, noisy and denoised signals for an SNR of 15. Please also refer to Supplementary Material S3.

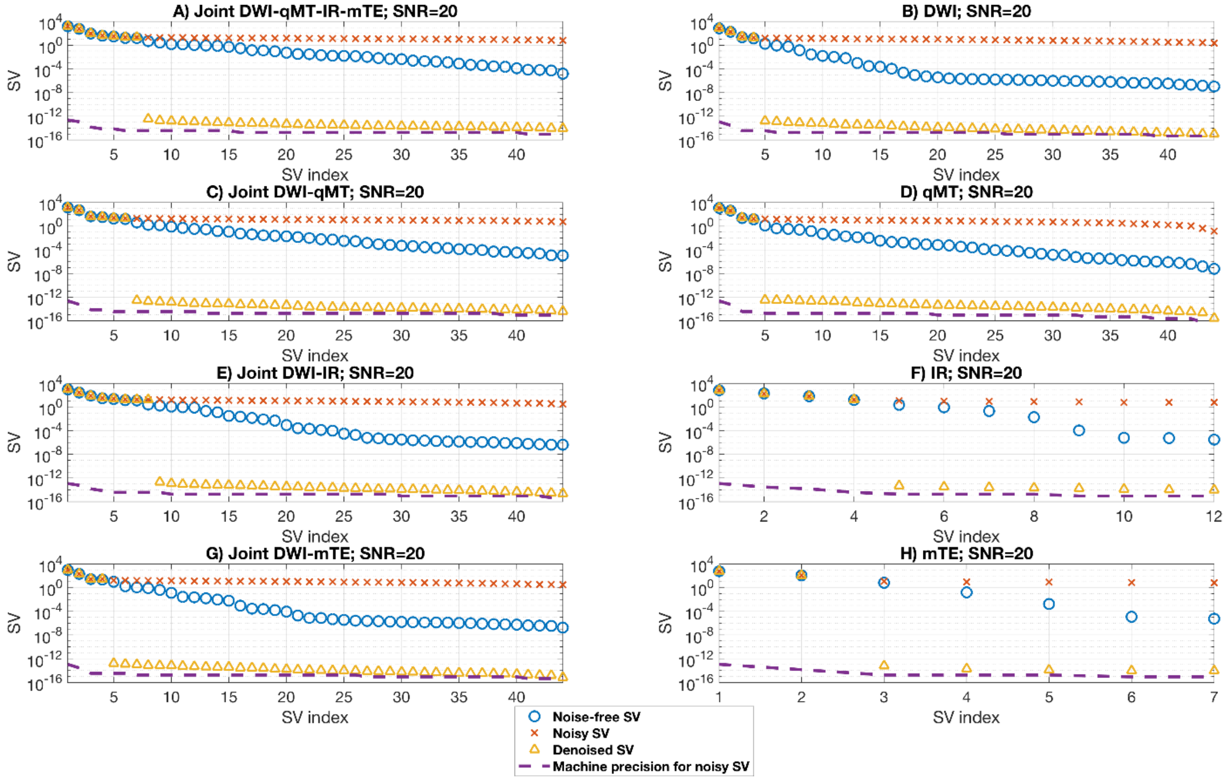

Fig. S4.5. Singular value decomposition of noise-free, noisy and denoised signals for an SNR of 20. Please also refer to Supplementary Material S3.

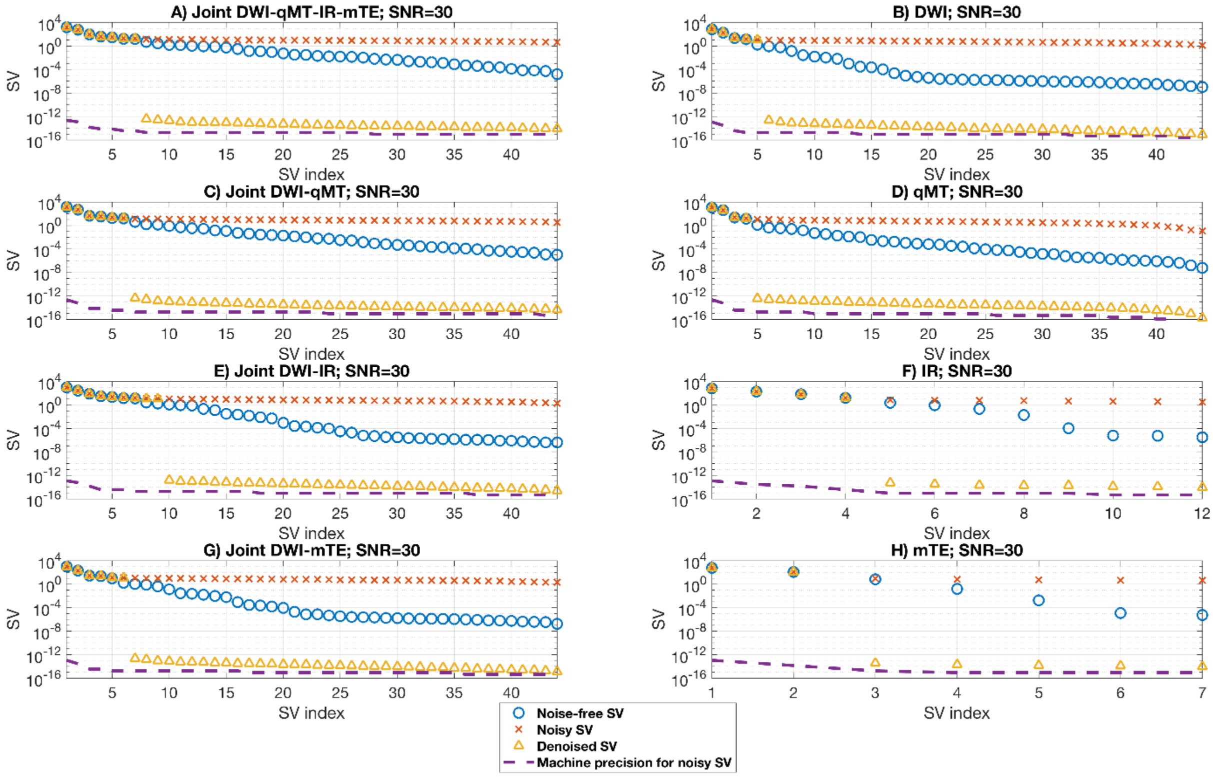

Fig. S4.6. Singular value decomposition of noise-free, noisy and denoised signals for an SNR of 30. Please also refer to Supplementary Material S3.

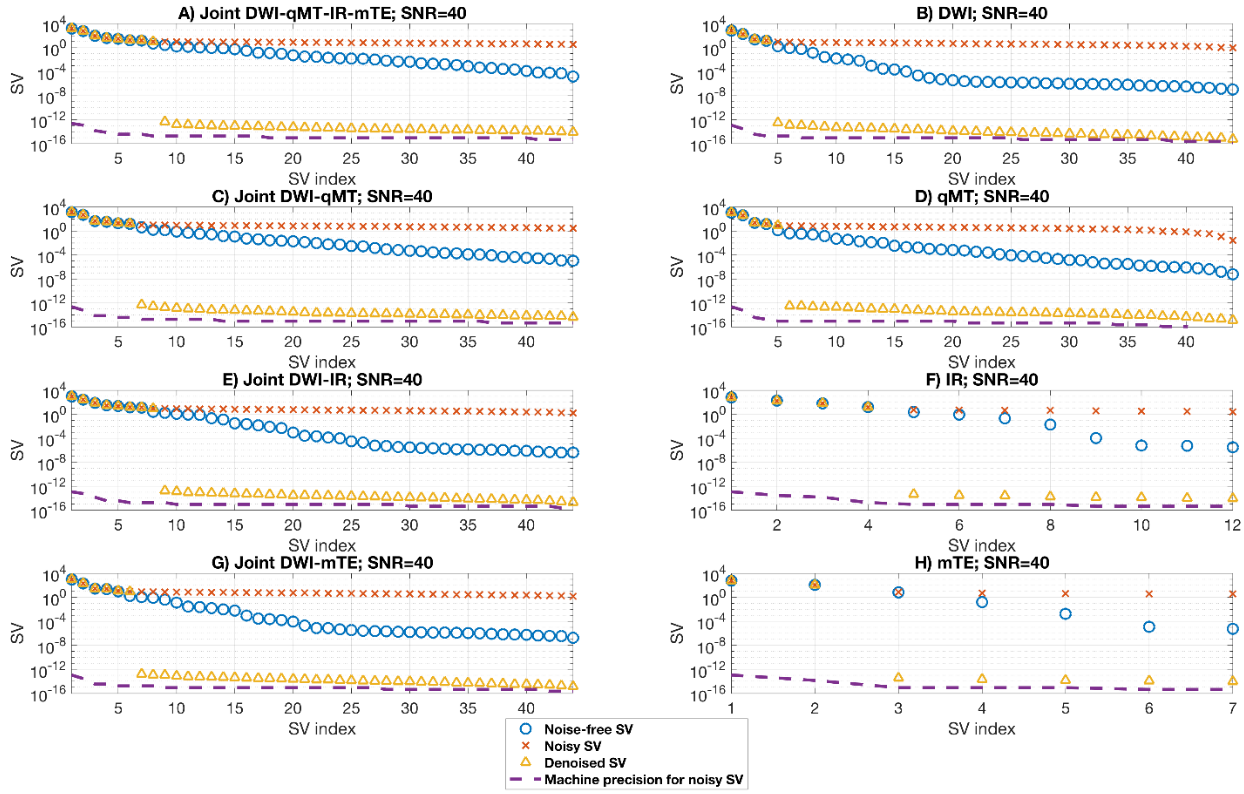

Fig. S4.7. Singular value decomposition of noise-free, noisy and denoised signals for an SNR of 40. Please also refer to Supplementary Material S3.
