# Supplementary material for: Multi-parametric quantitative in vivo spinal cord MRI with unified signal readout and image denoising
Source: Neuroimage. 2020 Aug 15;217:116884. doi: 10.1016/j.neuroimage.2020.116884 (PMC7378937; doi:10.1016/j.neuroimage.2020.116884)
Supplement: S5: Distribution of residuals [file mmc6.pdf]

Supplementary material S5
Distribution of residuals

This document illustrates distribution of normalised denoising residuals (i.e. denoising output – denoising input, normalised by the estimated noise σ). Distributions are plotted in the semi-log scales against the squared residuals. In this plot, a Gaussian distribution appears as a straight line of slope –½ (labelled as "Reference" in the legends).

The document reports distributions from both simulated and in vivo scans.

Simulations

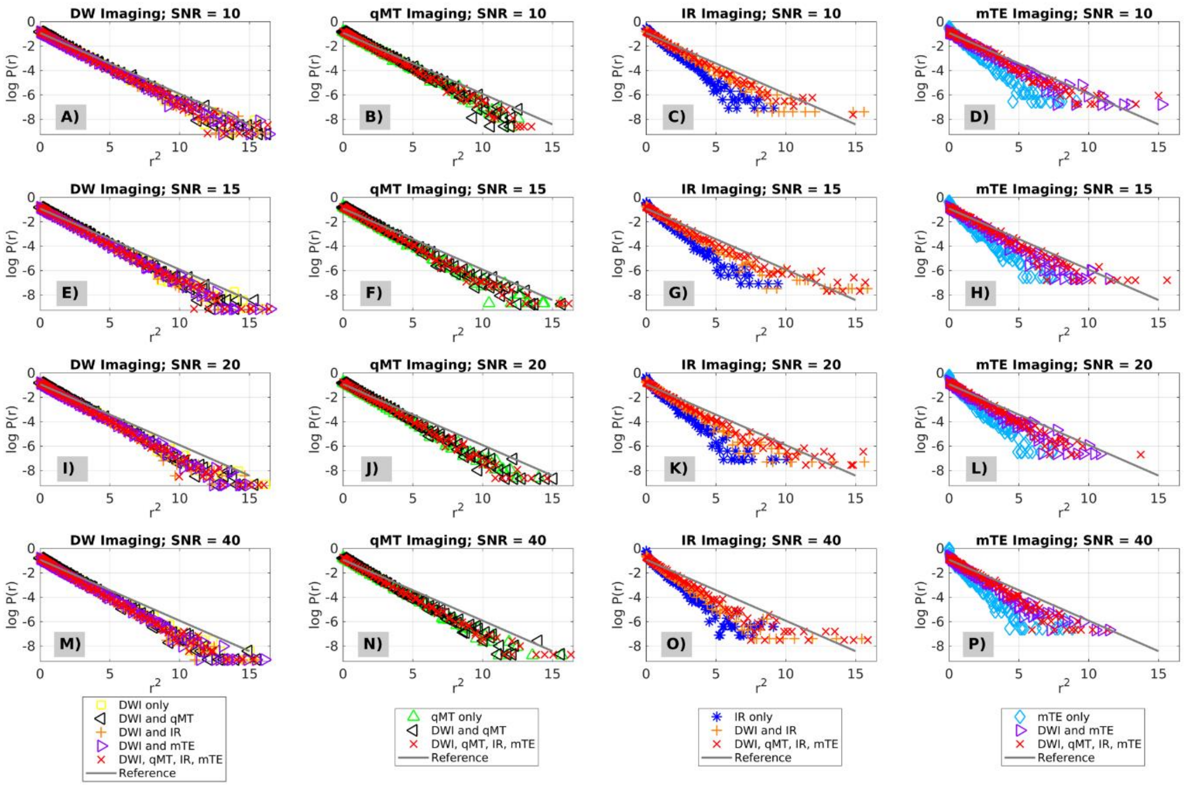

Fig. S5.1. Examples of distributions of residuals from simulations (Gaussian noise).

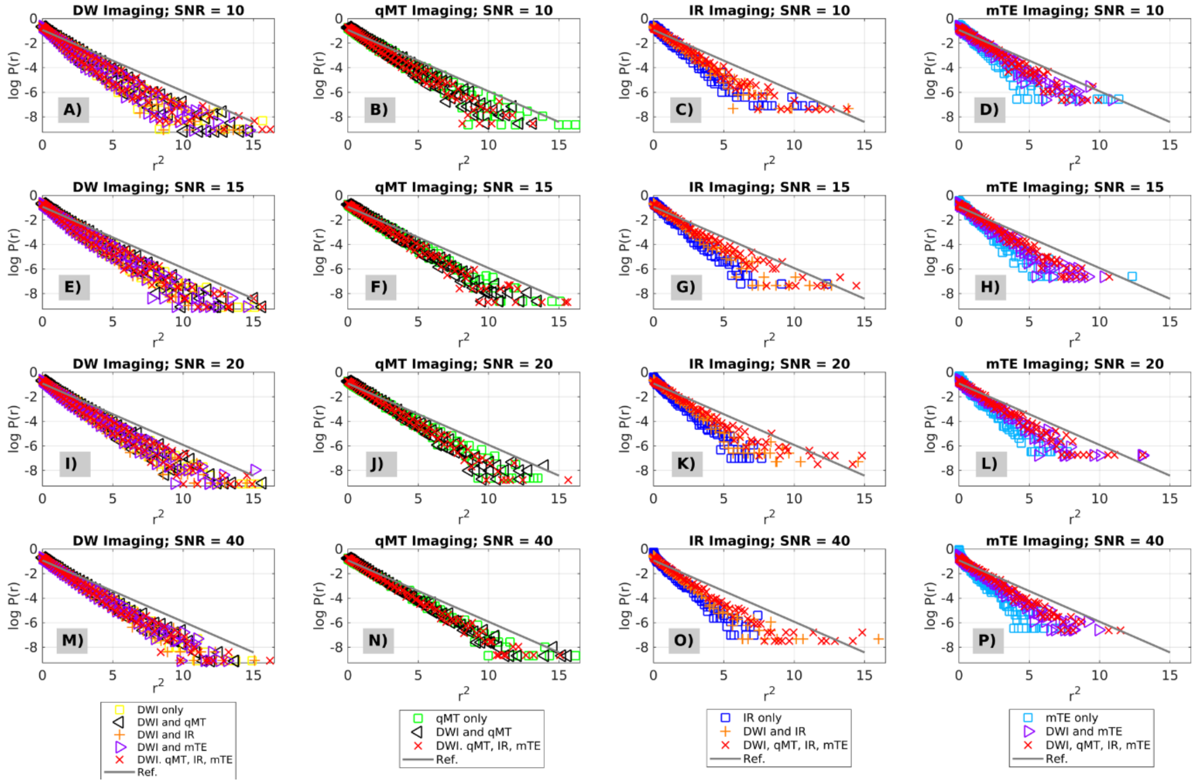

Fig. S5.2. Examples of distributions of residuals from simulations (Rician noise).

In vivo

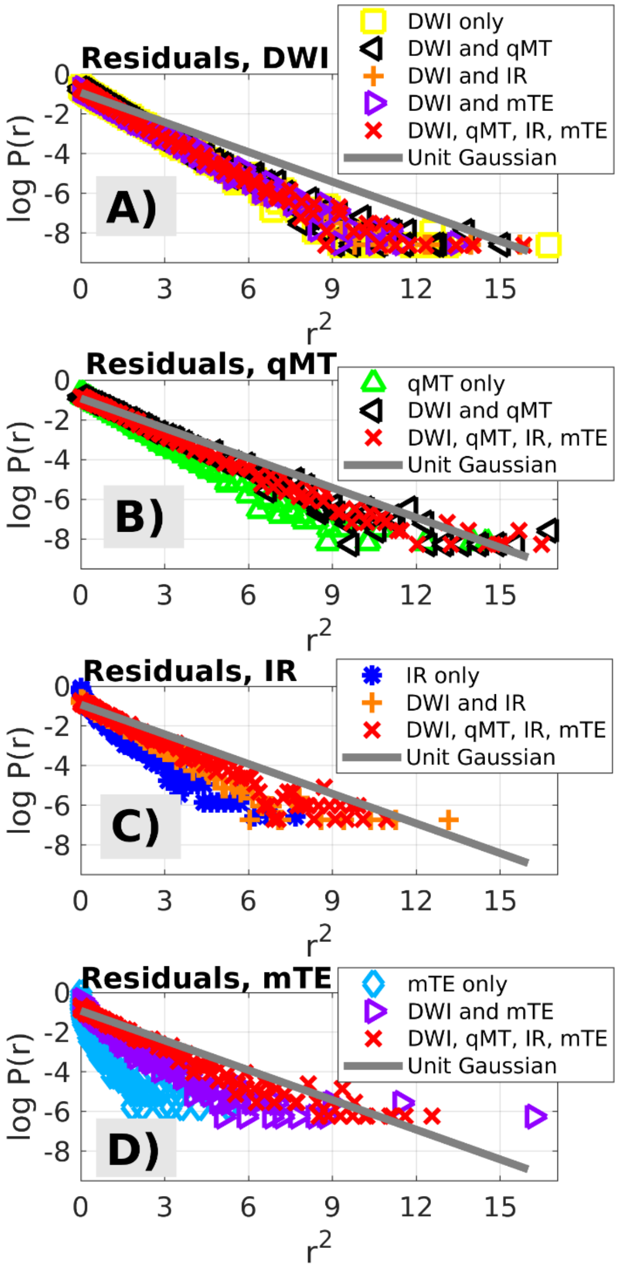

Fig. S5.3. Examples of distributions of residuals from in vivo data (vendor 1, London).

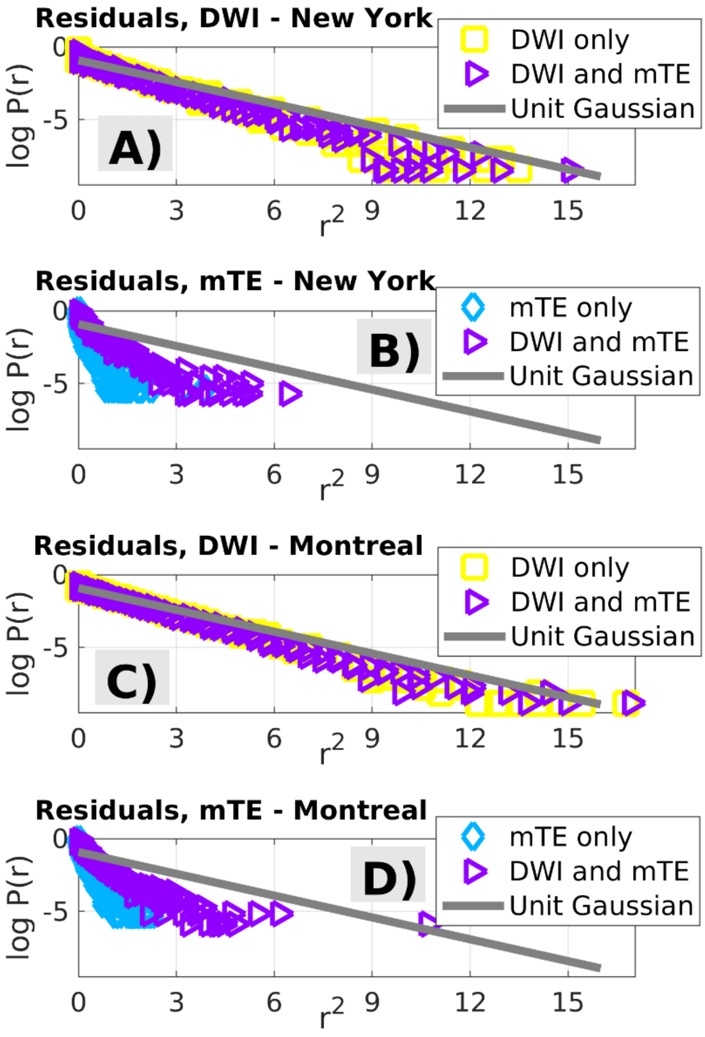

Fig. S5.4. Examples of distributions of residuals from in vivo data (vendor 2, two scanners – New York City and Montreal).
