# Supplementary material for: Multi-parametric quantitative in vivo spinal cord MRI with unified signal readout and image denoising
Source: Neuroimage. 2020 Aug 15;217:116884. doi: 10.1016/j.neuroimage.2020.116884 (PMC7378937; doi:10.1016/j.neuroimage.2020.116884)
Supplement: S6: Difference maps [file mmc7.pdf]

# Supplementary material S6
# Difference maps

This document reports examples of voxel-wise differences in quantitative maps obtained with and without denoising. Supplementary figures S6.1, S6.2 and S6.3 respectively correspond to figures 5, 6 and 7 of the main manuscript.

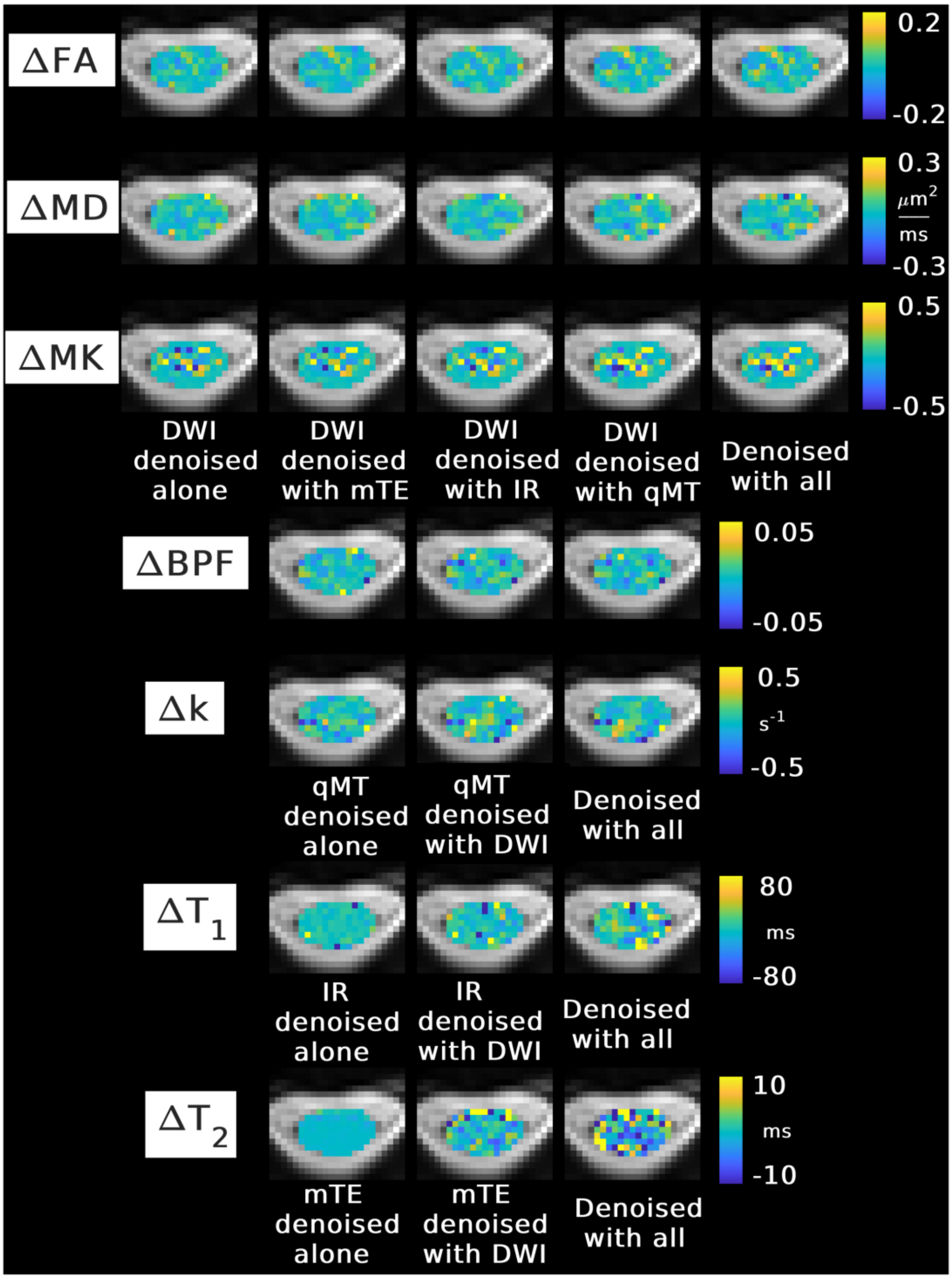


**Fig. S6.1.** Difference maps corresponding to figure 5 (vendor 1). Each plot shows the difference between a map obtained after denoising and the same map obtained without denoising (Δ = denoising – no denoising).

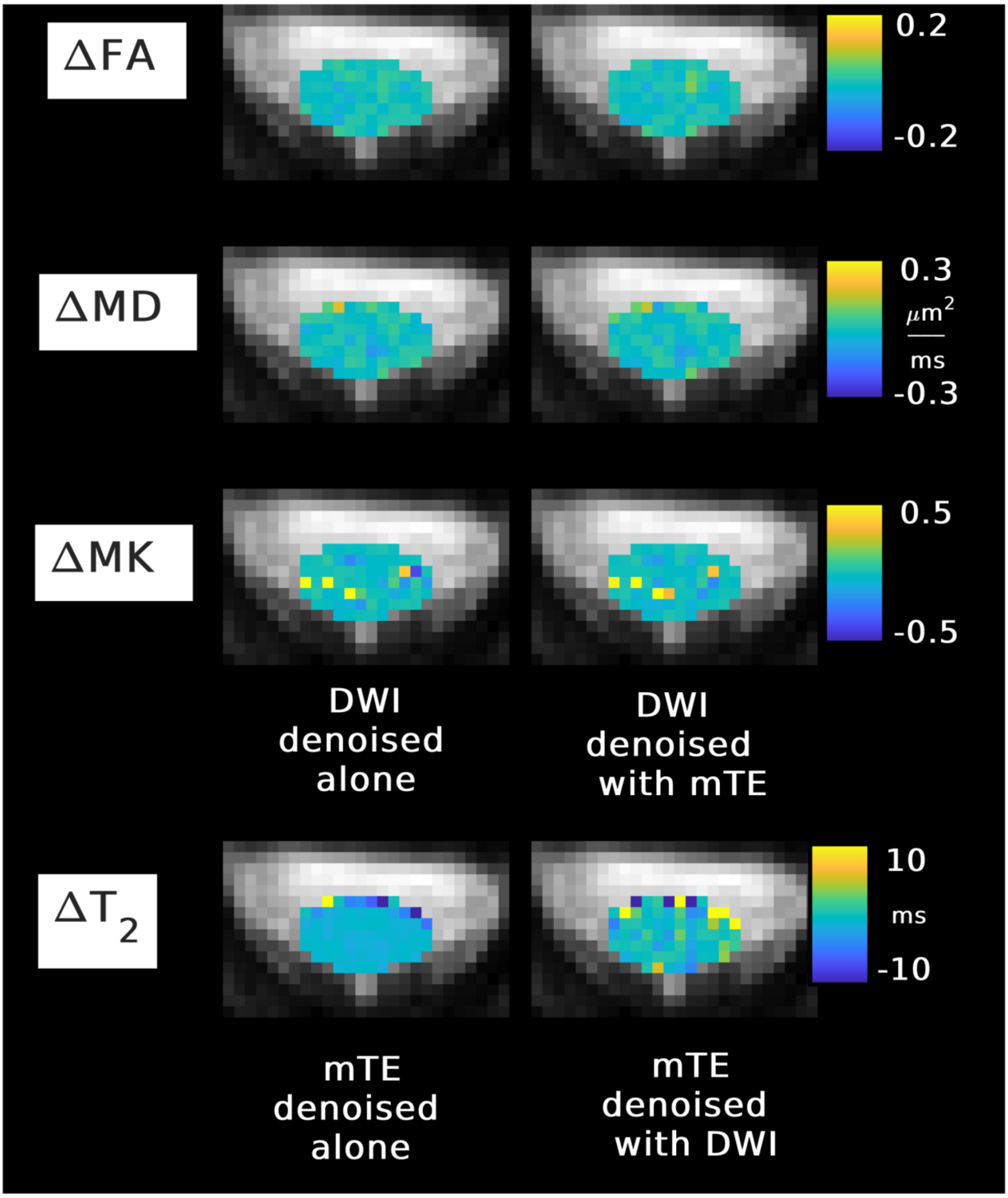


**Fig. S6.2.** Difference maps corresponding to figure 6 (vendor 2, New York). Each plot shows the difference between a map obtained after denoising and the same map obtained without denoising (Δ = denoising – no denoising).

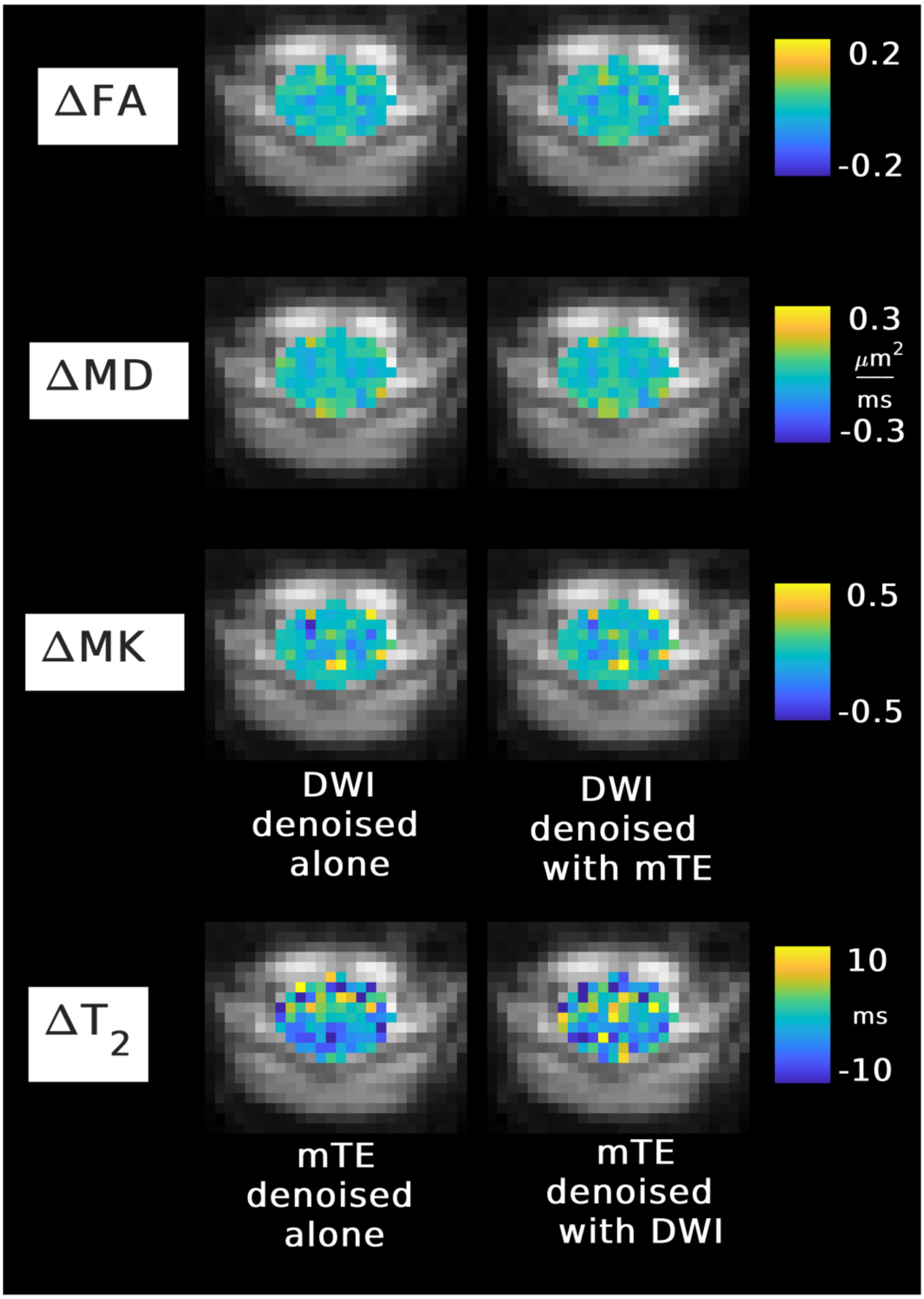


**Fig. S6.3.** Difference maps corresponding to figure 7 (vendor 2, Montreal). Each plot shows the difference between a map obtained after denoising and the same map obtained without denoising (Δ = denoising – no denoising).
