# Supplementary material for: Multi-parametric quantitative in vivo spinal cord MRI with unified signal readout and image denoising
Source: Neuroimage. 2020 Aug 15;217:116884. doi: 10.1016/j.neuroimage.2020.116884 (PMC7378937; doi:10.1016/j.neuroimage.2020.116884)
Supplement: S7: Contrast-to-noise ratio [file mmc8.pdf]

Supplementary material S7
Contrast-to-noise ratio

This document reports grey/white matter contrast-to-noise ratio (CNR) for all parametric maps and all denoising strategies. The two supplementary tables below show mean and standard deviation (within brackets) of CNR across subjects and scans for both vendors. An increase of mean CNR greater than 5% compared to the case with no denoising is labelled in bold font and green shadowing. A decrease of mean CNR greater than 5% compared to the case with no denoising is labelled in bold font and red shadowing.

Supplementary table S7.1: grey/white matter CNR for vendor 1

	No denoising	Individual denoising	Joint denoising DWI-mTE	Joint denoising DWI-IR	Joint denoising DWI-qMT	Joint denoising of all
**FA CNR (DWI)**	0.20 (0.2)	**0.22 (0.21)**	**0.24 (0.22)**	0.21 (0.21)	**0.22 (0.20)**	0.21 (0.19)
**MD CNR (DWI)**	0.40 (0.07)	0.38 (0.07)	0.39 (0.07)	0.39 (0.07)	**0.37 (0.08)**	**0.37 (0.08)**
**MK CNR (DWI)**	0.13 (0.08)	**0.15 (0.07)**	**0.17 (0.09)**	**0.17 (0.09)**	0.13 (0.09)	0.13 (0.08)
**BPF CNR (qMT)**	0.10 (0.08)	**0.12 (0.07)**	NA	NA	0.10 (0.08)	0.10 (0.07)
**k CNR (qMT)**	0.24 (0.09)	0.24 (0.11)	NA	NA	0.24 (0.11)	0.24 (0.10)
**T1 CNR (IR)**	0.12 (0.13)	0.12 (0.12)	NA	0.12 (0.13)	NA	0.12 (0.14)
**T2 CNR (mTE)**	0.34 (0.17)	0.34 (0.16)	0.34 (0.17)	NA	NA	**0.36 (0.18)**

Supplementary table S7.2: grey/white matter CNR for vendor 2

	No denoising	Individual denoising	Joint denoising DWI-mTE
**FA CNR (DWI)**	0.47 (0.16)	0.47 (0.14)	0.49 (0.14)
**MD CNR (DWI)**	0.24 (0.09)	0.25 (0.09)	0.25 (0.09)
**MK CNR (DWI)**	0.09 (0.05)	**0.11 (0.10)**	**0.13 (0.08)**
**T2 CNR (mTE)**	0.45 (0.13)	0.44 (0.12)	0.46 (0.12)
