# Supplementary material for: Multi-parametric quantitative in vivo spinal cord MRI with unified signal readout and image denoising
Source: Neuroimage. 2020 Aug 15;217:116884. doi: 10.1016/j.neuroimage.2020.116884 (PMC7378937; doi:10.1016/j.neuroimage.2020.116884)
Supplement: S8: Estimated noise levels [file mmc9.pdf]

Supplementary material S8
Estimated noise levels

Here we compare MP-PCA estimates of noise levels when different denoising strategies are followed. Both simulations and in vivo cases are shown.

Simulations

In the following supplementary figures, we scatter the estimated noise standard deviation obtained from denoising the DWI against the values obtained by denoising the other modalities, either alone or jointly with DWI (Gaussian noise case).

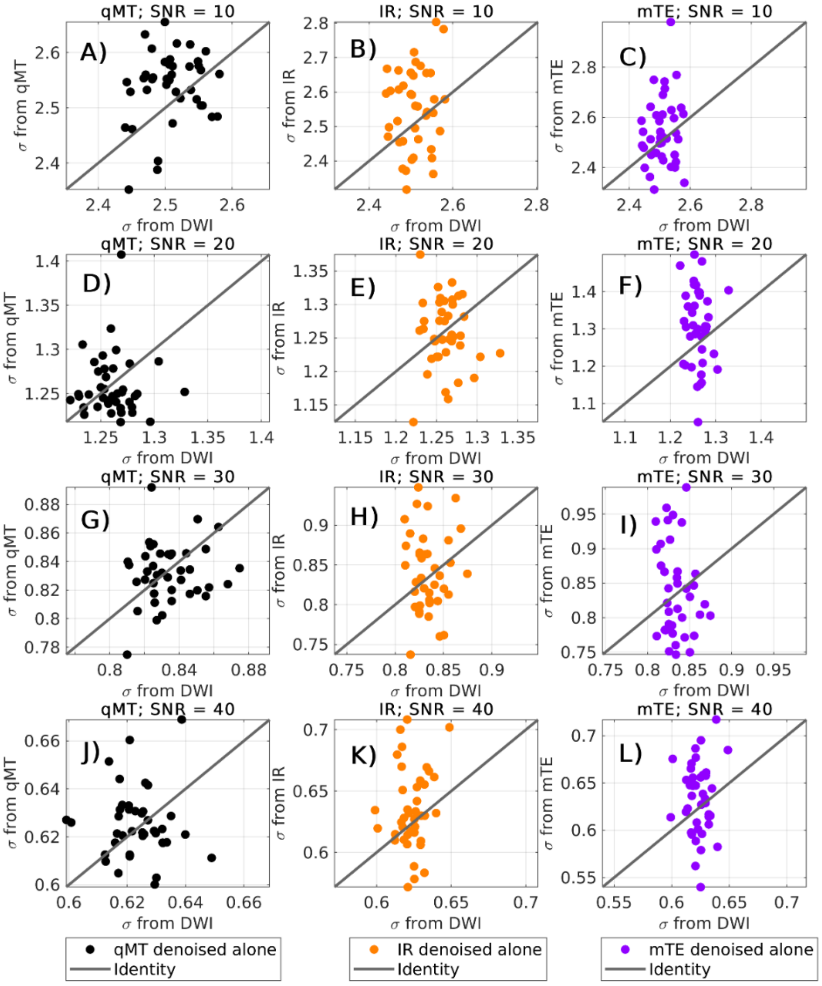

Fig. S8.1. Estimated noise standard deviation σ obtained denoising each modality independently. Modalities are: diffusion-weighted imaging (DWI); quantitative magnetisation transfer (qMT); inversion recovery (IR); multi-echo time (mTE). Each plot scatters values of σ obtained from qMT (left), IR (middle) or mTE (right), reported on the y-axis, against σ provided by denoising DWI alone (x-axis). Different rows shows different signal-to-noise ratios (SNRs) (top to bottom: SNR of 10, 20, 30, 40).

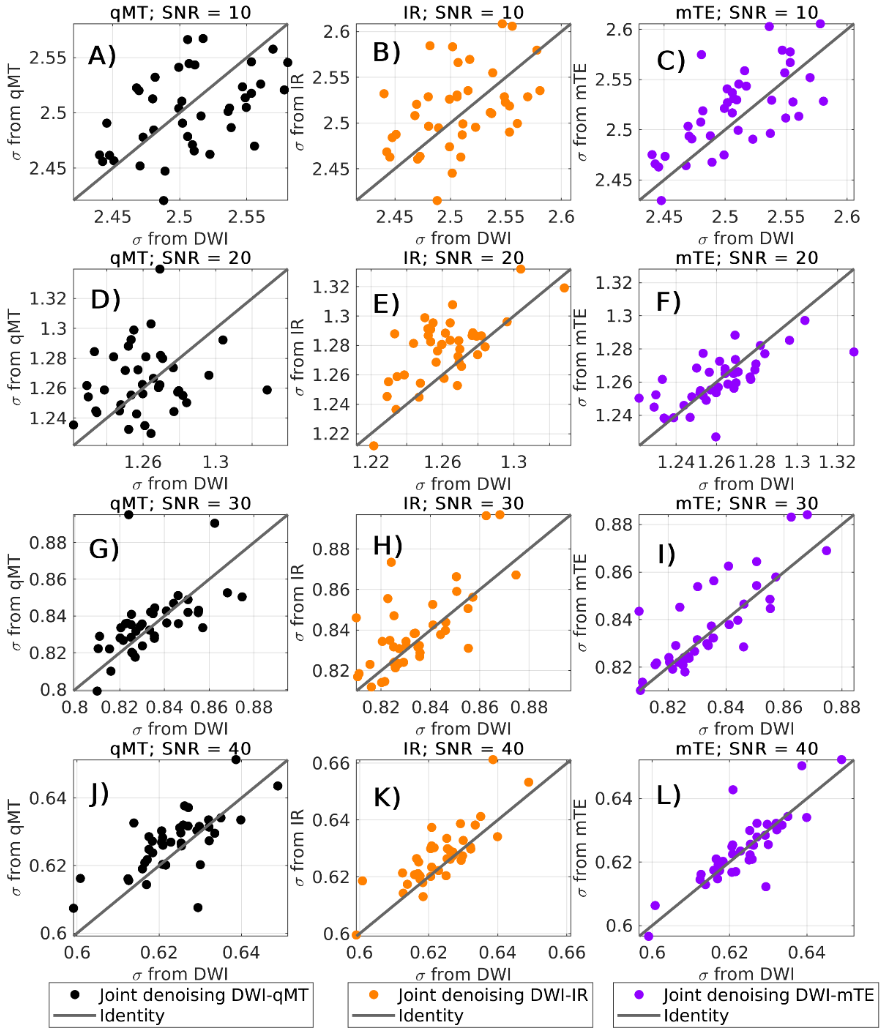

Fig. S8.2. Estimated noise standard deviation σ obtained denoising each of quantitative magnetisation transfer (qMT), inversion recovery (IR) and multi-echo time (mTE) jointly with diffusion-weighted imaging (DWI). Each plot scatters values of σ obtained from joint denoising DWI-qMT (left), DWI-IR (middle) or DWI-mTE (right), reported on the y-axis, against σ provided by denoising DWI alone (x-axis). Different rows shows different signal-to-noise ratio (SNR) levels (from top to bottom: SNR of 10, 20, 30, 40).

In vivo

In the following supplementary figures, we scatter the estimated noise standard deviation obtained from denoising the DWI against the values obtained by denoising the other modalities, either alone or jointly with DWI. Moreover, we report the scatter plots with and without noise floor mitigation, and for the two vendors.

Results are shown in both cases when denoised images and estimated noise level are corrected for Rician noise bias with the method of moments [1] and whey they are not.

[1] Koay, C.G., Basser, P.J., 2006. Analytically exact correction scheme for signal extraction from noisy magnitude MR signals. J of Magn Res 179, 317-322.

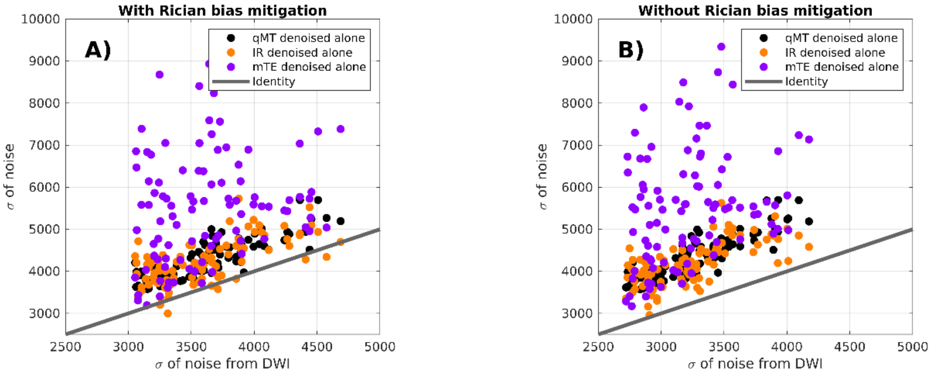

Fig. S8.3. Estimated noise standard deviation for vendor 1 (Philips Achieva) obtained denoising each modality (qMT, IR and mTE) independently. Values are scattered against the noise level estimated from the DWI alone. In panel A) (left), the estimated noise level has been corrected for Rician bias, while in panel B) (to the right) this correction has not been performed. When generating the plots, data from all scans of all subjects have been pooled together.

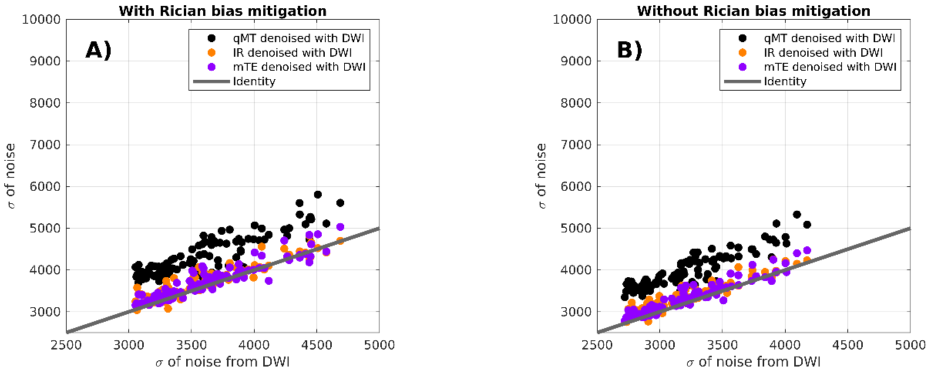

Figure S8.4. Estimated noise standard deviation for vendor 1 (Philips Achieva) obtained denoising each modality (qMT, IR and mTE) jointly with DWI. Values are scattered against the noise level estimated from the DWI alone. In panel A) (left), the estimated noise level has been corrected for Rician bias, while in panel B) (to the right) this correction has not been performed. When generating the plots, data from all scans of all subjects have been pooled together.

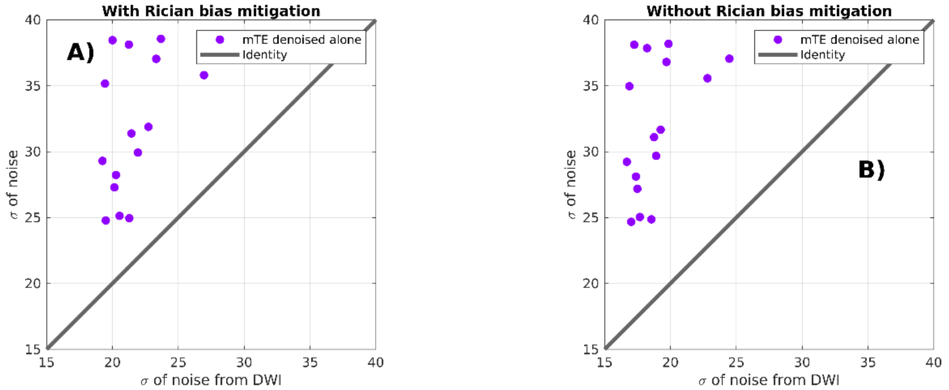

Fig. S8.5. Estimated noise standard deviation for vendor 2 (two Siemens Prisma located in New York, USA and Montreal, Canada) obtained denoising mTE independently from DWI. Values are scattered against the noise level estimated from the DWI alone. In panel A) (left), estimated noise level has been corrected for Rician bias, while in panel B) (to the right) this correction has not been performed. When generating the plots, data from all scans of all systems have been pooled together.

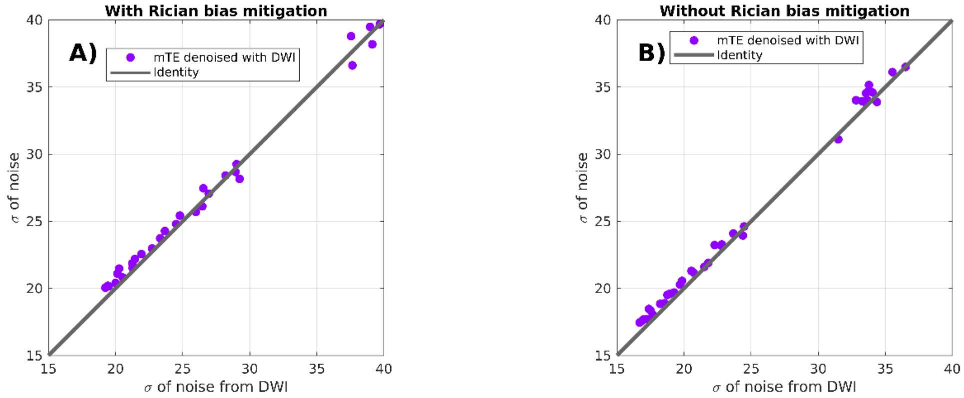

Fig. S8.6. Estimated noise standard deviation for vendor 2 (two Siemens Prisma located in New York, USA and Montreal, Canada) obtained denoising mTE jointly with DWI. Values are scattered against the noise level estimated from the DWI alone. In panel A) (left), the estimated noise level has been corrected for Rician bias, while in panel B) (to the right) this correction has not been performed. When generating the plots, data from all scans of all systems have been pooled together.
